# Neonatal and under-five mortality rate in Indian districts with reference to Sustainable Development Goal 3: An analysis of the National Family Health Survey of India (NFHS), 2015–2016

**DOI:** 10.1371/journal.pone.0201125

**Published:** 2018-07-30

**Authors:** Jayanta Kumar Bora, Nandita Saikia

**Affiliations:** 1 Indian Institute of Dalit Studies, New Delhi, India; 2 Vienna University of Economics and Business (WU), Demography Group/Wittgenstein Centre for Demography and Global Human Capital (IIASA, VID/ÖAW, WU), Vienna, Austria; 3 International Institute for Applied Systems Analysis, Laxenburg, Austria; 4 Center for the Study of Regional Development, School of Social Science, SSS III, Jawaharlal Nehru University, New Delhi, India; University of Miami, UNITED STATES

## Abstract

**Background and objective:**

India contributes the highest global share of deaths among the under-fives. Continuous monitoring of the reduction in the under-five mortality rate (U5MR) at local level is thus essential to set priorities for policy-makers and health professionals. In this study, we aimed to provide an update on district-level disparities in the neonatal mortality rate (NMR) and the U5MR with special reference to Sustainable Development Goal 3 (SDG3) on preventable deaths among new-borns and children under five.

**Data and methods:**

We used recently released population-based cross-sectional data from the National Family Health Survey (NFHS) conducted in 2015–2016. We used the synthetic cohort probability approach to analyze the full birth history information of women aged 15–49 to estimate the NMR and U5MR for the ten years preceding the survey.

**Results:**

Both the NMR and U5MR vary enormously across Indian districts. With respect to the SDG3 target for 2030 for the NMR and the U5MR, the estimated NMR for India for the period studied is about 2.4 times higher, while the estimated U5MR is about double. At district level, while 9% of the districts have already reached the NMR targeted in SDG3, nearly half (315 districts) are not likely to achieve the 2030 target even if they realize the NMR reductions achieved by their own states between the last two rounds of National Family Health Survey of India. Similarly, less than one-third of the districts (177) of India are unlikely to achieve the SDG3 target on the U5MR by 2030. While the majority of high-risk districts for the NMR and U5MR are located in the poorer states of north-central and eastern India, a few high-risk districts for NMR also fall in the rich and advanced states. About 97% of districts from Chhattisgarh and Uttar Pradesh, for example, are unlikely to meet the SDG3 target for preventable deaths among new-borns and children under age five, irrespective of gender.

**Conclusions:**

To achieve the SDG3 target on preventable deaths by 2030, the majority of Indian districts clearly need to make a giant leap to reduce their NMR and U5MR.

## Introduction

India has a highly significant role to play in global efforts to end the preventable death of newborns and children under the age of five, given that it has the highest number of deaths among these two groups in the world. According to various rounds of the National Family Health Survey (NFHS) data, the under-five mortality rate (U5MR) has declined by a little more than half in the past 23 years, namely, from 109 deaths (per 1000 live births) in c.1990 (five years prior to the 1992–1993 NFHS) to 50 deaths (per 1000 live births) in c. 2013 (five years prior to the 2015–2016 NFHS) [1]. The reduction in the neonatal mortality rate (NMR) was only 19 units (per 1000 live births) for the same period [1,2]. Thus, if India experiences a similar decline in the U5MR and the NMR in the next 12 years, it is very likely to meet the target set by the Sustainable Development Goals (SDGs) for U5MR but unlikely to meet the NMR target. As per Target 3.2 of SDG3 indicates that by 2030, seeks to end preventable deaths of newborns and children under 5 years of age, with all countries aiming to reduce neonatal mortality to at least as low as 12 per 1,000 live births and under-5 mortality to at least to as low as 25 per 1,000 live births.

Even for the U5MR, the national-level estimate masks the huge geographic diversity across Indian states and districts. Even if the current national-level U5MR were halved by 2030, the SDG3 target might still not be achieved in many districts. At the same time, unlike the Millennium Development Goals (MDGs), the SDGs are not only target-oriented, but also include all subsections of the country. The battle cry for the SDGs, “No one left behind” means that for India, the overarching goal is to reduce inequalities across gender, region, class, and caste. For a large and diverse country like India, it is thus imperative to examine the improvement in child survival at the lowest possible geographical unit by gender.

There has been a great deal of information on socioeconomic and geographical disparity in neonatal, infant, and UFM in recent years [2–12]. Only a few studies, however, have examined that disparity at below the Indian state level [4,13–15]. Ram and colleagues [13] applied the state and district mortality-rate pattern, derived from a few nationally representative surveys, to the 2012 UN sex-specific birth and mortality totals; the researchers indirectly calculated that only 37% of 597 districts were on track to achieve Millennium Development Goal (MDG) number 4 of 38 deaths per 1000 live births among the under-fives by 2015, and only around another 37% after 2020. They also found that more districts lagged behind the respective goal for NMR than for the U5MR. Gupta et al. [4] examined the spatial clustering and risk factors of infant mortality rate (IMR) across nine high focused states of India. The nine high focused states are Assam, Bihar, Chhattisgarh, Jharkhand, Madhya Pradesh, Odisha, Rajasthan, Uttarakhand and Uttar Pradesh. These states have 48.5% of India’s population and have under five and neonatal mortality higher than national level. Another study by Kumar et al [14] examined the role of socioeconomic and health-care indicators on the U5MR after controlling for bio-physical and geographical variables in nine high focused states of India. Both these studies concluded that health program initiatives play a major role in reducing child mortality. Singh et al [15] found evidence of the importance of spatial risk factors in explaining differential IMRs for 74 geographic regions of India.

These studies, however, discussed neither the district-level NMR and U5MR related to the SDG3 target on preventable deaths among newborns and children, nor the gender differential in child mortality indicators, which is an important concern of the SDGs. The present study contributes knowledge on the NMR and U5MR in India in several directions. ***First***, we used the National Family Health Survey (NFHS) conducted in 2015–2016 across all districts of India: the most recent nationally representative survey data giving the full birth history information of 699,686 women. As the full birth history information of women is the most unbiased basis on which to calculate robust mortality indicators among children, this study provides the most accurate estimates possible of the NMR and U5MR of Indian districts. We then examined the district level disparity in NMR and U5MR by gender.

Secondly, we identified the high-risk districts unlikely to meet the SDG targets for NMR and U5MR, even if the reduction in mortality rates that they saw between the last two rounds of NFHS (2005–2006 and 2015–2016) approximated their state’s overall reduction in mortality rates. These clusters of districts should be prioritized in order to prevent deaths among newborns and children under five. We also identified the best-practice districts, which are likely to meet the SDG target for both genders. The results of the present study can serve as critical input for policy-makers and health professionals to improve child survival and consequently achieve the SDG3 target of preventable deaths among children in India.

## Data and methods

### Ethics statement

The study is based on an anonymous publicly available dataset with no identifiable information on the survey participants; therefore, no ethics statement is required for this work.

### Data and sample design

We primarily used the most recent (fourth round) of the Demographic and Health Survey (DHS) for India, popularly known as the National Family Health Survey-4 (NFHS-4) conducted in 2015–2016 [1]. The previous three rounds of the NFHS were conducted in 1992–1993 (NFHS-1)[16], 1998–1999 (NFHS-2)[17], and 2005–2006 (NFHS-3)[2]. The NFHS surveys were conducted under the stewardship of the Ministry of Health and Family Welfare (MoHFW), Government of India. NFHS-4 was based on 1,315,617 children born of 699,686 women in 601,509 households with a response rate of 98%. The survey included 425,563 households from rural areas and 175,946 households from urban areas. The sample size for NFHS-4 was decided based on the need to produce indicators at district and state/union territory (UT). The sample was selected through a two-stage sample design: for the first stage, the Primary Sampling Units (PSU)s were villages in rural areas (selected with probability proportional to size); and Census Enumeration Blocks (CEB) for urban areas and in second stage; a random selection of 22 households in each PSU and each CEB were done for rural and urban area, respectively. The households were selected after a complete mapping and household listing operation was conducted in the selected first-stage units. If a selected PSU had at least 300 households, it was divided into segments of approximately 100–150 households. Two segments were then randomly selected for the survey using systematic sampling with probability proportional to segment size. Therefore, NFHS-4 cluster is thus either a PSU or a segment of a PSU [1].

The NFHS-4 report provides state- and national-level information on fertility (usually three years prior to the survey), family planning, infant and child morbidity and mortality (usually five years prior to the survey), maternal and reproductive health, nutritional status of women and children, and the quality of the health services. For the first time in the NFHS-4, district-level indicators were calculated for population and household profile, use of family planning methods, maternity care, child immunization rate, nutritional status of adults and children etc.

### Measures

We estimated the NMR and U5MR for Indian districts. We chose the reference period of these estimates as “ten years prior to the survey date” in order to maximize the size of sample needed to estimate those rates at district level. The NMR is defined as the probability (expressed as a rate per 1000 live births) of dying within the first month of life. Similarly, the U5MR is the probability (expressed as a rate per 1000 live births) of dying before reaching the age of five.

#### Demographic approach for computing NMR and U5MR

We applied direct methods of mortality-rate calculation using data on children’s date-of-birth, their survival status, and the date-of-death and age at death of deceased children. Our method is the same as that used to produce the mortality rate in DHS reports using the Stata package “*syncmrates*”. Rustein and Rojas describe the synthetic cohort probability approach using full birth histories data of women aged 15–49 to estimate the NMR and U5MR rate in detail [18]. A synthetic cohort life table approach is one in which mortality probabilities for small age-segments based on real cohort mortality experience are combined into the more common segments. This approach allows full use of the most recent data and is also time-period specific. We carried out district-level estimations of NMR and U5MR using the same synthetic cohort probability approach for total female and male separately, along with 95% confidence interval and level of significance. We carried out all the analysis in STATA S.E. 14.0 (STATA Corp., Inc., College Station, TX) version. Our inferences regarding NMR and U5MR are strictly based on the districts for which 1) estimated mortality rates are statistically significant at 10% level of significance; and 2) estimated mortality rates are greater than zero. Thus, while we provide all estimates in the appendices to this paper, we analyzed only significant estimates in the tables given in the text.

#### Categorization of districts

To present a state-wise comparative analysis of district status with reference to the SDG3 target on preventable deaths among new-borns and children, we categorized all districts as follows:

(a) District which had already achieved SDG3 (12 or fewer death per 1000 live births for NMR; 25 or fewer deaths per 1000 live births for U5MR in the study period)
(b) Districts which are on track to achieving, or will achieve, the target by 2030
(c) Districts which are lagging behind or unlikely to achieve the target by 2030

To include a district in category (b), we performed the following steps. First, we computed the state-specific yearly percentage reduction in NMR and U5MR between NFHS-3 (five years preceding the survey) and NFHS-4 (five years after the survey). We then applied the yearly state-specific reduction in the corresponding district’s mortality rate for a 20-year period. As our estimates based on NFHS-4 refer to the 10 years preceding the survey, the distance between the mid-point of this reference period (c. 2010) and the SDG target year (2030) is about 20 years. A district is assigned as being “on track” if, after experiencing state-specific reduction rate in 20 years, its expected mortality rate is less than the SDG3 target in 2030. Thus, we assume that reduction in mortality rate in a district during 2010–2030 will be similar to the reduction experienced by the corresponding state between the NFHS-3 and the NFHS-4. A district is assigned as being lagging behind (c) if it does not meet the criteria in either (a) or (b) discussed above.

## Results

### Disparity in NMR and U5MR across districts

We present district-level estimates of NMR (S1 Table and S1 Fig) and U5MR (S2 Table and S2 Fig) by gender for 10-year periods preceding the survey. Each estimated value is supported by a *p* value to indicate the statistical significance of the estimate. We also computed 95% uncertainty intervals for all indicators in view of the inherent uncertainty in child-mortality-related outcomes.

**Fig 1:**
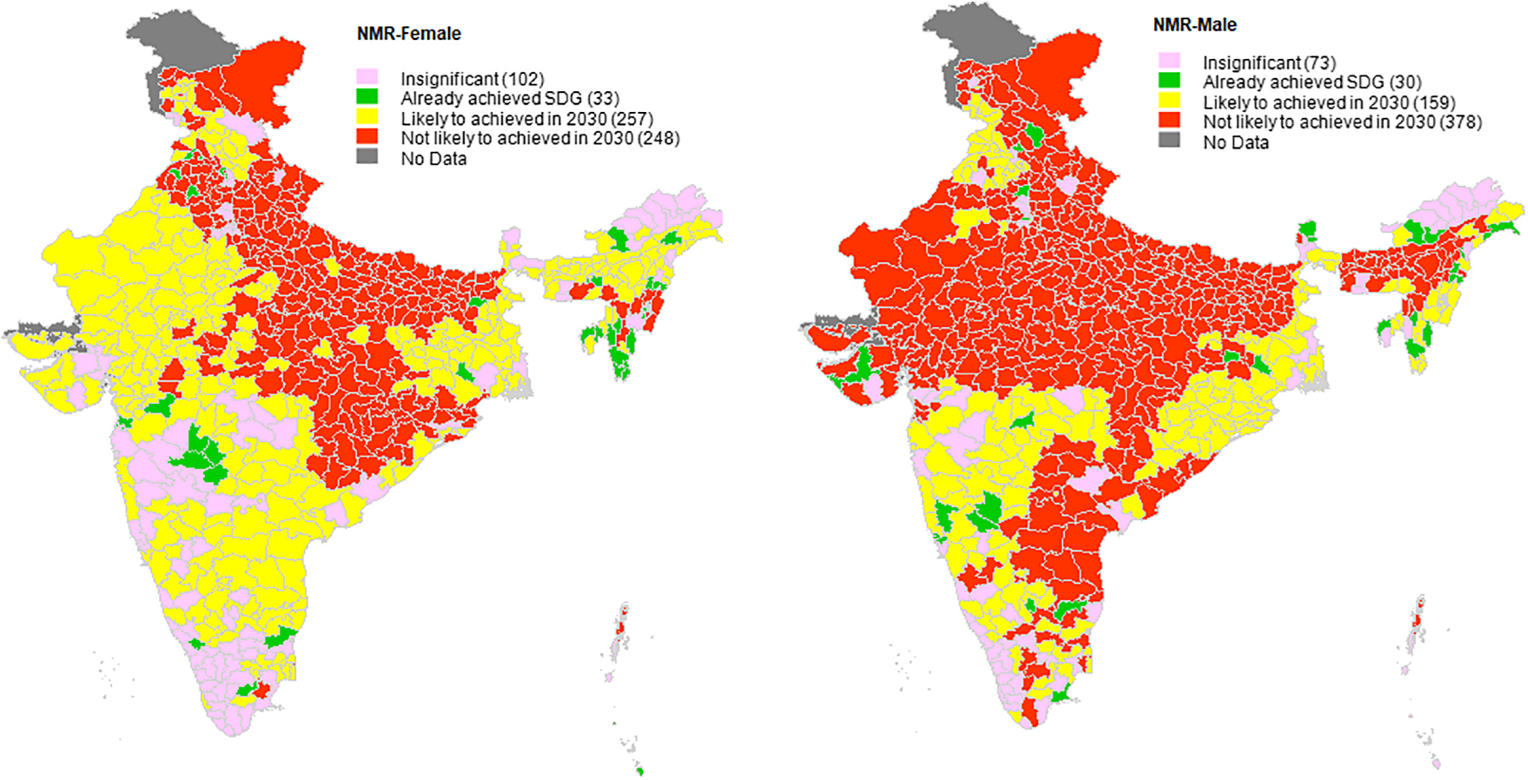
State-wise comparative analysis of neonatal mortality rate, India, 2005–2006 to 2015–2016 with reference to SDG3 target on preventable deaths among new-borns.

**Fig 2:**
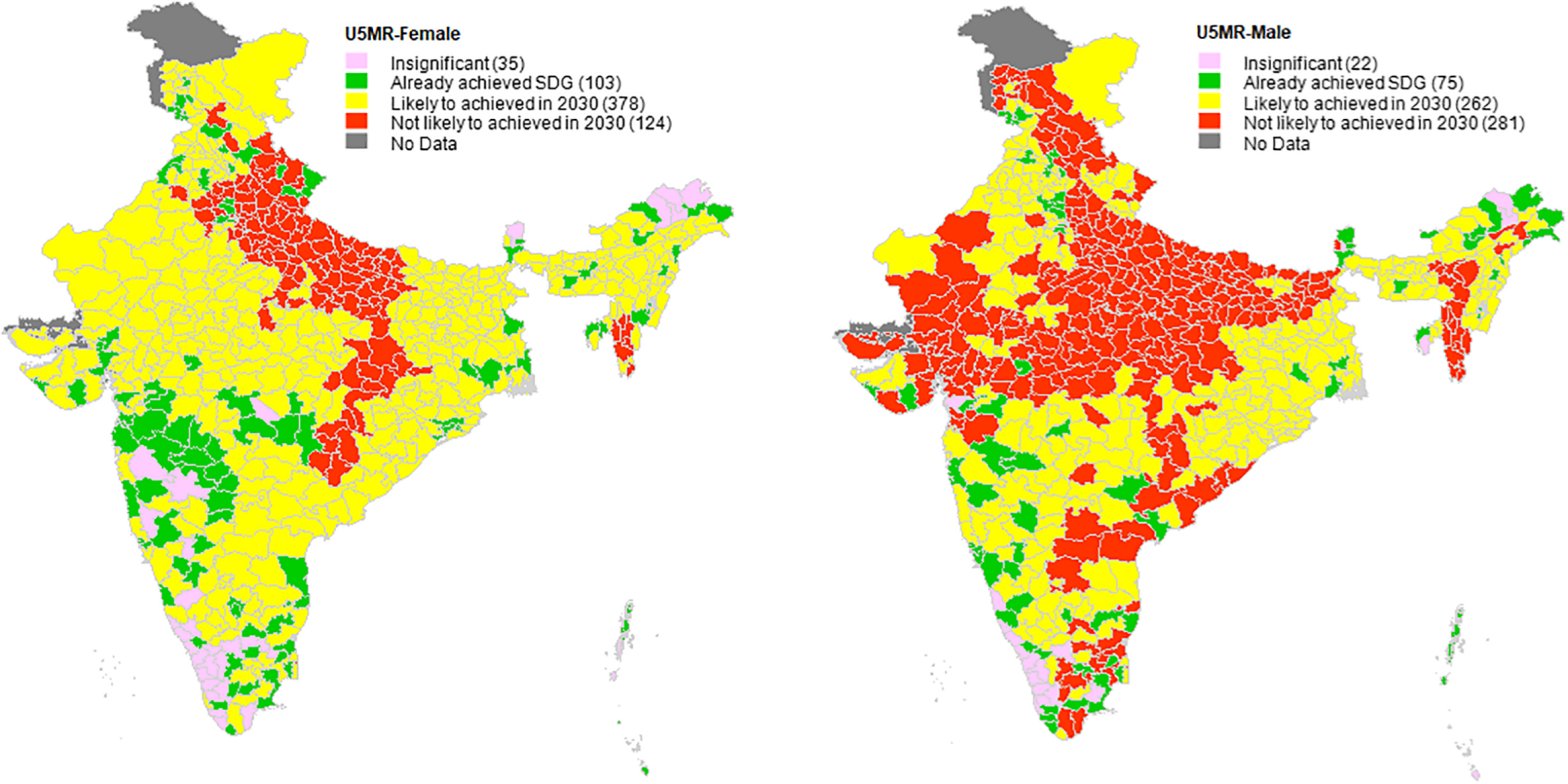
State-wise comparative analysis of under-five mortality rate, India, 2005–2006 and 2015–2016 with reference to SDG3 target on preventable deaths among children under the age of five.

Table 1 provides district level summary statistics of the estimated NMRs and U5MRs of those presented in Figs 1 and 2. One important inference from Table 1 is that the NFHS-4 sample size is large enough to estimate NMR by gender for the 10 years preceding the survey for more than 84% of the Indian districts (statistically significant at 10% level of significance). For then U5MR, the corresponding size is 94%. It is clear from Table 1 that all indicators are substantially higher than the SDG3 target for preventable deaths among children. On average, the estimated U5MR of India is double the targeted one (estimated 49.4 against targeted 25.0 deaths per 1000 live births in SDG3) whereas the estimated NMR is about 2.4 times greater than the targeted one (estimated 29.2 against targeted 12.0 deaths per 1000 live births in SDG3). The most striking thing about Table 1 is that while some districts have already reached a much lower level than that targeted in SDG3, other districts will need about five times the current reduction level to reach the SDG target by 2030.

**Table 1:** Descriptive statistics of district level NMR and U5MR by gender for 10-year periods preceding the survey, India, 2015–2016.

Variables	Number of districts with significant estimate	Mean	Standard Deviation (SD)	Minimum	Maximum	Coefficient of Variation (CV)
NMR Female	538	28.3	11.9	4.3	69.6	0.4195
NMR Male	567	34.2	15.0	4.5	97.7	0.4370
NMR Total	613	29.2	13.2	3.8	84.0	0.4538
U5MR Female	605	49.0	24.0	7.0	131.9	0.4899
U5MR Male	618	52.2	23.7	6.3	141.7	0.4538
U5MR Total	631	49.4	23.1	5.0	131.5	0.4674

For instance, the U5MR rate for male varies between 6.3 in South West district of national capital territory of Delhi to 141.7 in the tribal-dominated district Rayagada in Odisha, and so on. The coefficient of variations shows that the highest level of geographical disparity is observed in the U5MR among all mortality indicators. Finally, the female advantage in survival is clearer in the NMR (about 6 units per 1000 live births) than in the U5MR (about 3 units per 1000 live births).

### District status for SDG target on preventable deaths among new-borns and children under age 5: Achieved, on track, and lagging behind

Table 2 and Fig 1 present a state-wise comparative analysis of district-level NMR with reference to the SDG3 target on preventable deaths among new-borns. It is clear that while 9% (56 of 613 districts with significant estimates) already have an NMR as low as the SDG3 target during the study period, nearly half the districts (315) are not likely to achieve the SDG3 target on total NMR by 2030 if their pace of reduction remains similar to that between NFHS-3 and NFHS-4. NMR estimates by gender further reveal that 67% of the districts are not likely to achieve the SDG target for NMR for males, whereas only 46% of the districts are not likely to achieve the SDG3 target for NMR for females by 2030. For total NMR, most of the poorly performing districts are located in the states of Assam, Bihar, Chhattisgarh, Haryana, Madhya Pradesh, Uttar Pradesh, and Uttarakhand.

**Table 2:** State-wise comparative analysis of neonatal mortality rate, India, 2005–2006 and 2015–2016 with reference to SDG3 target on preventable deaths among new borns.

States	Total no of districts	NMR (per 1000 live births)											
		Female NMR				Male NMR				Total NMR			
		Number of districts with significant estimates	Already achieved SDG	Likely to be achieved in 2030	Not likely to be achieved in 2030	Number of districts with significant estimates	Already achieved SDG	Likely to be achieved in 2030	Not likely to be achieved in 2030	Number of districts with significant estimates	Already achieved SDG	Likely to be achieved in 2030	Not likely to be achieved in 2030
Andaman & Nicobar Islands	3	2	1	0	1	1	0	0	1	3	2	0	1
Andhra Pradesh	13	11	0	11	0	12	0	1	11	13	0	13	0
Arunachal Pradesh	16	7	1	6	0	7	3	4	0	12	7	5	0
Assam	27	27	1	25	1	27	0	1	26	27	0	8	19
Bihar	38	38	1	0	37	38	0	0	38	38	0	0	38
Chandigarh	1	1	0	0	1	0	0	0	0	1	0	1	0
Chhattisgarh	18	18	0	0	18	18	0	0	18	18	0	0	18
Dadra & Nagar Haveli	1	0	0	0	0	1	0	0	1	1	0	0	1
Daman & Diu	2	1	0	1	0	2	1	0	1	2	1	0	1
Delhi	9	5	0	1	4	4	1	3	0	7	3	4	0
Goa	2	1	0	1	0	1	1	0	0	2	1	0	1
Gujarat	26	23	1	22	0	23	2	0	21	26	2	7	17
Haryana	21	14	0	0	14	18	1	2	15	19	2	2	15
Himachal Pradesh	12	11	0	10	1	12	2	0	10	12	0	1	11
Jammu & Kashmir	22	21	0	11	10	21	0	6	15	22	0	8	14
Jharkhand	24	24	1	20	3	24	2	4	18	24	1	10	13
Karnataka	30	20	0	20	0	26	3	19	4	30	3	27	0
Kerala	14	2	1	1	0	2	0	2	0	3	2	1	0
Lakshadweep	1	1	0	1	0	1	1	0	0	1	0	1	0
Madhya Pradesh	50	50	0	22	28	50	0	0	50	50	0	1	49
Maharashtra	35	20	5	15	0	29	2	27	0	34	5	29	0
Manipur	9	8	3	0	5	9	0	9	0	9	1	4	4
Meghalaya	7	5	1	2	2	5	0	2	3	5	0	3	2
Mizoram	8	8	6	1	1	7	3	4	0	8	4	4	0
Nagaland	11	8	2	6	0	10	3	1	6	10	3	2	5
Odisha	30	29	0	5	24	30	0	30	0	30	0	28	2
Puducherry	4	2	1	0	1	3	0	0	3	4	3	0	1
Punjab	20	20	4	2	14	18	0	16	2	20	0	13	7
Rajasthan	33	33	0	33	0	33	0	1	32	33	0	21	12
Sikkim	4	2	0	2	0	3	2	0	1	4	3	0	1
Tamil Nadu	32	15	2	12	1	23	2	10	11	29	9	20	0
Telangana	10	9	0	9	0	9	0	1	8	10	0	10	0
Tripura	4	4	2	2	0	3	1	2	0	4	2	2	0
Uttar Pradesh	71	71	0	1	70	71	0	0	71	71	0	0	71
Uttarakhand	13	12	0	0	12	12	0	0	12	13	1	0	12
West Bengal	19	15	0	15	0	14	0	14	0	18	1	17	0
**Total**	**640**	**538**	**33(6%)**	**257(48%)**	**248(46%)**	**567**	**30(5%)**	**159(28%)**	**378(67%)**	**613**	**56(9%)**	**242(39%)**	**315(52%)**

Moreover, a clear geographical clustering of districts is seen in the female NMR (Fig 1). Most of the districts unlikely to achieve the female NMR target are clustered in north-central and eastern belt of the country. On the other hand, for male NMR, we observe three geographical clustering of districts, namely, north-central-west, north-east, and south-east, which are unlikely to meet the target by 2030. Thus, we observe quite a few high-risk districts for male NMR in rich and advanced states such as Andhra Pradesh, Gujarat, Haryana, Telangana etc.

Table 3 and Fig 2 presents a state-wise comparative analysis of district level U5MR with reference to the SDG3 target on preventable deaths among children under the age of five. In Indian districts the U5MR status in terms of reaching the SDG3 is much better than the NMR status. Less than one-third of the districts (177) of India are unlikely to achieve the SDG3 on the U5MR by 2030. We also observed a clear female advantage in survivorship in district level U5M, with 20% and 45% of the districts probably not achieving the SDG target for female and males, respectively. Poorly performing districts in terms of male U5MR are concentrated in north-central and west India, with a few clusters in the southeast and northeast. At state level, there are two particular states, namely, Chhattisgarh and Uttar Pradesh, where 97% of districts are unlikely to meet the SDG targets for both NMR and U5MR irrespective of gender (Tables 2 and 3).

**Table 3:** State-wise comparative analysis of under-five mortality rate, India, 2005–2006 and 2015–2016 with reference to SDG3 target on preventable deaths among children aged under five.

States	Total no of districts	U5MR (per 1000 live births)											
		Female U5MR				Male U5MR				Total U5MR			
		Number of districts with significant estimates	Already achieved SDG	Likely to be achieved in 2030	Not likely to be achieved in 2030	Number of districts with significant estimates	Already achieved SDG	Likely to be achieved in 2030	Not likely to be achieved in 2030	Number of districts with significant estimates	Already achieved SDG	Likely to be achieved in 2030	Not likely to be achieved in 2030
Andaman & Nicobar Islands	3	2	2	0	0	2	2	0	0	3	3	0	0
Andhra Pradesh	13	13	1	12	0	13	1	4	8	13	1	12	0
Arunachal Pradesh	16	12	4	8	0	14	7	7	0	15	6	9	0
Assam	27	27	1	26	0	27	0	16	11	27	0	22	5
Bihar	38	38	0	38	0	38	0	3	35	38	0	28	10
Chandigarh	1	1	0	1	0	1	0	1	0	1	0	1	0
Chhattisgarh	18	18	0	3	15	18	0	6	12	18	0	3	15
Dadra & Nagar Haveli	1	1	1	0	0	1	0	1	0	1	0	1	0
Daman & Diu	2	2	2	0	0	2	1	0	1	2	1	1	0
Delhi	9	8	2	0	6	8	3	4	1	9	4	0	5
Goa	2	2	1	1	0	2	2	0	0	2	2	0	0
Gujarat	26	26	6	20	0	25	3	2	20	26	3	17	6
Haryana	21	21	4	4	13	21	6	13	2	21	4	11	6
Himachal Pradesh	12	12	3	6	3	12	1	0	11	12	1	3	8
Jammu & Kashmir	22	22	6	16	0	22	3	8	11	22	4	15	3
Jharkhand	24	24	1	22	1	24	1	23	0	24	1	23	0
Karnataka	30	27	10	17	0	29	7	22	0	30	8	22	0
Kerala	14	3	2	1	0	4	4	0	0	7	7	0	0
Lakshadweep	1	1	0	1	0	1	1	0	0	1	0	1	0
Madhya Pradesh	50	50	1	49	0	50	1	2	47	50	1	20	29
Maharashtra	35	31	18	13	0	35	7	26	2	35	10	25	0
Manipur	9	9	4	5	0	9	2	7	0	9	2	7	0
Meghalaya	7	7	1	6	0	7	1	6	0	7	1	6	0
Mizoram	8	8	0	2	6	8	0	0	8	8	0	1	7
Nagaland	11	11	2	9	0	11	2	9	0	11	3	8	0
Odisha	30	30	2	28	0	30	0	30	0	30	1	29	0
Puducherry	4	3	2	0	1	4	1	0	3	4	2	0	2
Punjab	20	20	3	17	0	20	3	17	0	20	1	19	0
Rajasthan	33	33	0	33	0	33	0	16	17	33	0	33	0
Sikkim	4	2	1	1	0	3	2	0	1	4	3	0	1
Tamil Nadu	32	25	13	12	0	29	9	7	13	31	15	16	0
Telangana	10	10	0	10	0	10	1	6	3	10	1	9	0
Tripura	4	4	2	2	0	3	1	2	0	4	2	2	0
Uttar Pradesh	71	71	0	2	69	71	0	0	71	71	0	0	71
Uttarakhand	13	13	3	0	10	13	0	9	4	13	0	4	9
West Bengal	19	18	5	13	0	18	3	15	0	19	4	15	0
**Total**	**640**	**605**	**103(17%)**	**378(62%)**	**124(20%)**	**618**	**75(12%)**	**262(42%)**	**281(45%)**	**631**	**91(14%)**	**363(58%)**	**177(28%)**

## Discussion and conclusion

We know of no other study that estimates district-level NMR and U5MR in India using the most recent full birth history information from the Indian DHS. Our estimates refer to the period 2005–2006 to 2015–2016, a period when the Government of India launched an ambitious National Rural Health Mission (later renamed as the National Health Mission or NHM) to address factors contributing to neonatal and under-five mortality. The novelty of this study also lies in its ability to identify districts with both a high and a low propensity to meet the SDG3 target on preventable deaths among new-borns and children under the age of five.

In line with previous research findings, our analysis again demonstrates the enormous variation across the districts of India in NMR and U5MR [13]. While about 9% and 14% of Indian districts have already achieved the SDG3 target for NMR and U5MR, respectively, in the study period, a great many districts may not achieve the SDG3 target even in 2030. The picture for U5MR, particularly for females, was optimistic. Our study used a state-level comparative analysis to show that the majority of high-risk districts for NMR and U5MR belong to the states of Assam, Bihar, Chhattisgarh, Jharkhand, Madhya Pradesh, Orissa, Rajasthan, and Uttar Pradesh. Yet, the high-risk districts in NMR terms are not limited to poorer states but spread across rich and advanced states like Andhra Pradesh, Gujarat, and Telangana, particularly for male neonates. The enormous variation in NMR and U5MR among Indian districts are due two reasons. First, there exists wide disparities in the level of socio-economic development in the districts within and between different states of India[19]. For instance, census 2011 data shows that district level female literacy rate varies between 24.25 to 88.62 percent; district level urbanization varies between 0 to 100 percent and that of safe drinking water ranges between 8.60 to 99.60 percent [20]. Secondly, the district level disparities in NMR and U5MR also links to implementations of interventions. Despite governments efforts to improve the infrastructure of backwards districts through National Rural Health Mission, the Annual Health Survey and District Level Household and Facility Survey -4 data shows presence of enormous variation in accessibility and availability of health infrastructure, human resources and services in the health care system. Our findings on the districts’ status with reference to SDG3 do not coincide exactly with the results of an earlier study [13] which evaluated the district’s status with respect to (MDG4) because of the targets set by MDG4 and SDG3 are different. Unlike the previous study, ours analyzed the situation by gender.

Secondly, similar to previous studies [4,14,15], we also observed a clear geographical clustering of the high-risk districts for NMR and U5MR. While there are two relatively smaller clusters of high-risk districts for female U5MR, we observed a large cluster (124 districts for female U5MR) of districts in the north, east, and west regions of India, followed another four clusters in northeast and south India. We also observed clear geographical pattern in NMRs across districts.

Finally, our analysis shows that female NMR is lower than male, a much-expected finding due to female’s biological advantage observed in other countries of the world. However, this advantage reduces once we move toward U5MR. This indicates potential discrimination against girl children in behavioral factors such as nutrition and healthcare in their early years of life, a factor that has been discussed in previous studies [21–26] on developing countries.

The findings of this study have great relevance for policy-makers, health professionals, program managers and administrative authorities. Earlier studies have shown that the reduction in U5MR in India was stalled in early 2000 [27,28]. However, the inauguration of the National Rural Health Mission in the mid-2000s led to a sharp reduction in the infant mortality rate (IMR) and U5MR during 2005–2015 [1]. Yet, as our study shows, the extent of this reduction may not be enough to achieve the SDG3 goals for the NMR and U5MR by 2030. Secondly, India needs more intensive programs, particularly in certain geographical pockets—not just in the most populous and demographically backward regions, but also in some zones in the rich and advanced states. At state level, Chhattisgarh and Uttar Pradesh deserve particular attention, as the majority of districts in these two states may not achieve the SDG3 target by 2030 if they continue a decline similar to that experienced during NFHS-3 and NFHS-4. Finally, more investment is needed to improve the survival rate of neonates. During the study period, under the NHM program, the government had already aimed to improve child health through a number of program such as Integrated Management of Neonatal and Childhood Illnesses (IMNCI); Navjaat Shishu Suraksha Karyakram; home-based care of newborns, universal immunization through mother and child tracking system, early detection and appropriate management of Acute Respiratory Infections (ARI), diarrhea and other infectious diseases etc.[29]. However, the main barrier to extensive coverage of integrated packages for the health of mothers, neonates, and children in countries like India [30] is inadequate operational management, especially at the district level [31]. The government should thus focus more intensively on how these programs are executed in the low performing districts. The operational and monitoring execution at district level should be in two folds; first, at individual level-the district administrators, NGO’s etc. should give emphasis on awareness of the district level intervention program through community based awareness program and educate parents for the possible high risk factors and preventive measures of child health. Also, focus should be given on level of education to the girls and mothers especially for poor, rural and backward community. Secondly, State govt. should give priority for the improvement of public health facilities through reinforced in institutional deliveries, fill the eligible and trained human resources, make available of adequate functioning testing machine and infrastructures at Sub-Centre (SC), Primary Health Centre (PHC), Community Health Centres (CHCs) and District Hospitals(DH). At the same time, District administration should efficiently monitor the intervention program so that it could reach to the needy people. It is expected that low performing districts with high mortality would response well in mortality reduction if above strategies are adopted. As earlier literature highlighted socioeconomic inequalities in access to child healthcare [30, 32–35] any program should also intensify its focus where more attention is needed.

## Limitations

The study has a few limitations. First, in this analysis we assumed that reduction in NMR or U5MR in the districts from the study period to 2030 would be same as the reduction experienced by corresponding states during NFHS-3 and NFHS-4. As noted earlier, there is an enormous variation between the districts; hence, the pace of reduction in the child mortality rate may also vary substantially across the districts within a particular state of India. Particularly, the rate of reduction in mortality may be higher in the districts with high level of mortality, and vice versa. There is, however, no other alternative, in the absence of district-level estimates from the previous rounds of the NFHS.

Secondly, it should be noted that the reduction in under-five deaths during the NFHS-3 and NFHS-4 were the highest among all rounds of NFHS. Thus, when we assume that the future reduction in NMR and U5MR will be same as that which occurred between the NFHS-3 and NFHS-4, we are providing an optimistic picture.

## Supporting information

S1 Fig Map of neonatal mortality rate of all Indian districts by gender, India, 2015–2016. (TIF)

S2 Fig Map of under-five mortality rate of all Indian districts by gender, India, 2015–2016. (TIF)

S1 Table Estimated districtwise neonatal mortality rate for ten-years periods preceding the survey, by gender, India, 2015–16. (PDF)

S2 Table Estimated districtwise under-five mortality rate for ten-years periods preceding the survey, by gender, India, 2015–16. (PDF)
