# Supplementary figures and images for: Neonatal and under-five mortality rate in Indian districts with reference to Sustainable Development Goal 3: An analysis of the National Family Health Survey of India (NFHS), 2015–2016
Source: PLoS One. 2018 Jul 30;13(7):e0201125. doi: 10.1371/journal.pone.0201125 (PMC6066210; doi:10.1371/journal.pone.0201125)

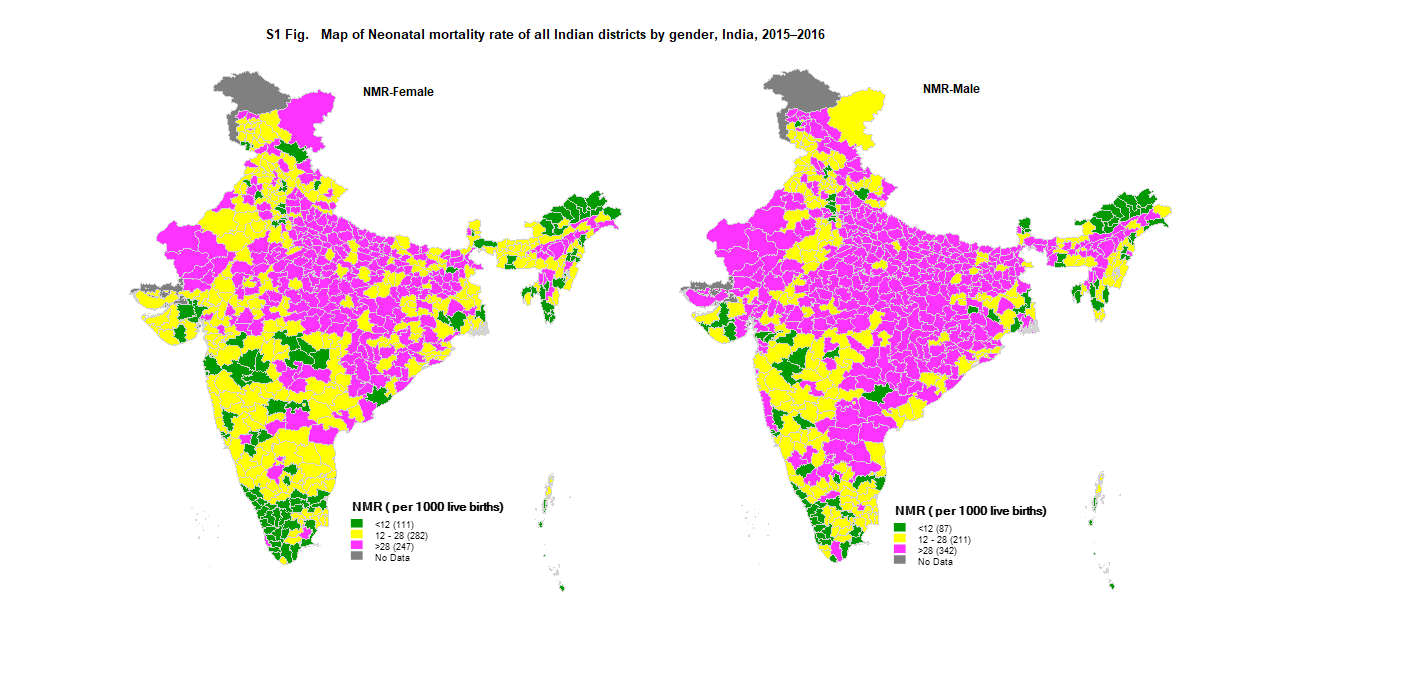

Supplement: S1 Fig — (TIF) [file pone.0201125.s001.tif]

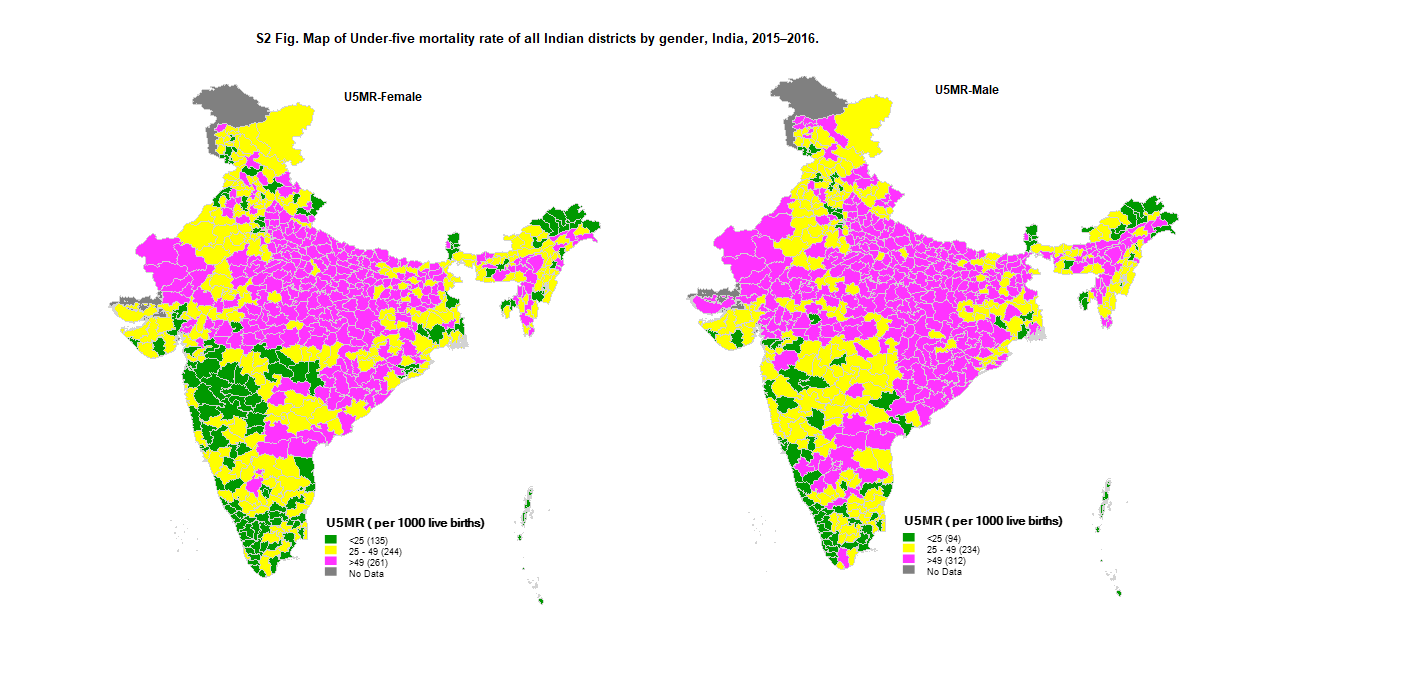

Supplement: S2 Fig — (TIF) [file pone.0201125.s002.tif]
