# Supplementary material for: Neonatal and under-five mortality rate in Indian districts with reference to Sustainable Development Goal 3: An analysis of the National Family Health Survey of India (NFHS), 2015–2016
Source: PLoS One. 2018 Jul 30;13(7):e0201125. doi: 10.1371/journal.pone.0201125 (PMC6066210; doi:10.1371/journal.pone.0201125)
Supplement: S1 Table — (PDF) [file pone.0201125.s003.pdf]

**S1 Table. Estimated Districtwise Neonatal mortality rate for ten-years periods preceding the survey, by gender, India, 2015-16.**

| District | Female | | | | Male | | | | Total | | | |
|---|---|---|---|---|---|---|---|---|---|---|---|---|
| | NMR | P value | 95% CI | | NMR | P value | 95% CI | | NMR | P value | 95% CI | |
| | | | Lower | Upper | | | Lower | Upper | | | Lower | Upper |
| Kupwara | 33.2 | 0.000 | 18.1 | 48.3 | 58.7 | 0.000 | 35.7 | 81.8 | 46.5 | 0.000 | 31.9 | 61.1 |
| Badgam | 18.7 | 0.006 | 5.3 | 32.1 | 5.8 | 0.259 | -4.3 | 16.0 | 12.2 | 0.002 | 4.4 | 20.0 |
| Leh | 33.7 | 0.046 | 0.5 | 66.9 | 23.8 | 0.016 | 4.5 | 43.1 | 28.5 | 0.000 | 12.6 | 44.3 |
| Kargil | 25.2 | 0.036 | 1.7 | 48.7 | 39.4 | 0.000 | 18.5 | 60.4 | 32.8 | 0.000 | 16.8 | 48.9 |
| Punch | 20.4 | 0.000 | 9.1 | 31.7 | 24.8 | 0.000 | 12.0 | 37.6 | 22.8 | 0.000 | 12.1 | 33.5 |
| Rajouri | 23.1 | 0.001 | 9.1 | 37.1 | 23.2 | 0.001 | 9.5 | 36.8 | 23.1 | 0.000 | 12.3 | 34.0 |
| Kathua | 31.8 | 0.001 | 12.2 | 51.3 | 20.7 | 0.009 | 5.1 | 36.4 | 25.8 | 0.000 | 14.5 | 37.2 |
| Baramula | 21.7 | 0.009 | 5.5 | 38.0 | 29.7 | 0.003 | 10.0 | 49.4 | 25.9 | 0.000 | 14.9 | 36.9 |
| Bandipore | 30.7 | 0.003 | 10.4 | 51.0 | 40.8 | 0.000 | 22.7 | 58.9 | 35.6 | 0.000 | 20.6 | 50.5 |
| Srinagar | 15.7 | 0.034 | 1.2 | 30.1 | 23.5 | 0.095 | -4.1 | 51.1 | 19.8 | 0.002 | 7.3 | 32.2 |
| Ganderbal | 15.3 | 0.008 | 3.9 | 26.7 | 41.4 | 0.001 | 16.6 | 66.2 | 27.7 | 0.000 | 13.5 | 42.0 |
| Pulwama | 13.8 | 0.038 | 0.7 | 26.8 | 43.1 | 0.000 | 19.8 | 66.3 | 28.8 | 0.000 | 15.1 | 42.5 |
| Shupiyan | 29.8 | 0.005 | 8.9 | 50.6 | 20.8 | 0.004 | 6.7 | 34.8 | 25.4 | 0.000 | 13.0 | 37.7 |
| Anantnag | 20.4 | 0.010 | 4.8 | 36.0 | 31.0 | 0.000 | 14.5 | 47.5 | 26.2 | 0.000 | 13.9 | 38.5 |
| Kulgam | 20.4 | 0.019 | 3.3 | 37.5 | 44.1 | 0.000 | 26.3 | 61.8 | 32.8 | 0.000 | 18.2 | 47.4 |
| Doda | 14.2 | 0.014 | 2.9 | 25.5 | 27.4 | 0.003 | 9.6 | 45.3 | 20.8 | 0.000 | 12.8 | 28.8 |
| Ramban | 13.6 | 0.009 | 3.3 | 23.9 | 25.7 | 0.000 | 12.8 | 38.7 | 19.9 | 0.000 | 11.7 | 28.2 |
| Kishtwar | 26.7 | 0.002 | 10.1 | 43.3 | 34.6 | 0.000 | 18.1 | 51.0 | 30.8 | 0.000 | 19.0 | 42.6 |
| Udhampur | 22.7 | 0.005 | 6.9 | 38.5 | 19.0 | 0.003 | 6.4 | 31.5 | 20.8 | 0.000 | 10.6 | 31.1 |
| Reasi | 13.2 | 0.015 | 2.5 | 23.9 | 21.3 | 0.000 | 10.8 | 31.8 | 17.4 | 0.000 | 9.6 | 25.2 |
| Jammu | 8.0 | 0.203 | -4.3 | 20.4 | 19.4 | 0.036 | 1.3 | 37.6 | 14.2 | 0.005 | 4.2 | 24.3 |
| Samba | 12.3 | 0.060 | -0.5 | 25.1 | 12.4 | 0.050 | 0.0 | 24.8 | 12.4 | 0.010 | 3.0 | 21.7 |
| Chamba | 31.2 | 0.001 | 12.9 | 49.5 | 48.7 | 0.000 | 31.5 | 65.9 | 40.2 | 0.000 | 21.8 | 58.6 |
| Kangra | 14.3 | 0.062 | -0.7 | 29.2 | 27.1 | 0.018 | 4.7 | 49.5 | 21.3 | 0.000 | 9.8 | 32.7 |
| Lahul and spiti | 10.8 | 0.154 | -4.1 | 25.7 | 31.1 | 0.010 | 7.4 | 54.8 | 21.0 | 0.010 | 5.0 | 37.1 |
| Kullu | 19.8 | 0.041 | 0.8 | 38.7 | 11.9 | 0.072 | -1.1 | 24.9 | 15.8 | 0.009 | 3.9 | 27.7 |
| Mandi | 33.7 | 0.001 | 13.4 | 54.0 | 18.0 | 0.077 | -2.0 | 38.0 | 25.9 | 0.000 | 12.5 | 39.3 |
| Hamirpur | 21.0 | 0.018 | 3.6 | 38.5 | 28.0 | 0.006 | 7.8 | 48.1 | 24.6 | 0.001 | 10.3 | 38.8 |
| Una | 17.4 | 0.054 | -0.3 | 35.2 | 34.7 | 0.001 | 13.8 | 55.6 | 26.5 | 0.000 | 12.6 | 40.4 |
| Bilaspur | 15.5 | 0.037 | 0.9 | 30.1 | 10.5 | 0.089 | -1.6 | 22.6 | 13.0 | 0.003 | 4.3 | 21.6 |
| Solan | 21.6 | 0.009 | 5.4 | 37.8 | 26.4 | 0.008 | 7.0 | 45.8 | 24.2 | 0.000 | 11.2 | 37.1 |
| Sirmaur | 19.8 | 0.015 | 3.8 | 35.8 | 34.8 | 0.001 | 13.4 | 56.2 | 27.3 | 0.000 | 15.7 | 38.9 |
| Shimla | 17.5 | 0.044 | 0.4 | 34.5 | 42.0 | 0.010 | 9.9 | 74.0 | 29.7 | 0.000 | 14.5 | 44.8 |
| Kinnaur | 55.3 | 0.004 | 17.4 | 93.3 | 48.0 | 0.002 | 17.2 | 78.8 | 51.6 | 0.000 | 29.4 | 73.7 |
| Gurdaspur | 12.9 | 0.047 | 0.2 | 25.6 | 22.5 | 0.013 | 4.7 | 40.2 | 18.0 | 0.000 | 8.6 | 27.4 |
| Kapurthala | 11.6 | 0.050 | 0.0 | 23.3 | 20.9 | 0.033 | 1.7 | 40.1 | 16.4 | 0.012 | 3.5 | 29.2 |
| Jalandhar | 24.5 | 0.011 | 5.6 | 43.3 | 13.1 | 0.026 | 1.6 | 24.6 | 18.1 | 0.019 | 3.0 | 33.1 |
| Hoshiarpur | 15.8 | 0.047 | 0.2 | 31.4 | 28.6 | 0.002 | 10.5 | 46.7 | 22.4 | 0.000 | 10.8 | 34.0 |
| Sangrur | 16.5 | 0.053 | -0.2 | 33.2 | 36.6 | 0.000 | 16.6 | 56.5 | 26.5 | 0.000 | 12.8 | 40.3 |
| Fatehgarh sahib | 29.6 | 0.030 | 2.8 | 56.5 | 18.3 | 0.022 | 2.6 | 33.9 | 23.6 | 0.001 | 9.6 | 37.7 |
| Ludhiana | 22.4 | 0.017 | 3.9 | 40.8 | 18.9 | 0.005 | 5.7 | 32.2 | 20.5 | 0.001 | 8.4 | 32.6 |
| Moga | 47.5 | 0.000 | 24.9 | 70.1 | 37.0 | 0.000 | 18.5 | 55.4 | 42.0 | 0.000 | 27.0 | 57.0 |
| Firozpur | 17.6 | 0.023 | 2.4 | 32.7 | 24.5 | 0.002 | 9.3 | 39.8 | 21.2 | 0.000 | 9.6 | 32.8 |
| Muktsar | 16.9 | 0.025 | 2.1 | 31.7 | 27.4 | 0.000 | 12.5 | 42.3 | 23.1 | 0.000 | 13.7 | 32.5 |
| Faridkot | 11.3 | 0.040 | 0.5 | 22.0 | 14.5 | 0.009 | 3.6 | 25.3 | 13.1 | 0.001 | 5.3 | 21.0 |
| Bathinda | 37.9 | 0.005 | 11.2 | 64.6 | 13.6 | 0.118 | -3.5 | 30.7 | 25.2 | 0.000 | 12.9 | 37.6 |
| Mansa | 9.8 | 0.061 | -0.4 | 20.1 | 27.1 | 0.004 | 8.8 | 45.4 | 18.9 | 0.003 | 6.5 | 31.4 |
| Patiala | 17.0 | 0.014 | 3.4 | 30.6 | 14.5 | 0.000 | 6.4 | 22.5 | 15.6 | 0.004 | 4.9 | 26.3 |
| Amritsar | 29.7 | 0.002 | 10.6 | 48.8 | 16.2 | 0.016 | 3.0 | 29.4 | 22.2 | 0.000 | 11.1 | 33.3 |
| Tarn taran | 14.5 | 0.023 | 2.0 | 27.0 | 18.7 | 0.007 | 5.0 | 32.4 | 16.7 | 0.011 | 3.8 | 29.6 |
| Rupnagar | 43.2 | 0.000 | 19.2 | 67.1 | 9.9 | 0.125 | -2.7 | 22.5 | 25.4 | 0.001 | 11.1 | 39.8 |
| Sahibzada ajit singh nagar | 11.9 | 0.045 | 0.2 | 23.5 | 18.3 | 0.026 | 2.1 | 34.4 | 15.4 | 0.004 | 5.0 | 25.8 |
| Shahid bhagat singh nagar | 27.9 | 0.008 | 7.1 | 48.7 | 32.2 | 0.001 | 13.0 | 51.4 | 30.2 | 0.001 | 12.4 | 48.0 |
| Barnala | 31.2 | 0.006 | 8.9 | 53.5 | 51.0 | 0.002 | 19.0 | 83.1 | 41.8 | 0.000 | 20.9 | 62.7 |
| Chandigarh | 30.6 | 0.003 | 10.7 | 50.5 | 13.0 | 0.129 | -3.8 | 29.7 | 21.8 | 0.002 | 7.9 | 35.7 |
| Uttarkashi | 32.4 | 0.000 | 14.9 | 50.0 | 38.7 | 0.000 | 18.3 | 59.1 | 35.8 | 0.000 | 22.7 | 48.9 |
| Chamoli | 27.9 | 0.003 | 9.3 | 46.5 | 17.1 | 0.007 | 4.6 | 29.6 | 22.4 | 0.001 | 9.6 | 35.2 |
| Rudraprayag | 8.5 | 0.231 | -5.4 | 22.5 | 18.3 | 0.025 | 2.3 | 34.2 | 13.5 | 0.005 | 4.1 | 22.9 |
| Tehri garhwal | 12.3 | 0.004 | 3.9 | 20.7 | 16.1 | 0.004 | 5.0 | 27.3 | 14.3 | 0.001 | 5.7 | 22.9 |
| Dehradun | 18.0 | 0.004 | 5.8 | 30.2 | 18.0 | 0.000 | 7.9 | 28.1 | 18.0 | 0.000 | 12.1 | 23.8 |
| Garhwal | 12.6 | 0.085 | -1.7 | 26.9 | 9.7 | 0.177 | -4.4 | 23.7 | 11.0 | 0.011 | 2.5 | 19.6 |
| Pithoragarh | 15.7 | 0.013 | 3.3 | 28.1 | 39.6 | 0.001 | 16.1 | 63.0 | 28.3 | 0.000 | 15.0 | 41.6 |
| Bageshwar | 38.3 | 0.000 | 17.3 | 59.2 | 25.4 | 0.002 | 9.7 | 41.1 | 31.5 | 0.000 | 19.7 | 43.2 |
| Almora | 20.3 | 0.003 | 6.9 | 33.7 | 49.5 | 0.001 | 20.4 | 78.6 | 35.2 | 0.000 | 21.9 | 48.6 |
| Champawat | 41.6 | 0.000 | 20.8 | 62.5 | 26.5 | 0.001 | 10.7 | 42.3 | 33.9 | 0.000 | 21.5 | 46.2 |
| Nainital | 36.9 | 0.000 | 23.5 | 50.2 | 24.3 | 0.000 | 12.6 | 36.1 | 30.3 | 0.000 | 21.8 | 38.8 |
| Udham singh nagar | 26.3 | 0.000 | 14.6 | 38.1 | 34.3 | 0.000 | 22.0 | 46.5 | 30.5 | 0.000 | 22.3 | 38.7 |
| Hardwar | 39.3 | 0.000 | 27.4 | 51.3 | 39.4 | 0.000 | 30.9 | 47.9 | 39.4 | 0.000 | 30.8 | 47.9 |
| Panchkula | 7.9 | 0.168 | -3.3 | 19.0 | 12.4 | 0.026 | 1.5 | 23.3 | 10.5 | 0.017 | 1.9 | 19.1 |
| Ambala | 6.8 | 0.218 | -4.0 | 17.6 | 18.3 | 0.009 | 4.6 | 32.0 | 13.6 | 0.002 | 4.8 | 22.4 |
| Yamunanagar | 17.0 | 0.089 | -2.6 | 36.5 | 17.8 | 0.015 | 3.4 | 32.1 | 17.4 | 0.001 | 6.9 | 28.0 |
| Kurukshetra | 24.6 | 0.022 | 3.6 | 45.6 | 16.9 | 0.012 | 3.7 | 30.2 | 20.2 | 0.002 | 7.2 | 33.2 |
| Kaithal | 27.0 | 0.004 | 8.6 | 45.4 | 34.8 | 0.000 | 15.5 | 54.1 | 31.2 | 0.000 | 19.0 | 43.4 |
| Karnal | 18.6 | 0.007 | 5.1 | 32.1 | 10.2 | 0.083 | -1.3 | 21.7 | 14.1 | 0.001 | 5.9 | 22.2 |
| Panipat | 4.1 | 0.168 | -1.7 | 9.8 | 2.4 | 0.299 | -2.1 | 7.0 | 3.2 | 0.109 | -0.7 | 7.1 |
| Sonipat | 7.9 | 0.127 | -2.2 | 18.0 | 5.1 | 0.157 | -2.0 | 12.3 | 6.4 | 0.036 | 0.4 | 12.3 |

| | | | | | | | | | | | | |
|---|---|---|---|---|---|---|---|---|---|---|---|---|
| Jind | 21.9 | 0.001 | 9.2 | 34.6 | 15.6 | 0.036 | 1.1 | 30.1 | 18.7 | 0.000 | 9.8 | 27.6 |
| Fatehabad | 23.2 | 0.004 | 7.3 | 39.2 | 20.1 | 0.003 | 6.9 | 33.2 | 21.6 | 0.000 | 9.6 | 33.6 |
| Sirsa | 32.7 | 0.000 | 15.2 | 50.1 | 31.9 | 0.000 | 15.7 | 48.1 | 32.3 | 0.000 | 20.8 | 43.7 |
| Hisar | 33.9 | 0.005 | 10.5 | 57.3 | 15.5 | 0.019 | 2.6 | 28.4 | 23.7 | 0.000 | 12.1 | 35.3 |
| Bhiwani | 30.6 | 0.000 | 13.5 | 47.8 | 45.5 | 0.000 | 21.1 | 69.8 | 38.5 | 0.000 | 25.1 | 51.8 |
| Rohtak | 18.7 | 0.041 | 0.8 | 36.7 | 46.0 | 0.000 | 24.9 | 67.1 | 33.4 | 0.000 | 20.4 | 46.4 |
| Jhajjar | 23.9 | 0.013 | 5.1 | 42.6 | 31.2 | 0.001 | 12.3 | 50.0 | 28.2 | 0.000 | 14.1 | 42.2 |
| Mahendragarh | 14.8 | 0.057 | -0.5 | 30.1 | 12.1 | 0.014 | 2.4 | 21.8 | 13.2 | 0.003 | 4.4 | 22.0 |
| Rewari | 6.7 | 0.158 | -2.6 | 16.1 | 20.2 | 0.003 | 6.9 | 33.4 | 14.7 | 0.001 | 6.4 | 22.9 |
| Gurgaon | 1.3 | 0.204 | -0.7 | 3.2 | 5.5 | 0.237 | -3.6 | 14.7 | 3.6 | 0.195 | -1.8 | 8.9 |
| Mewat | 43.9 | 0.000 | 29.4 | 58.3 | 40.5 | 0.000 | 24.9 | 56.2 | 42.1 | 0.000 | 33.9 | 50.4 |
| Faridabad | 20.2 | 0.128 | -5.8 | 46.2 | 23.2 | 0.003 | 8.1 | 38.2 | 21.9 | 0.000 | 10.5 | 33.2 |
| Palwal | 22.2 | 0.002 | 8.4 | 36.0 | 18.9 | 0.005 | 5.6 | 32.2 | 20.4 | 0.000 | 11.8 | 29.0 |
| North west | 26.5 | 0.036 | 1.8 | 51.2 | 9.1 | 0.111 | -2.1 | 20.3 | 16.7 | 0.012 | 3.6 | 29.9 |
| North | 27.5 | 0.022 | 4.0 | 51.0 | 22.7 | 0.090 | -3.6 | 49.0 | 25.1 | 0.006 | 7.3 | 42.9 |
| North east | 20.1 | 0.033 | 1.7 | 38.5 | 32.3 | 0.003 | 11.2 | 53.5 | 26.9 | 0.000 | 12.9 | 40.8 |
| East | 3.9 | 0.297 | -3.4 | 11.3 | 15.1 | 0.110 | -3.4 | 33.7 | 9.5 | 0.052 | -0.1 | 19.0 |
| New delhi | 0.0 | 0.000 | 0.0 | 0.0 | 5.0 | 0.383 | -6.2 | 16.2 | 2.6 | 0.398 | -3.4 | 8.6 |
| Central | 21.3 | 0.074 | -2.1 | 44.6 | 0.0 | 0.000 | 0.0 | 0.0 | 11.0 | 0.028 | 1.2 | 20.9 |
| West | 17.8 | 0.079 | -2.0 | 37.6 | 3.3 | 0.309 | -3.1 | 9.7 | 9.7 | 0.106 | -2.1 | 21.5 |
| South west | 3.0 | 0.389 | -3.8 | 9.7 | 4.5 | 0.085 | -0.6 | 9.7 | 3.8 | 0.024 | 0.5 | 7.2 |
| South | 10.0 | 0.209 | -5.6 | 25.7 | 24.6 | 0.018 | 4.2 | 45.0 | 18.4 | 0.018 | 3.1 | 33.7 |
| Ganganagar | 31.9 | 0.001 | 12.4 | 51.5 | 36.4 | 0.001 | 15.2 | 57.6 | 34.3 | 0.000 | 22.2 | 46.4 |
| Hanumangarh | 18.2 | 0.007 | 4.9 | 31.4 | 35.2 | 0.000 | 15.8 | 54.7 | 27.5 | 0.000 | 17.1 | 37.8 |
| Bikaner | 25.1 | 0.000 | 16.7 | 33.4 | 37.8 | 0.000 | 23.0 | 52.6 | 31.6 | 0.000 | 23.6 | 39.7 |
| Churu | 22.1 | 0.002 | 8.0 | 36.2 | 14.1 | 0.005 | 4.3 | 23.9 | 17.9 | 0.000 | 10.9 | 24.9 |
| Jhunjhunun | 18.7 | 0.001 | 7.4 | 30.0 | 28.8 | 0.000 | 14.3 | 43.2 | 24.2 | 0.000 | 13.4 | 35.1 |
| Alwar | 24.1 | 0.000 | 11.2 | 37.0 | 23.3 | 0.002 | 8.6 | 38.0 | 23.7 | 0.000 | 14.9 | 32.4 |
| Bharatpur | 21.9 | 0.001 | 9.4 | 34.3 | 25.3 | 0.000 | 13.6 | 37.0 | 23.6 | 0.000 | 14.8 | 32.3 |
| Dhaulpur | 24.2 | 0.002 | 9.2 | 39.3 | 30.9 | 0.000 | 15.1 | 46.8 | 27.9 | 0.000 | 19.0 | 36.7 |
| Karauli | 45.4 | 0.000 | 24.8 | 66.0 | 41.4 | 0.000 | 24.5 | 58.3 | 43.3 | 0.000 | 29.5 | 57.0 |
| Sawai madhopur | 33.3 | 0.000 | 16.1 | 50.4 | 27.9 | 0.000 | 14.3 | 41.5 | 30.4 | 0.000 | 21.6 | 39.2 |
| Dausa | 36.2 | 0.000 | 21.7 | 50.7 | 28.4 | 0.000 | 14.3 | 42.5 | 32.2 | 0.000 | 21.4 | 43.0 |
| Jaipur | 29.2 | 0.000 | 19.1 | 39.3 | 25.1 | 0.000 | 15.5 | 34.8 | 27.0 | 0.000 | 19.8 | 34.2 |
| Sikar | 25.2 | 0.002 | 9.1 | 41.3 | 39.0 | 0.000 | 24.3 | 53.8 | 32.5 | 0.000 | 19.7 | 45.3 |
| Nagaur | 12.7 | 0.048 | 0.1 | 25.2 | 32.3 | 0.000 | 16.6 | 48.0 | 23.2 | 0.000 | 13.3 | 33.2 |
| Jodhpur | 31.8 | 0.000 | 17.9 | 45.6 | 39.2 | 0.000 | 25.7 | 52.8 | 35.8 | 0.000 | 27.2 | 44.5 |
| Jaisalmer | 35.3 | 0.000 | 21.2 | 49.5 | 33.0 | 0.000 | 18.3 | 47.7 | 34.0 | 0.000 | 19.6 | 48.4 |
| Barmer | 44.9 | 0.000 | 27.7 | 62.1 | 40.1 | 0.000 | 24.6 | 55.6 | 42.4 | 0.000 | 30.4 | 54.4 |
| Jalor | 34.1 | 0.000 | 16.8 | 51.4 | 51.9 | 0.000 | 34.9 | 68.9 | 43.9 | 0.000 | 33.0 | 54.8 |
| Sirohi | 40.4 | 0.000 | 19.8 | 61.0 | 39.9 | 0.000 | 22.7 | 57.0 | 40.1 | 0.000 | 25.8 | 54.5 |
| Pali | 39.7 | 0.000 | 20.9 | 58.5 | 40.6 | 0.000 | 24.6 | 56.5 | 40.2 | 0.000 | 25.3 | 55.0 |
| Ajmer | 22.8 | 0.000 | 12.6 | 33.0 | 25.6 | 0.000 | 14.8 | 36.4 | 24.3 | 0.000 | 16.2 | 32.4 |
| Tonk | 42.3 | 0.000 | 20.2 | 64.3 | 22.8 | 0.007 | 6.1 | 39.4 | 32.4 | 0.000 | 20.7 | 44.1 |
| Bundi | 31.7 | 0.001 | 12.3 | 51.1 | 28.5 | 0.002 | 10.9 | 46.2 | 30.0 | 0.000 | 17.5 | 42.5 |
| Bhilwara | 35.3 | 0.000 | 18.2 | 52.4 | 35.1 | 0.001 | 13.5 | 56.7 | 35.2 | 0.000 | 23.0 | 47.4 |
| Rajsamand | 24.2 | 0.000 | 10.8 | 37.6 | 42.8 | 0.000 | 22.7 | 62.8 | 34.6 | 0.000 | 22.2 | 46.9 |
| Dungarpur | 20.3 | 0.002 | 7.5 | 33.1 | 23.8 | 0.000 | 12.3 | 35.4 | 22.2 | 0.000 | 13.3 | 31.1 |
| Banswara | 17.1 | 0.006 | 5.0 | 29.1 | 27.4 | 0.000 | 15.1 | 39.7 | 22.3 | 0.000 | 13.4 | 31.3 |
| Chittaurgarh | 30.5 | 0.001 | 12.3 | 48.8 | 53.7 | 0.000 | 28.4 | 79.1 | 42.8 | 0.000 | 27.1 | 58.5 |
| Kota | 23.4 | 0.000 | 11.7 | 35.0 | 31.1 | 0.000 | 20.3 | 41.9 | 27.4 | 0.000 | 19.9 | 35.0 |
| Baran | 30.3 | 0.003 | 10.5 | 50.0 | 32.3 | 0.001 | 13.7 | 50.9 | 31.3 | 0.000 | 19.8 | 42.9 |
| Jhalawar | 23.8 | 0.001 | 9.5 | 38.1 | 49.1 | 0.000 | 30.0 | 68.2 | 37.9 | 0.000 | 26.2 | 49.7 |
| Udaipur | 28.2 | 0.001 | 11.3 | 45.1 | 34.8 | 0.000 | 16.4 | 53.2 | 31.5 | 0.000 | 20.4 | 42.7 |
| Pratapgarh | 33.2 | 0.000 | 16.3 | 50.0 | 27.1 | 0.000 | 13.9 | 40.2 | 29.9 | 0.000 | 20.3 | 39.5 |
| Saharanpur | 44.5 | 0.000 | 29.2 | 59.8 | 30.9 | 0.000 | 21.4 | 40.4 | 37.3 | 0.000 | 29.8 | 44.8 |
| Muzaffarnagar | 48.3 | 0.000 | 26.9 | 69.6 | 47.5 | 0.000 | 32.2 | 62.7 | 47.8 | 0.000 | 35.6 | 60.1 |
| Bijnor | 41.8 | 0.000 | 26.7 | 57.0 | 30.4 | 0.000 | 17.4 | 43.4 | 35.7 | 0.000 | 25.0 | 46.5 |
| Moradabad | 57.1 | 0.000 | 44.2 | 70.0 | 60.4 | 0.000 | 44.7 | 76.2 | 58.8 | 0.000 | 47.9 | 69.8 |
| Rampur | 37.8 | 0.000 | 23.8 | 51.8 | 38.6 | 0.000 | 24.5 | 52.6 | 38.2 | 0.000 | 26.8 | 49.6 |
| Jyotiba phule nagar | 44.8 | 0.000 | 27.8 | 61.8 | 39.8 | 0.000 | 25.0 | 54.6 | 42.3 | 0.000 | 32.4 | 52.1 |
| Meerut | 35.5 | 0.000 | 20.8 | 50.1 | 35.7 | 0.000 | 23.8 | 47.6 | 35.6 | 0.000 | 25.0 | 46.2 |
| Baghpat | 36.5 | 0.000 | 20.4 | 52.6 | 25.2 | 0.000 | 13.3 | 37.1 | 30.3 | 0.000 | 20.7 | 39.9 |
| Ghaziabad | 18.1 | 0.000 | 9.7 | 26.5 | 39.3 | 0.000 | 24.7 | 53.8 | 29.3 | 0.000 | 21.0 | 37.7 |
| Gautam buddha nagar | 33.1 | 0.000 | 22.7 | 43.6 | 36.1 | 0.000 | 24.6 | 47.5 | 34.7 | 0.000 | 26.4 | 43.0 |
| Bulandshahr | 33.8 | 0.000 | 19.5 | 48.1 | 54.3 | 0.000 | 36.1 | 72.6 | 44.6 | 0.000 | 28.9 | 60.4 |
| Aligarh | 43.4 | 0.000 | 29.5 | 57.3 | 63.2 | 0.000 | 47.3 | 79.1 | 54.0 | 0.000 | 44.3 | 63.7 |
| Mahamaya nagar | 44.4 | 0.000 | 26.4 | 62.3 | 51.5 | 0.000 | 32.0 | 70.9 | 48.2 | 0.000 | 32.6 | 63.8 |
| Mathura | 39.0 | 0.000 | 22.3 | 55.6 | 50.3 | 0.000 | 33.7 | 66.9 | 45.0 | 0.000 | 32.1 | 58.0 |
| Agra | 32.5 | 0.000 | 21.7 | 43.4 | 46.4 | 0.000 | 33.1 | 59.7 | 39.6 | 0.000 | 32.1 | 47.1 |
| Firozabad | 46.3 | 0.000 | 33.0 | 59.6 | 44.0 | 0.000 | 32.7 | 55.4 | 45.1 | 0.000 | 37.3 | 53.0 |
| Mainpuri | 49.8 | 0.000 | 30.8 | 68.8 | 54.6 | 0.000 | 37.6 | 71.5 | 52.3 | 0.000 | 40.8 | 63.8 |
| Budaun | 58.8 | 0.000 | 40.5 | 77.1 | 74.7 | 0.000 | 55.7 | 93.6 | 67.1 | 0.000 | 52.1 | 82.1 |
| Bareilly | 42.4 | 0.000 | 30.9 | 54.0 | 43.3 | 0.000 | 30.5 | 56.1 | 42.9 | 0.000 | 35.3 | 50.5 |
| Pilibhit | 42.5 | 0.000 | 28.1 | 56.9 | 52.2 | 0.000 | 31.4 | 73.0 | 47.6 | 0.000 | 36.1 | 59.0 |
| Shahjahanpur | 35.1 | 0.000 | 20.7 | 49.5 | 82.4 | 0.000 | 60.6 | 104.2 | 59.0 | 0.000 | 44.5 | 73.5 |
| Kheri | 63.2 | 0.000 | 41.6 | 84.8 | 50.6 | 0.000 | 32.6 | 68.5 | 56.7 | 0.000 | 43.7 | 69.8 |
| Sitapur | 54.4 | 0.000 | 35.9 | 72.9 | 62.9 | 0.000 | 44.7 | 81.1 | 59.0 | 0.000 | 43.6 | 74.3 |
| Hardoi | 39.3 | 0.000 | 20.1 | 58.6 | 60.7 | 0.000 | 42.2 | 79.1 | 51.1 | 0.000 | 38.5 | 63.6 |
| Unnao | 38.4 | 0.000 | 22.4 | 54.4 | 39.5 | 0.000 | 23.6 | 55.3 | 39.0 | 0.000 | 26.3 | 51.6 |
| Lucknow | 38.3 | 0.000 | 20.1 | 56.4 | 26.5 | 0.000 | 11.8 | 41.1 | 32.0 | 0.000 | 21.5 | 42.5 |

| | | | | | | | | | | | | |
|---|---|---|---|---|---|---|---|---|---|---|---|---|
| Rae bareli | 28.5 | 0.001 | 11.1 | 46.0 | 46.7 | 0.000 | 27.3 | 66.2 | 37.9 | 0.000 | 23.2 | 52.5 |
| Farrukhabad | 45.1 | 0.000 | 29.2 | 61.0 | 60.0 | 0.000 | 39.8 | 80.3 | 52.4 | 0.000 | 40.4 | 64.4 |
| Kannauj | 56.5 | 0.000 | 36.4 | 76.6 | 51.6 | 0.000 | 33.9 | 69.2 | 54.0 | 0.000 | 40.1 | 67.8 |
| Etawah | 36.4 | 0.000 | 17.9 | 55.0 | 48.3 | 0.000 | 31.5 | 65.2 | 43.2 | 0.000 | 27.4 | 59.0 |
| Auraiya | 33.0 | 0.001 | 13.3 | 52.6 | 37.7 | 0.000 | 21.1 | 54.3 | 35.5 | 0.000 | 23.5 | 47.4 |
| Kanpur dehat | 25.9 | 0.001 | 10.7 | 41.1 | 40.8 | 0.000 | 23.1 | 58.4 | 34.0 | 0.000 | 24.0 | 43.9 |
| Kanpur nagar | 32.8 | 0.003 | 10.8 | 54.7 | 39.1 | 0.000 | 17.9 | 60.3 | 36.2 | 0.000 | 22.7 | 49.8 |
| Jalaun | 42.5 | 0.005 | 12.8 | 72.3 | 49.8 | 0.000 | 26.2 | 73.4 | 46.7 | 0.000 | 27.1 | 66.3 |
| Jhansi | 51.9 | 0.000 | 32.2 | 71.6 | 54.6 | 0.000 | 35.6 | 73.5 | 53.3 | 0.000 | 39.0 | 67.6 |
| Lalitpur | 32.8 | 0.001 | 13.8 | 51.7 | 38.6 | 0.001 | 16.7 | 60.5 | 35.8 | 0.000 | 21.8 | 49.8 |
| Hamirpur | 31.5 | 0.003 | 10.9 | 52.1 | 24.0 | 0.002 | 9.1 | 39.0 | 27.3 | 0.000 | 14.1 | 40.6 |
| Mahoba | 36.1 | 0.000 | 20.3 | 51.9 | 47.4 | 0.000 | 27.4 | 67.4 | 41.9 | 0.000 | 26.9 | 57.0 |
| Banda | 21.5 | 0.012 | 4.7 | 38.3 | 33.7 | 0.001 | 13.3 | 54.2 | 27.9 | 0.000 | 13.8 | 42.0 |
| Chitrakoot | 29.1 | 0.000 | 16.8 | 41.3 | 46.9 | 0.000 | 29.0 | 64.9 | 38.2 | 0.000 | 26.7 | 49.8 |
| Fatehpur | 35.3 | 0.000 | 17.3 | 53.2 | 38.1 | 0.000 | 23.9 | 52.3 | 36.6 | 0.000 | 24.6 | 48.7 |
| Pratapgarh | 31.1 | 0.001 | 12.2 | 49.9 | 53.4 | 0.000 | 30.3 | 76.4 | 43.0 | 0.000 | 28.9 | 57.2 |
| Kaushambi | 59.1 | 0.000 | 38.5 | 79.8 | 83.4 | 0.000 | 58.4 | 108.4 | 71.6 | 0.000 | 56.6 | 86.6 |
| Allahabad | 26.2 | 0.000 | 12.8 | 39.6 | 44.0 | 0.000 | 23.8 | 64.1 | 35.1 | 0.000 | 25.3 | 44.9 |
| Bara banki | 14.5 | 0.009 | 3.6 | 25.5 | 34.3 | 0.000 | 16.9 | 51.7 | 24.3 | 0.000 | 14.1 | 34.5 |
| Faizabad | 41.1 | 0.000 | 22.5 | 59.7 | 43.6 | 0.000 | 27.0 | 60.2 | 42.4 | 0.000 | 29.6 | 55.2 |
| Ambedkar nagar | 45.8 | 0.000 | 23.2 | 68.5 | 52.7 | 0.000 | 35.2 | 70.1 | 49.3 | 0.000 | 35.1 | 63.6 |
| Sultanpur | 42.6 | 0.000 | 20.5 | 64.7 | 51.0 | 0.000 | 33.4 | 68.6 | 47.0 | 0.000 | 31.5 | 62.6 |
| Bahraich | 58.8 | 0.000 | 40.9 | 76.6 | 45.1 | 0.000 | 32.7 | 57.4 | 51.8 | 0.000 | 39.8 | 63.8 |
| Shrawasti | 49.1 | 0.000 | 32.2 | 66.1 | 68.2 | 0.000 | 50.0 | 86.5 | 59.0 | 0.000 | 47.3 | 70.8 |
| Balrampur | 45.0 | 0.000 | 31.2 | 58.8 | 41.8 | 0.000 | 27.0 | 56.6 | 43.3 | 0.000 | 32.1 | 54.5 |
| Gonda | 69.6 | 0.000 | 52.0 | 87.3 | 97.7 | 0.000 | 70.9 | 124.5 | 84.0 | 0.000 | 66.7 | 101.3 |
| Siddharth nagar | 43.5 | 0.000 | 27.3 | 59.8 | 62.1 | 0.000 | 47.7 | 76.6 | 53.4 | 0.000 | 42.6 | 64.1 |
| Basti | 43.4 | 0.000 | 24.8 | 61.9 | 53.6 | 0.000 | 34.9 | 72.3 | 48.8 | 0.000 | 38.3 | 59.3 |
| Sant kabir nagar | 39.6 | 0.000 | 23.0 | 56.2 | 45.6 | 0.000 | 30.8 | 60.3 | 42.9 | 0.000 | 31.3 | 54.4 |
| Mahrajganj | 32.4 | 0.000 | 15.5 | 49.3 | 67.2 | 0.000 | 51.4 | 83.0 | 51.3 | 0.000 | 38.9 | 63.7 |
| Gorakhpur | 33.4 | 0.000 | 17.5 | 49.2 | 49.8 | 0.000 | 31.1 | 68.5 | 41.9 | 0.000 | 28.2 | 55.6 |
| Kushinagar | 27.3 | 0.000 | 13.9 | 40.7 | 46.6 | 0.000 | 32.7 | 60.5 | 37.8 | 0.000 | 27.0 | 48.6 |
| Deoria | 29.4 | 0.000 | 14.3 | 44.5 | 44.3 | 0.000 | 30.3 | 58.3 | 37.2 | 0.000 | 25.8 | 48.7 |
| Azamgarh | 48.9 | 0.000 | 30.8 | 67.0 | 42.7 | 0.000 | 23.2 | 62.3 | 45.8 | 0.000 | 34.0 | 57.5 |
| Mau | 26.9 | 0.000 | 13.6 | 40.1 | 58.7 | 0.000 | 43.3 | 74.2 | 43.3 | 0.000 | 31.1 | 55.4 |
| Ballia | 33.4 | 0.000 | 15.7 | 51.2 | 37.7 | 0.000 | 20.5 | 54.9 | 35.6 | 0.000 | 24.6 | 46.6 |
| Jaunpur | 31.6 | 0.000 | 18.8 | 44.4 | 48.6 | 0.000 | 29.7 | 67.4 | 40.2 | 0.000 | 28.4 | 52.1 |
| Ghazipur | 42.0 | 0.000 | 24.7 | 59.4 | 48.2 | 0.000 | 29.4 | 67.0 | 45.1 | 0.000 | 32.0 | 58.1 |
| Chandauli | 24.8 | 0.000 | 14.4 | 35.3 | 33.4 | 0.000 | 17.8 | 49.0 | 29.3 | 0.000 | 19.6 | 38.9 |
| Varanasi | 42.4 | 0.000 | 29.0 | 55.7 | 39.9 | 0.000 | 26.2 | 53.6 | 41.1 | 0.000 | 30.4 | 51.8 |
| Sant ravidas nagar (bhadohi) | 47.3 | 0.000 | 29.4 | 65.2 | 77.3 | 0.000 | 57.5 | 97.1 | 62.7 | 0.000 | 47.9 | 77.6 |
| Mirzapur | 51.1 | 0.000 | 31.1 | 71.1 | 45.7 | 0.000 | 27.5 | 63.9 | 48.3 | 0.000 | 37.3 | 59.3 |
| Sonbhadra | 25.1 | 0.000 | 12.6 | 37.5 | 38.7 | 0.000 | 25.3 | 52.0 | 32.4 | 0.000 | 22.0 | 42.8 |
| Etah | 46.4 | 0.000 | 25.8 | 67.0 | 75.3 | 0.000 | 52.9 | 97.7 | 62.1 | 0.000 | 50.5 | 73.6 |
| Kanshiram nagar | 53.6 | 0.000 | 32.5 | 74.8 | 64.3 | 0.000 | 45.3 | 83.4 | 59.4 | 0.000 | 47.8 | 70.9 |
| Pashchim champaran | 25.3 | 0.000 | 17.3 | 33.2 | 38.7 | 0.000 | 24.6 | 52.8 | 32.1 | 0.000 | 22.9 | 41.3 |
| Purba champaran | 35.8 | 0.000 | 22.6 | 49.1 | 36.2 | 0.000 | 21.6 | 50.9 | 36.0 | 0.000 | 25.5 | 46.5 |
| Sheohar | 43.0 | 0.000 | 28.4 | 57.6 | 53.0 | 0.000 | 36.7 | 69.2 | 48.3 | 0.000 | 39.5 | 57.1 |
| Sitamarhi | 35.9 | 0.000 | 20.6 | 51.3 | 52.7 | 0.000 | 34.4 | 71.1 | 44.6 | 0.000 | 31.0 | 58.2 |
| Madhubani | 27.2 | 0.000 | 14.3 | 40.1 | 35.7 | 0.000 | 22.3 | 49.1 | 31.6 | 0.000 | 21.6 | 41.6 |
| Supaul | 29.8 | 0.000 | 19.1 | 40.5 | 37.3 | 0.000 | 24.1 | 50.5 | 33.6 | 0.000 | 26.2 | 41.0 |
| Araria | 37.3 | 0.000 | 22.0 | 52.6 | 58.0 | 0.000 | 42.7 | 73.3 | 48.1 | 0.000 | 36.3 | 59.9 |
| Kishanganj | 23.5 | 0.000 | 12.1 | 34.8 | 36.0 | 0.000 | 20.7 | 51.4 | 30.3 | 0.000 | 20.7 | 40.0 |
| Purnia | 43.1 | 0.000 | 29.8 | 56.4 | 55.2 | 0.000 | 42.3 | 68.2 | 49.3 | 0.000 | 38.2 | 60.5 |
| Katihar | 36.9 | 0.000 | 21.2 | 52.6 | 66.1 | 0.000 | 45.9 | 86.3 | 52.0 | 0.000 | 39.7 | 64.3 |
| Madhepura | 40.7 | 0.000 | 26.0 | 55.4 | 40.9 | 0.000 | 28.0 | 53.9 | 40.8 | 0.000 | 31.9 | 49.8 |
| Saharsa | 25.6 | 0.000 | 15.3 | 35.8 | 52.8 | 0.000 | 38.3 | 67.3 | 39.1 | 0.000 | 29.5 | 48.7 |
| Darbhanga | 17.4 | 0.000 | 8.0 | 26.7 | 26.5 | 0.000 | 15.1 | 37.8 | 22.0 | 0.000 | 15.1 | 28.9 |
| Muzaffarpur | 31.5 | 0.000 | 16.0 | 47.0 | 29.6 | 0.000 | 15.8 | 43.4 | 30.5 | 0.000 | 21.4 | 39.6 |
| Gopalganj | 25.1 | 0.000 | 12.5 | 37.7 | 43.4 | 0.000 | 28.0 | 58.8 | 34.2 | 0.000 | 23.2 | 45.2 |
| Siwan | 29.8 | 0.000 | 15.0 | 44.5 | 40.9 | 0.000 | 27.5 | 54.2 | 35.6 | 0.000 | 26.0 | 45.2 |
| Saran | 20.9 | 0.001 | 8.5 | 33.2 | 30.7 | 0.000 | 17.3 | 44.1 | 25.9 | 0.000 | 18.7 | 33.1 |
| Vaishali | 25.1 | 0.000 | 12.2 | 37.9 | 35.9 | 0.000 | 21.1 | 50.8 | 31.0 | 0.000 | 21.2 | 40.9 |
| Samastipur | 29.9 | 0.000 | 15.2 | 44.6 | 23.3 | 0.000 | 13.4 | 33.2 | 26.5 | 0.000 | 19.1 | 33.9 |
| Begusarai | 23.6 | 0.002 | 8.8 | 38.4 | 40.4 | 0.000 | 24.0 | 56.8 | 32.4 | 0.000 | 24.0 | 40.7 |
| Khagaria | 27.6 | 0.000 | 16.4 | 38.7 | 44.6 | 0.000 | 31.2 | 58.0 | 36.3 | 0.000 | 27.9 | 44.6 |
| Bhagalpur | 9.9 | 0.002 | 3.7 | 16.1 | 26.5 | 0.000 | 14.6 | 38.4 | 18.7 | 0.000 | 11.3 | 26.1 |
| Banka | 32.2 | 0.000 | 17.4 | 47.0 | 34.3 | 0.000 | 22.0 | 46.6 | 33.3 | 0.000 | 23.7 | 43.0 |
| Munger | 39.8 | 0.000 | 24.0 | 55.6 | 45.8 | 0.000 | 29.3 | 62.3 | 42.9 | 0.000 | 31.3 | 54.6 |
| Lakhisarai | 37.9 | 0.000 | 23.9 | 51.9 | 45.5 | 0.000 | 31.3 | 59.7 | 41.9 | 0.000 | 32.3 | 51.4 |
| Sheikhpura | 35.5 | 0.000 | 21.8 | 49.2 | 37.4 | 0.000 | 23.1 | 51.6 | 36.5 | 0.000 | 25.8 | 47.2 |
| Nalanda | 25.7 | 0.000 | 12.8 | 38.6 | 27.7 | 0.000 | 13.9 | 41.6 | 26.7 | 0.000 | 17.7 | 35.7 |
| Patna | 28.5 | 0.000 | 20.7 | 36.3 | 34.1 | 0.000 | 22.8 | 45.4 | 31.3 | 0.000 | 24.8 | 37.9 |
| Bhojpur | 39.7 | 0.000 | 25.8 | 53.5 | 32.6 | 0.000 | 17.3 | 47.8 | 36.0 | 0.000 | 25.6 | 46.4 |
| Buxar | 31.8 | 0.000 | 16.9 | 46.8 | 43.5 | 0.000 | 31.4 | 55.7 | 37.9 | 0.000 | 26.2 | 49.5 |
| Kaimur (bhabua) | 27.3 | 0.000 | 15.8 | 38.7 | 58.3 | 0.000 | 40.0 | 76.7 | 43.0 | 0.000 | 31.8 | 54.1 |
| Rohtas | 25.6 | 0.000 | 15.0 | 36.3 | 52.9 | 0.000 | 35.4 | 70.4 | 39.5 | 0.000 | 28.9 | 50.1 |
| Aurangabad | 42.3 | 0.000 | 26.2 | 58.4 | 26.8 | 0.000 | 13.1 | 40.4 | 34.1 | 0.000 | 23.7 | 44.5 |
| Gaya | 48.0 | 0.000 | 30.6 | 65.4 | 55.4 | 0.000 | 39.7 | 71.1 | 51.9 | 0.000 | 41.0 | 62.7 |
| Nawada | 31.9 | 0.000 | 18.6 | 45.3 | 34.4 | 0.000 | 21.7 | 47.0 | 33.2 | 0.000 | 23.2 | 43.1 |
| Jamui | 38.6 | 0.000 | 26.2 | 51.0 | 50.1 | 0.000 | 35.4 | 64.9 | 44.6 | 0.000 | 35.5 | 53.7 |

| | | | | | | | | | | | | |
|---|---|---|---|---|---|---|---|---|---|---|---|---|
| Jehanabad | 40.2 | 0.000 | 19.6 | 60.9 | 46.6 | 0.000 | 32.3 | 60.9 | 43.6 | 0.000 | 27.9 | 59.4 |
| Arwal | 32.4 | 0.000 | 19.9 | 44.9 | 35.4 | 0.000 | 20.4 | 50.3 | 33.9 | 0.000 | 23.8 | 44.0 |
| North district | 14.7 | 0.144 | -5.0 | 34.3 | 8.4 | 0.091 | -1.3 | 18.1 | 11.2 | 0.020 | 1.7 | 20.7 |
| West district | 32.4 | 0.012 | 7.1 | 57.7 | 57.4 | 0.000 | 31.7 | 83.0 | 46.0 | 0.000 | 27.6 | 64.4 |
| South district | 2.2 | 0.304 | -2.0 | 6.5 | 7.8 | 0.166 | -3.2 | 18.7 | 5.0 | 0.087 | -0.7 | 10.7 |
| East district | 12.6 | 0.033 | 1.0 | 24.1 | 11.4 | 0.014 | 2.3 | 20.5 | 12.0 | 0.001 | 5.2 | 18.7 |
| Tawang | 19.5 | 0.037 | 1.2 | 37.9 | 2.4 | 0.312 | -2.2 | 6.9 | 10.3 | 0.039 | 0.5 | 20.1 |
| West kameng | 23.9 | 0.012 | 5.4 | 42.5 | 21.0 | 0.048 | 0.1 | 41.8 | 22.5 | 0.001 | 8.8 | 36.1 |
| East kameng | 4.3 | 0.065 | -0.3 | 8.9 | 9.6 | 0.010 | 2.3 | 16.9 | 7.0 | 0.007 | 1.9 | 12.1 |
| Papumpare | 10.1 | 0.157 | -3.9 | 24.1 | 7.5 | 0.083 | -1.0 | 15.9 | 8.7 | 0.037 | 0.5 | 16.9 |
| Upper subansiri | 3.2 | 0.244 | -2.2 | 8.6 | 6.7 | 0.137 | -2.1 | 15.6 | 5.0 | 0.050 | 0.0 | 10.0 |
| West siang | 0.0 | 0.000 | 0.0 | 0.0 | 3.5 | 0.371 | -4.1 | 11.1 | 1.9 | 0.423 | -2.7 | 6.5 |
| East siang | 0.0 | 0.000 | 0.0 | 0.0 | 2.8 | 0.300 | -2.5 | 8.0 | 1.5 | 0.303 | -1.3 | 4.2 |
| Upper siang | 0.0 | 0.000 | 0.0 | 0.0 | 0.0 | 0.000 | 0.0 | 0.0 | 0.0 | 0.000 | 0.0 | 0.0 |
| Changlang | 35.1 | 0.000 | 15.8 | 54.4 | 8.8 | 0.065 | -0.6 | 18.2 | 22.3 | 0.000 | 11.5 | 33.1 |
| Tirap | 18.9 | 0.016 | 3.5 | 34.4 | 12.4 | 0.036 | 0.8 | 24.1 | 15.4 | 0.001 | 6.3 | 24.6 |
| Lower subansiri | 15.8 | 0.028 | 1.7 | 29.9 | 9.8 | 0.148 | -3.5 | 23.2 | 12.8 | 0.015 | 2.5 | 23.0 |
| Kurung kumey | 6.0 | 0.120 | -1.5 | 13.5 | 3.7 | 0.169 | -1.6 | 9.0 | 4.8 | 0.023 | 0.7 | 9.0 |
| Dibang valley | 0.0 | 0.000 | 0.0 | 0.0 | 0.0 | 0.000 | 0.0 | 0.0 | 0.0 | 0.000 | 0.0 | 0.0 |
| Lower dibang valley | 3.3 | 0.315 | -3.1 | 9.7 | 5.9 | 0.146 | -2.1 | 13.9 | 4.7 | 0.065 | -0.3 | 9.7 |
| Lohit | 28.1 | 0.002 | 10.7 | 45.4 | 48.1 | 0.000 | 26.2 | 70.0 | 38.8 | 0.000 | 26.8 | 50.8 |
| Anjaw | 5.5 | 0.242 | -3.7 | 14.6 | 13.4 | 0.029 | 1.3 | 25.4 | 9.5 | 0.015 | 1.8 | 17.2 |
| Mon | 2.1 | 0.291 | -1.8 | 6.0 | 3.1 | 0.166 | -1.3 | 7.4 | 2.6 | 0.124 | -0.7 | 5.9 |
| Mokokchung | 24.3 | 0.043 | 0.8 | 47.8 | 23.4 | 0.051 | -0.1 | 46.9 | 23.8 | 0.009 | 6.0 | 41.7 |
| Zunheboto | 7.2 | 0.134 | -2.2 | 16.6 | 11.4 | 0.055 | -0.3 | 23.1 | 9.5 | 0.008 | 2.5 | 16.5 |
| Wokha | 20.9 | 0.015 | 4.1 | 37.8 | 22.1 | 0.016 | 4.1 | 40.2 | 21.6 | 0.004 | 6.8 | 36.4 |
| Dimapur | 23.2 | 0.000 | 11.4 | 35.0 | 21.3 | 0.001 | 8.2 | 34.3 | 22.2 | 0.000 | 13.6 | 30.8 |
| Phek | 7.4 | 0.081 | -0.9 | 15.8 | 8.7 | 0.015 | 1.7 | 15.7 | 8.1 | 0.019 | 1.3 | 14.8 |
| Tuensang | 14.7 | 0.009 | 3.6 | 25.8 | 13.2 | 0.015 | 2.6 | 23.9 | 14.0 | 0.000 | 6.6 | 21.3 |
| Longleng | 5.2 | 0.183 | -2.5 | 12.9 | 9.2 | 0.066 | -0.6 | 18.9 | 7.3 | 0.010 | 1.7 | 12.9 |
| Kiphire | 23.0 | 0.007 | 6.3 | 39.7 | 32.2 | 0.000 | 15.2 | 49.1 | 27.7 | 0.000 | 15.3 | 40.1 |
| Kohima | 8.9 | 0.010 | 2.1 | 15.7 | 16.1 | 0.009 | 4.1 | 28.2 | 12.3 | 0.000 | 5.6 | 19.1 |
| Peren | 12.3 | 0.020 | 2.0 | 22.7 | 27.6 | 0.000 | 14.1 | 41.1 | 20.3 | 0.000 | 11.2 | 29.4 |
| Senapati (excluding 3 sub-division | 31.1 | 0.000 | 15.3 | 47.0 | 26.2 | 0.001 | 11.1 | 41.2 | 28.5 | 0.000 | 17.3 | 39.7 |
| Tamenglong | 17.2 | 0.002 | 6.1 | 28.3 | 13.8 | 0.017 | 2.5 | 25.1 | 15.5 | 0.000 | 7.3 | 23.7 |
| Churachandpur | 8.1 | 0.104 | -1.7 | 17.9 | 27.9 | 0.001 | 11.4 | 44.3 | 18.3 | 0.000 | 9.4 | 27.1 |
| Bishnupur | 9.3 | 0.017 | 1.6 | 16.9 | 13.6 | 0.000 | 6.4 | 20.7 | 11.5 | 0.000 | 6.6 | 16.4 |
| Thoubal | 22.2 | 0.000 | 10.7 | 33.8 | 16.3 | 0.000 | 7.7 | 24.8 | 19.2 | 0.000 | 12.0 | 26.3 |
| Imphal west | 7.4 | 0.036 | 0.5 | 14.3 | 18.0 | 0.001 | 7.6 | 28.4 | 12.6 | 0.000 | 5.6 | 19.6 |
| Imphal east | 11.1 | 0.007 | 3.0 | 19.1 | 20.3 | 0.000 | 11.0 | 29.7 | 16.0 | 0.000 | 10.9 | 21.1 |
| Ukhrul | 19.9 | 0.005 | 6.1 | 33.8 | 17.8 | 0.002 | 6.5 | 29.1 | 18.9 | 0.000 | 9.4 | 28.3 |
| Chandel | 14.9 | 0.027 | 1.7 | 28.2 | 16.3 | 0.005 | 4.9 | 27.6 | 15.6 | 0.001 | 6.6 | 24.6 |
| Mamit | 9.6 | 0.018 | 1.6 | 17.5 | 7.1 | 0.128 | -2.1 | 16.3 | 8.3 | 0.008 | 2.2 | 14.5 |
| Kolasib | 8.8 | 0.019 | 1.5 | 16.2 | 9.3 | 0.006 | 2.6 | 16.0 | 9.1 | 0.001 | 3.6 | 14.5 |
| Aizawl | 28.8 | 0.023 | 3.9 | 53.7 | 17.6 | 0.005 | 5.3 | 29.9 | 23.0 | 0.000 | 11.5 | 34.4 |
| Champhai | 11.8 | 0.001 | 4.6 | 18.9 | 11.1 | 0.001 | 4.3 | 18.0 | 11.5 | 0.000 | 6.4 | 16.5 |
| Serchhip | 13.5 | 0.005 | 4.1 | 22.9 | 19.3 | 0.001 | 7.8 | 30.8 | 16.6 | 0.000 | 9.6 | 23.7 |
| Lunglei | 8.8 | 0.012 | 1.9 | 15.7 | 7.8 | 0.009 | 1.9 | 13.7 | 8.3 | 0.003 | 2.8 | 13.8 |
| Lawngtlai | 9.0 | 0.019 | 1.5 | 16.5 | 21.7 | 0.001 | 8.5 | 35.0 | 15.5 | 0.001 | 6.4 | 24.5 |
| Saiha | 9.6 | 0.002 | 3.6 | 15.6 | 18.3 | 0.000 | 9.7 | 27.0 | 14.1 | 0.000 | 7.5 | 20.7 |
| West tripura | 9.3 | 0.028 | 1.0 | 17.6 | 11.4 | 0.029 | 1.2 | 21.7 | 10.4 | 0.001 | 4.1 | 16.6 |
| South tripura | 15.4 | 0.042 | 0.6 | 30.1 | 3.6 | 0.214 | -2.1 | 9.2 | 9.2 | 0.038 | 0.5 | 17.8 |
| Dhalai | 9.5 | 0.089 | -1.4 | 20.5 | 18.4 | 0.006 | 5.3 | 31.5 | 14.0 | 0.005 | 4.2 | 23.8 |
| North tripura | 13.4 | 0.033 | 1.1 | 25.6 | 32.5 | 0.003 | 11.4 | 53.6 | 23.3 | 0.000 | 12.3 | 34.4 |
| West garo hills | 13.7 | 0.016 | 2.5 | 25.0 | 36.4 | 0.000 | 21.8 | 51.0 | 25.4 | 0.000 | 15.2 | 35.7 |
| East garo hills | 0.0 | 0.000 | 0.0 | 0.0 | 1.8 | 0.265 | -1.3 | 4.9 | 0.9 | 0.345 | -0.9 | 2.6 |
| South garo hills | 4.6 | 0.138 | -1.5 | 10.8 | 0.0 | 0.000 | 0.0 | 0.0 | 2.5 | 0.233 | -1.6 | 6.5 |
| West khasi hills | 19.5 | 0.000 | 8.9 | 30.1 | 26.4 | 0.000 | 14.5 | 38.3 | 22.9 | 0.000 | 14.6 | 31.3 |
| Ribhoi | 11.7 | 0.001 | 4.5 | 18.8 | 29.2 | 0.000 | 17.9 | 40.5 | 20.2 | 0.000 | 13.6 | 26.9 |
| East khasi hills | 17.6 | 0.000 | 8.8 | 26.4 | 19.4 | 0.000 | 10.6 | 28.1 | 18.5 | 0.000 | 12.9 | 24.1 |
| Jaintia hills | 19.7 | 0.000 | 9.3 | 30.0 | 16.7 | 0.004 | 5.4 | 28.0 | 18.1 | 0.000 | 11.6 | 24.7 |
| Kokrajhar | 22.5 | 0.003 | 7.6 | 37.4 | 38.4 | 0.000 | 18.4 | 58.3 | 30.8 | 0.000 | 20.1 | 41.5 |
| Dhubri | 16.1 | 0.002 | 6.0 | 26.2 | 41.3 | 0.000 | 24.4 | 58.2 | 29.2 | 0.000 | 18.8 | 39.6 |
| Goalpara | 22.8 | 0.003 | 8.0 | 37.6 | 25.5 | 0.001 | 10.3 | 40.6 | 24.2 | 0.000 | 13.8 | 34.7 |
| Barpeta | 17.1 | 0.003 | 5.9 | 28.4 | 32.1 | 0.000 | 16.5 | 47.6 | 24.9 | 0.000 | 15.0 | 34.8 |
| Morigaon | 31.0 | 0.000 | 14.6 | 47.3 | 53.5 | 0.000 | 30.6 | 76.5 | 42.6 | 0.000 | 29.6 | 55.5 |
| Nagaon | 47.2 | 0.000 | 29.8 | 64.6 | 41.7 | 0.000 | 23.3 | 60.1 | 44.4 | 0.000 | 29.9 | 58.8 |
| Sonitpur | 18.0 | 0.004 | 5.7 | 30.3 | 38.0 | 0.001 | 16.5 | 59.5 | 28.2 | 0.000 | 17.0 | 39.3 |
| Lakhimpur | 18.8 | 0.025 | 2.4 | 35.3 | 36.0 | 0.001 | 15.6 | 56.5 | 27.3 | 0.000 | 15.7 | 38.9 |
| Dhemaji | 34.7 | 0.000 | 18.0 | 51.4 | 57.7 | 0.000 | 37.9 | 77.5 | 46.6 | 0.000 | 32.1 | 61.2 |
| Tinsukia | 42.9 | 0.000 | 22.7 | 63.1 | 53.2 | 0.000 | 33.9 | 72.5 | 48.5 | 0.000 | 33.5 | 63.6 |
| Dibrugarh | 11.5 | 0.074 | -1.1 | 24.0 | 16.3 | 0.018 | 2.8 | 29.9 | 14.0 | 0.003 | 4.7 | 23.2 |
| Sivasagar | 46.0 | 0.000 | 24.1 | 67.9 | 40.0 | 0.002 | 14.3 | 65.7 | 43.1 | 0.000 | 29.5 | 56.8 |
| Jorhat | 32.4 | 0.001 | 14.1 | 50.8 | 39.2 | 0.001 | 15.8 | 62.5 | 36.0 | 0.000 | 21.0 | 50.9 |
| Golaghat | 29.9 | 0.001 | 11.9 | 47.9 | 43.9 | 0.000 | 26.0 | 61.9 | 37.0 | 0.000 | 22.7 | 51.3 |
| Karbi anglong | 30.7 | 0.000 | 14.6 | 46.7 | 50.5 | 0.000 | 26.8 | 74.3 | 40.9 | 0.000 | 27.7 | 54.1 |
| Dima hasao | 28.5 | 0.000 | 14.9 | 42.0 | 49.6 | 0.000 | 28.0 | 71.2 | 39.2 | 0.000 | 24.5 | 53.9 |
| Cachar | 56.0 | 0.000 | 30.6 | 81.4 | 42.6 | 0.000 | 23.3 | 61.9 | 49.2 | 0.000 | 33.2 | 65.1 |
| Karimganj | 40.8 | 0.000 | 23.2 | 58.3 | 52.6 | 0.000 | 34.1 | 71.0 | 47.0 | 0.000 | 33.1 | 60.9 |
| Hailakandi | 44.0 | 0.000 | 25.1 | 63.0 | 44.4 | 0.000 | 26.9 | 61.9 | 44.2 | 0.000 | 30.4 | 58.0 |
| Bongaigaon | 17.4 | 0.005 | 5.3 | 29.5 | 23.5 | 0.003 | 7.9 | 39.1 | 20.5 | 0.000 | 9.7 | 31.3 |

| | | | | | | | | | | | | |
|---|---|---|---|---|---|---|---|---|---|---|---|---|
| Chirang | 25.5 | 0.000 | 14.2 | 36.9 | 21.0 | 0.002 | 7.8 | 34.3 | 23.3 | 0.000 | 12.7 | 33.8 |
| Kamrup | 22.0 | 0.003 | 7.2 | 36.7 | 38.7 | 0.000 | 22.7 | 54.8 | 30.8 | 0.000 | 20.8 | 40.8 |
| Kamrup metropolitan | 31.8 | 0.006 | 8.9 | 54.7 | 26.5 | 0.009 | 6.7 | 46.4 | 29.1 | 0.000 | 14.9 | 43.3 |
| Nalbari | 17.5 | 0.035 | 1.2 | 33.9 | 26.0 | 0.018 | 4.5 | 47.5 | 22.0 | 0.000 | 11.4 | 32.7 |
| Baksa | 24.4 | 0.001 | 10.0 | 38.8 | 22.8 | 0.000 | 11.4 | 34.3 | 23.6 | 0.000 | 12.7 | 34.5 |
| Darrang | 20.4 | 0.004 | 6.3 | 34.5 | 53.6 | 0.000 | 29.8 | 77.4 | 37.5 | 0.000 | 24.5 | 50.6 |
| Udalguri | 20.6 | 0.003 | 7.2 | 34.0 | 22.2 | 0.002 | 7.9 | 36.6 | 21.4 | 0.000 | 11.2 | 31.7 |
| Darjiling | 16.6 | 0.071 | -1.4 | 34.5 | 13.7 | 0.106 | -2.9 | 30.4 | 15.1 | 0.018 | 2.6 | 27.7 |
| Jalpaiguri | 9.1 | 0.151 | -3.3 | 21.4 | 29.5 | 0.002 | 10.8 | 48.1 | 19.9 | 0.003 | 6.7 | 33.0 |
| Koch bihar | 18.0 | 0.042 | 0.7 | 35.4 | 33.9 | 0.002 | 12.3 | 55.5 | 26.0 | 0.001 | 10.4 | 41.6 |
| Uttar dinajpur | 32.7 | 0.000 | 15.2 | 50.2 | 47.8 | 0.000 | 29.8 | 65.8 | 40.6 | 0.000 | 27.9 | 53.3 |
| Dakshin dinajpur | 35.3 | 0.020 | 5.7 | 64.9 | 23.6 | 0.004 | 7.3 | 39.8 | 28.7 | 0.000 | 14.0 | 43.4 |
| Maldah | 24.7 | 0.002 | 9.3 | 40.1 | 33.3 | 0.000 | 16.0 | 50.6 | 29.3 | 0.000 | 16.8 | 41.8 |
| Murshidabad | 17.9 | 0.009 | 4.5 | 31.3 | 35.6 | 0.001 | 14.0 | 57.1 | 27.0 | 0.000 | 15.2 | 38.7 |
| Birbhum | 25.0 | 0.021 | 3.8 | 46.3 | 24.8 | 0.003 | 8.7 | 40.9 | 24.9 | 0.000 | 11.1 | 38.7 |
| Barddhaman | 17.8 | 0.013 | 3.8 | 31.9 | 28.4 | 0.039 | 1.5 | 55.4 | 23.1 | 0.001 | 9.8 | 36.3 |
| Nadia | 18.1 | 0.041 | 0.8 | 35.5 | 7.9 | 0.126 | -2.2 | 18.1 | 12.8 | 0.005 | 3.9 | 21.6 |
| North twenty four parganas | 7.9 | 0.142 | -2.7 | 18.4 | 21.9 | 0.013 | 4.7 | 39.2 | 15.4 | 0.022 | 2.3 | 28.5 |
| Hugli | 47.9 | 0.001 | 20.5 | 75.4 | 7.3 | 0.148 | -2.6 | 17.2 | 26.8 | 0.001 | 10.8 | 42.9 |
| Bankura | 30.6 | 0.005 | 9.1 | 52.1 | 50.0 | 0.000 | 24.2 | 75.7 | 39.7 | 0.000 | 24.2 | 55.2 |
| Puruliya | 21.8 | 0.001 | 8.8 | 34.8 | 24.5 | 0.004 | 7.8 | 41.1 | 23.2 | 0.000 | 11.8 | 34.7 |
| Haora | 16.6 | 0.013 | 3.5 | 29.6 | 35.2 | 0.004 | 11.3 | 59.0 | 26.0 | 0.000 | 12.9 | 39.1 |
| Kolkata | 5.1 | 0.304 | -4.6 | 14.8 | 1.9 | 0.285 | -1.6 | 5.5 | 3.5 | 0.262 | -2.6 | 9.5 |
| South twenty four parganas | 18.1 | 0.012 | 4.0 | 32.2 | 48.8 | 0.001 | 20.4 | 77.2 | 33.1 | 0.000 | 20.4 | 45.7 |
| Paschim medinipur | 9.0 | 0.184 | -4.3 | 22.2 | 24.2 | 0.023 | 3.3 | 45.1 | 17.0 | 0.005 | 5.2 | 28.9 |
| Purba medinipur | 15.4 | 0.014 | 3.2 | 27.6 | 3.0 | 0.280 | -2.4 | 8.4 | 9.0 | 0.005 | 2.8 | 15.3 |
| Garhwa | 40.6 | 0.000 | 21.1 | 60.2 | 41.6 | 0.000 | 24.9 | 58.3 | 41.1 | 0.000 | 28.1 | 54.1 |
| Chatra | 46.5 | 0.000 | 30.2 | 62.8 | 57.8 | 0.000 | 40.1 | 75.4 | 52.3 | 0.000 | 40.6 | 64.0 |
| Kodarma | 18.0 | 0.001 | 7.7 | 28.4 | 37.4 | 0.000 | 18.4 | 56.4 | 28.0 | 0.000 | 16.2 | 39.8 |
| Giridih | 36.8 | 0.000 | 20.1 | 53.5 | 43.1 | 0.000 | 25.8 | 60.4 | 40.1 | 0.000 | 27.4 | 52.9 |
| Deoghar | 48.6 | 0.000 | 32.2 | 65.1 | 35.0 | 0.000 | 21.5 | 48.5 | 41.2 | 0.000 | 28.5 | 53.9 |
| Godda | 36.5 | 0.000 | 20.9 | 52.1 | 56.1 | 0.000 | 33.3 | 78.9 | 46.7 | 0.000 | 31.2 | 62.2 |
| Sahibganj | 29.9 | 0.000 | 13.8 | 46.0 | 32.0 | 0.000 | 19.5 | 44.5 | 31.0 | 0.000 | 20.8 | 41.2 |
| Pakur | 21.5 | 0.000 | 10.8 | 32.2 | 35.2 | 0.000 | 19.5 | 50.9 | 28.4 | 0.000 | 18.7 | 38.1 |
| Dhanbad | 31.3 | 0.000 | 19.0 | 43.5 | 46.7 | 0.000 | 31.7 | 61.7 | 39.5 | 0.000 | 28.7 | 50.3 |
| Bokaro | 27.9 | 0.000 | 15.1 | 40.8 | 25.8 | 0.000 | 14.4 | 37.2 | 26.9 | 0.000 | 18.4 | 35.4 |
| Lohardaga | 23.1 | 0.001 | 9.6 | 36.7 | 26.9 | 0.001 | 11.7 | 42.1 | 25.0 | 0.000 | 14.3 | 35.7 |
| Purbi singhbhum | 10.9 | 0.016 | 2.0 | 19.8 | 7.2 | 0.039 | 0.4 | 14.0 | 9.0 | 0.000 | 4.1 | 13.9 |
| Palamu | 32.0 | 0.000 | 18.0 | 46.0 | 40.8 | 0.000 | 22.7 | 59.0 | 36.6 | 0.000 | 24.0 | 49.2 |
| Latehar | 15.7 | 0.018 | 2.7 | 28.7 | 24.9 | 0.002 | 9.5 | 40.4 | 20.5 | 0.000 | 11.4 | 29.6 |
| Hazaribagh | 37.2 | 0.000 | 19.8 | 54.6 | 34.3 | 0.000 | 20.1 | 48.6 | 35.6 | 0.000 | 23.8 | 47.5 |
| Ramgarh | 27.7 | 0.000 | 16.7 | 38.7 | 26.6 | 0.000 | 16.6 | 36.5 | 27.1 | 0.000 | 18.1 | 36.1 |
| Dumka | 34.8 | 0.001 | 13.5 | 56.2 | 35.3 | 0.000 | 19.3 | 51.2 | 35.0 | 0.000 | 24.1 | 46.0 |
| Jamtara | 32.2 | 0.000 | 16.6 | 47.9 | 39.5 | 0.000 | 25.2 | 53.7 | 36.1 | 0.000 | 25.3 | 46.9 |
| Ranchi | 32.5 | 0.000 | 17.4 | 47.6 | 35.4 | 0.000 | 17.5 | 53.3 | 33.9 | 0.000 | 24.5 | 43.4 |
| Khunti | 27.8 | 0.002 | 10.2 | 45.5 | 8.9 | 0.044 | 0.2 | 17.5 | 18.3 | 0.000 | 8.1 | 28.4 |
| Gumla | 25.7 | 0.001 | 10.3 | 41.1 | 32.6 | 0.000 | 15.5 | 49.7 | 29.2 | 0.000 | 17.3 | 41.1 |
| Simdega | 52.5 | 0.000 | 32.2 | 72.8 | 48.1 | 0.000 | 28.1 | 68.1 | 50.4 | 0.000 | 35.1 | 65.6 |
| Pashchimi singhbhum | 33.1 | 0.001 | 13.8 | 52.3 | 39.5 | 0.000 | 21.1 | 57.9 | 36.4 | 0.000 | 23.8 | 49.0 |
| Saraikela kharsawan | 26.1 | 0.021 | 3.9 | 48.2 | 37.1 | 0.000 | 21.3 | 52.9 | 31.8 | 0.000 | 17.6 | 46.1 |
| Bargarh | 20.8 | 0.010 | 5.0 | 36.6 | 25.7 | 0.005 | 7.6 | 43.9 | 23.3 | 0.000 | 10.4 | 36.3 |
| Jharsuguda | 36.2 | 0.000 | 22.0 | 50.4 | 33.6 | 0.000 | 18.9 | 48.4 | 34.9 | 0.000 | 25.5 | 44.3 |
| Sambalpur | 39.6 | 0.002 | 14.9 | 64.4 | 44.7 | 0.000 | 22.7 | 66.6 | 42.3 | 0.000 | 27.1 | 57.5 |
| Debagarh | 22.9 | 0.001 | 9.5 | 36.3 | 57.2 | 0.000 | 30.4 | 84.0 | 39.4 | 0.000 | 24.4 | 54.3 |
| Sundargarh | 16.0 | 0.000 | 7.8 | 24.2 | 34.7 | 0.000 | 18.6 | 50.9 | 25.2 | 0.000 | 16.1 | 34.4 |
| Kendujhar | 43.2 | 0.000 | 19.4 | 67.0 | 34.8 | 0.000 | 15.8 | 53.8 | 38.7 | 0.000 | 26.4 | 51.0 |
| Mayurbhanj | 14.1 | 0.012 | 3.1 | 25.2 | 29.6 | 0.001 | 12.6 | 46.7 | 22.0 | 0.000 | 12.5 | 31.5 |
| Baleshwar | 29.8 | 0.001 | 11.7 | 47.9 | 16.9 | 0.020 | 2.6 | 31.1 | 23.1 | 0.000 | 11.3 | 34.9 |
| Bhadrak | 25.3 | 0.002 | 9.5 | 41.1 | 34.6 | 0.001 | 14.6 | 54.7 | 30.1 | 0.000 | 16.7 | 43.5 |
| Kendrapara | 22.3 | 0.011 | 5.2 | 39.4 | 19.6 | 0.049 | 0.1 | 39.0 | 20.9 | 0.000 | 11.3 | 30.6 |
| Jagatsinghapur | 17.0 | 0.015 | 3.2 | 30.7 | 50.7 | 0.001 | 19.5 | 81.8 | 35.1 | 0.000 | 17.7 | 52.5 |
| Cuttack | 15.2 | 0.104 | -3.1 | 33.4 | 20.6 | 0.025 | 2.6 | 38.6 | 17.9 | 0.002 | 6.3 | 29.5 |
| Jajapur | 37.7 | 0.000 | 19.8 | 55.7 | 31.7 | 0.001 | 13.6 | 49.8 | 34.5 | 0.000 | 21.1 | 47.8 |
| Dhenkanal | 28.2 | 0.006 | 8.3 | 48.2 | 39.2 | 0.001 | 16.7 | 61.6 | 33.8 | 0.000 | 20.4 | 47.3 |
| Anugul | 51.7 | 0.001 | 22.2 | 81.1 | 35.1 | 0.001 | 14.8 | 55.4 | 43.0 | 0.000 | 25.8 | 60.2 |
| Nayagarh | 44.0 | 0.000 | 19.7 | 68.3 | 39.3 | 0.000 | 23.3 | 55.4 | 41.4 | 0.000 | 25.7 | 57.2 |
| Khordha | 12.6 | 0.007 | 3.4 | 21.9 | 15.3 | 0.002 | 5.8 | 24.8 | 14.0 | 0.000 | 7.2 | 20.8 |
| Puri | 29.2 | 0.003 | 10.0 | 48.3 | 48.6 | 0.000 | 24.7 | 72.5 | 39.4 | 0.000 | 24.9 | 53.9 |
| Ganjam | 12.2 | 0.074 | -1.2 | 25.6 | 21.2 | 0.006 | 6.0 | 36.4 | 17.0 | 0.002 | 6.4 | 27.5 |
| Gajapati | 46.5 | 0.000 | 26.8 | 66.2 | 31.5 | 0.000 | 16.0 | 47.0 | 38.6 | 0.000 | 24.7 | 52.6 |
| Kandhamal | 33.5 | 0.000 | 17.0 | 50.0 | 61.8 | 0.000 | 39.3 | 84.2 | 47.8 | 0.000 | 36.4 | 59.1 |
| Baudh | 66.5 | 0.000 | 36.7 | 96.4 | 52.4 | 0.000 | 31.8 | 73.0 | 59.4 | 0.000 | 42.7 | 76.1 |
| Subarnapur | 19.2 | 0.013 | 4.0 | 34.4 | 48.0 | 0.000 | 25.6 | 70.4 | 34.1 | 0.000 | 23.8 | 44.4 |
| Balangir | 31.9 | 0.000 | 15.7 | 48.1 | 49.9 | 0.000 | 26.5 | 73.2 | 41.1 | 0.000 | 27.5 | 54.6 |
| Nuapada | 42.5 | 0.000 | 23.8 | 61.2 | 45.9 | 0.000 | 26.6 | 65.3 | 44.2 | 0.000 | 29.6 | 58.8 |
| Kalahandi | 27.9 | 0.001 | 12.1 | 43.7 | 31.9 | 0.000 | 15.5 | 48.3 | 30.0 | 0.000 | 16.1 | 43.9 |
| Rayagada | 39.3 | 0.000 | 20.0 | 58.7 | 72.2 | 0.000 | 50.5 | 93.9 | 56.3 | 0.000 | 42.7 | 69.9 |
| Nabarangapur | 36.7 | 0.000 | 21.2 | 52.2 | 54.7 | 0.000 | 38.0 | 71.5 | 45.6 | 0.000 | 33.7 | 57.6 |
| Koraput | 41.9 | 0.000 | 22.2 | 61.6 | 26.1 | 0.001 | 10.9 | 41.4 | 33.8 | 0.000 | 21.3 | 46.3 |
| Malkangiri | 28.6 | 0.000 | 15.4 | 41.7 | 49.8 | 0.000 | 29.4 | 70.3 | 39.8 | 0.000 | 25.3 | 54.4 |
| Korea (koriya) | 41.9 | 0.000 | 24.5 | 59.4 | 50.4 | 0.000 | 35.4 | 65.4 | 46.3 | 0.000 | 33.4 | 59.3 |

| | | | | | | | | | | | | |
|---|---|---|---|---|---|---|---|---|---|---|---|---|
| Surguja | 33.2 | 0.000 | 15.4 | 51.0 | 56.5 | 0.000 | 34.1 | 78.8 | 44.2 | 0.000 | 30.9 | 57.4 |
| Jashpur | 44.1 | 0.000 | 25.6 | 62.7 | 60.0 | 0.000 | 37.5 | 82.5 | 52.3 | 0.000 | 34.3 | 70.4 |
| Raigarh | 30.2 | 0.007 | 8.4 | 52.0 | 47.8 | 0.000 | 21.7 | 73.8 | 39.7 | 0.000 | 25.4 | 54.0 |
| Korba | 52.8 | 0.000 | 39.4 | 66.1 | 60.0 | 0.000 | 46.3 | 73.7 | 56.5 | 0.000 | 43.6 | 69.5 |
| Janjgir - champa | 56.3 | 0.000 | 29.2 | 83.5 | 36.6 | 0.000 | 16.4 | 56.8 | 45.8 | 0.000 | 30.2 | 61.4 |
| Bilaspur | 28.3 | 0.000 | 15.7 | 40.9 | 54.9 | 0.000 | 38.7 | 71.1 | 41.4 | 0.000 | 30.3 | 52.6 |
| Kabirdham | 35.9 | 0.000 | 23.0 | 48.7 | 39.9 | 0.000 | 22.0 | 57.9 | 38.0 | 0.000 | 25.9 | 50.1 |
| Rajnandgaon | 21.6 | 0.001 | 8.3 | 34.9 | 58.4 | 0.000 | 36.9 | 79.9 | 40.5 | 0.000 | 28.0 | 52.9 |
| Durg | 29.7 | 0.000 | 16.3 | 43.0 | 39.7 | 0.000 | 26.3 | 53.1 | 34.9 | 0.000 | 23.3 | 46.5 |
| Raipur | 44.7 | 0.000 | 29.0 | 60.4 | 47.3 | 0.000 | 31.4 | 63.1 | 46.0 | 0.000 | 34.4 | 57.6 |
| Mahasamund | 27.3 | 0.008 | 7.2 | 47.5 | 49.0 | 0.000 | 29.6 | 68.4 | 38.7 | 0.000 | 24.4 | 52.9 |
| Dhamtari | 47.3 | 0.000 | 24.9 | 69.8 | 49.6 | 0.000 | 29.2 | 70.0 | 48.5 | 0.000 | 32.0 | 65.0 |
| Uttar bastar kanker | 38.0 | 0.000 | 17.7 | 58.4 | 61.6 | 0.000 | 36.3 | 87.0 | 50.4 | 0.000 | 31.0 | 69.9 |
| Bastar | 39.4 | 0.000 | 24.2 | 54.6 | 75.8 | 0.000 | 50.2 | 101.3 | 58.0 | 0.000 | 44.4 | 71.5 |
| Narayanpur | 31.2 | 0.000 | 16.3 | 46.1 | 51.7 | 0.000 | 38.2 | 65.1 | 42.0 | 0.000 | 30.7 | 53.4 |
| Dakshin bastar dantewada | 54.1 | 0.000 | 33.5 | 74.7 | 65.3 | 0.000 | 43.8 | 86.9 | 59.9 | 0.000 | 43.5 | 76.2 |
| Bijapur | 25.4 | 0.000 | 12.6 | 38.1 | 52.3 | 0.000 | 34.4 | 70.3 | 39.4 | 0.000 | 28.5 | 50.4 |
| Sheopur | 30.6 | 0.001 | 12.0 | 49.2 | 33.9 | 0.000 | 17.9 | 50.0 | 32.4 | 0.000 | 18.3 | 46.5 |
| Morena | 33.6 | 0.000 | 16.0 | 51.3 | 34.1 | 0.000 | 15.0 | 53.2 | 33.9 | 0.000 | 21.4 | 46.4 |
| Bhind | 29.2 | 0.004 | 9.6 | 48.9 | 45.1 | 0.000 | 29.0 | 61.2 | 37.8 | 0.000 | 24.5 | 51.1 |
| Gwalior | 31.6 | 0.000 | 19.4 | 43.9 | 45.1 | 0.000 | 32.6 | 57.5 | 39.1 | 0.000 | 29.0 | 49.1 |
| Datia | 29.5 | 0.001 | 12.1 | 47.0 | 56.7 | 0.000 | 36.2 | 77.1 | 44.5 | 0.000 | 33.2 | 55.8 |
| Shivpuri | 37.5 | 0.000 | 21.8 | 53.2 | 45.4 | 0.000 | 29.7 | 61.1 | 41.5 | 0.000 | 30.6 | 52.4 |
| Tikamgarh | 51.0 | 0.000 | 27.8 | 74.3 | 36.1 | 0.000 | 18.2 | 54.1 | 43.5 | 0.000 | 29.0 | 58.0 |
| Chhatarpur | 42.1 | 0.000 | 23.6 | 60.7 | 39.0 | 0.000 | 22.7 | 55.2 | 40.5 | 0.000 | 25.4 | 55.6 |
| Panna | 55.2 | 0.000 | 30.8 | 79.7 | 68.8 | 0.000 | 48.6 | 88.9 | 62.6 | 0.000 | 45.6 | 79.5 |
| Sagar | 34.8 | 0.000 | 18.4 | 51.3 | 62.7 | 0.000 | 39.5 | 86.0 | 49.5 | 0.000 | 32.9 | 66.2 |
| Damoh | 38.0 | 0.000 | 20.9 | 55.0 | 47.8 | 0.000 | 28.5 | 67.0 | 43.2 | 0.000 | 31.9 | 54.6 |
| Satna | 39.4 | 0.000 | 20.8 | 57.9 | 41.7 | 0.000 | 20.5 | 62.8 | 40.5 | 0.000 | 27.5 | 53.4 |
| Rewa | 43.2 | 0.000 | 23.2 | 63.1 | 69.9 | 0.000 | 43.0 | 96.7 | 57.4 | 0.000 | 44.6 | 70.2 |
| Umaria | 26.3 | 0.002 | 10.0 | 42.5 | 45.8 | 0.000 | 21.6 | 70.0 | 36.0 | 0.000 | 22.4 | 49.6 |
| Neemuch | 22.3 | 0.001 | 9.4 | 35.3 | 28.4 | 0.001 | 12.2 | 44.7 | 25.5 | 0.000 | 15.1 | 35.9 |
| Mandsaur | 37.2 | 0.000 | 16.9 | 57.4 | 40.4 | 0.000 | 23.5 | 57.4 | 38.9 | 0.000 | 23.2 | 54.6 |
| Ratlam | 27.3 | 0.001 | 10.5 | 44.1 | 25.6 | 0.001 | 11.2 | 40.0 | 26.4 | 0.000 | 18.0 | 34.8 |
| Ujjain | 19.2 | 0.000 | 10.1 | 28.3 | 43.5 | 0.000 | 28.6 | 58.4 | 31.1 | 0.000 | 22.3 | 39.9 |
| Shajapur | 35.3 | 0.000 | 17.3 | 53.4 | 28.9 | 0.000 | 14.8 | 43.0 | 32.1 | 0.000 | 19.4 | 44.7 |
| Dewas | 36.0 | 0.000 | 19.6 | 52.4 | 53.6 | 0.000 | 30.2 | 76.9 | 44.9 | 0.000 | 30.9 | 58.9 |
| Dhar | 38.3 | 0.000 | 22.5 | 54.2 | 48.9 | 0.000 | 26.4 | 71.3 | 43.8 | 0.000 | 31.5 | 56.1 |
| Indore | 14.0 | 0.013 | 3.0 | 25.0 | 13.9 | 0.001 | 5.8 | 21.9 | 13.9 | 0.000 | 7.4 | 20.4 |
| Khargone (west nimar) | 29.0 | 0.000 | 13.7 | 44.2 | 33.4 | 0.000 | 14.6 | 52.2 | 31.3 | 0.000 | 19.8 | 42.8 |
| Barwani | 38.8 | 0.000 | 23.9 | 53.6 | 36.9 | 0.000 | 25.1 | 48.8 | 37.8 | 0.000 | 29.1 | 46.5 |
| Rajgarh | 36.8 | 0.000 | 19.5 | 54.0 | 58.3 | 0.000 | 36.0 | 80.5 | 47.9 | 0.000 | 30.9 | 64.9 |
| Vidisha | 31.6 | 0.000 | 15.0 | 48.2 | 49.9 | 0.000 | 27.6 | 72.3 | 40.7 | 0.000 | 26.9 | 54.6 |
| Bhopal | 27.9 | 0.003 | 9.5 | 46.2 | 34.6 | 0.000 | 17.2 | 51.9 | 31.1 | 0.000 | 18.5 | 43.6 |
| Sehore | 26.3 | 0.002 | 9.3 | 43.3 | 24.0 | 0.002 | 8.6 | 39.5 | 25.1 | 0.000 | 15.8 | 34.4 |
| Raisen | 45.1 | 0.000 | 28.0 | 62.2 | 43.7 | 0.000 | 24.6 | 62.8 | 44.4 | 0.000 | 31.7 | 57.1 |
| Betul | 28.3 | 0.004 | 9.2 | 47.4 | 49.4 | 0.000 | 29.4 | 69.4 | 39.6 | 0.000 | 26.4 | 52.9 |
| Harda | 26.2 | 0.001 | 11.2 | 41.2 | 39.3 | 0.000 | 22.8 | 55.8 | 33.3 | 0.000 | 20.6 | 46.0 |
| Hoshangabad | 36.0 | 0.000 | 21.0 | 51.0 | 31.1 | 0.000 | 18.3 | 44.0 | 33.5 | 0.000 | 23.9 | 43.1 |
| Katni | 35.2 | 0.000 | 16.0 | 54.4 | 46.9 | 0.000 | 28.0 | 65.8 | 41.1 | 0.000 | 27.8 | 54.5 |
| Jabalpur | 42.2 | 0.000 | 24.0 | 60.4 | 43.2 | 0.000 | 26.6 | 59.8 | 42.7 | 0.000 | 32.5 | 52.9 |
| Narsimhapur | 28.7 | 0.003 | 9.8 | 47.6 | 33.4 | 0.002 | 12.4 | 54.4 | 31.3 | 0.000 | 18.3 | 44.3 |
| Dindori | 53.4 | 0.000 | 29.0 | 77.9 | 41.4 | 0.000 | 22.1 | 60.6 | 47.1 | 0.000 | 30.1 | 64.1 |
| Mandla | 37.8 | 0.001 | 15.9 | 59.8 | 47.9 | 0.000 | 27.9 | 67.9 | 42.9 | 0.000 | 28.7 | 57.1 |
| Chhindwara | 38.7 | 0.000 | 23.8 | 53.6 | 40.9 | 0.000 | 18.7 | 63.1 | 39.9 | 0.000 | 24.2 | 55.5 |
| Seoni | 33.5 | 0.001 | 13.5 | 53.6 | 14.9 | 0.017 | 2.6 | 27.3 | 24.1 | 0.000 | 14.8 | 33.4 |
| Balaghat | 48.7 | 0.000 | 25.7 | 71.8 | 60.5 | 0.000 | 34.3 | 86.7 | 54.6 | 0.000 | 36.2 | 73.0 |
| Guna | 45.0 | 0.000 | 30.2 | 59.8 | 61.2 | 0.000 | 42.4 | 79.9 | 53.1 | 0.000 | 40.8 | 65.4 |
| Ashoknagar | 21.6 | 0.000 | 10.7 | 32.5 | 31.6 | 0.000 | 17.5 | 45.6 | 26.7 | 0.000 | 17.1 | 36.4 |
| Shahdol | 48.0 | 0.000 | 26.5 | 69.6 | 29.4 | 0.000 | 13.8 | 44.9 | 38.7 | 0.000 | 24.3 | 53.0 |
| Anuppur | 44.5 | 0.000 | 24.1 | 64.9 | 38.0 | 0.000 | 20.9 | 55.1 | 41.0 | 0.000 | 27.6 | 54.4 |
| Sidhi | 47.1 | 0.000 | 29.0 | 65.1 | 41.8 | 0.000 | 24.5 | 59.1 | 44.3 | 0.000 | 31.9 | 56.8 |
| Singrauli | 26.8 | 0.002 | 10.0 | 43.5 | 38.3 | 0.000 | 23.8 | 52.8 | 32.7 | 0.000 | 23.2 | 42.1 |
| Jhabua | 19.7 | 0.001 | 8.1 | 31.3 | 32.6 | 0.000 | 18.8 | 46.5 | 26.2 | 0.000 | 18.7 | 33.8 |
| Alirajpur | 30.4 | 0.000 | 17.9 | 42.9 | 47.9 | 0.000 | 34.2 | 61.5 | 39.4 | 0.000 | 29.9 | 48.9 |
| Khandwa (east nimar) | 26.4 | 0.003 | 9.0 | 43.7 | 41.1 | 0.000 | 24.9 | 57.2 | 34.4 | 0.000 | 23.7 | 45.0 |
| Burhanpur | 27.1 | 0.000 | 17.2 | 36.9 | 46.6 | 0.000 | 34.4 | 58.8 | 37.6 | 0.000 | 27.9 | 47.3 |
| Kachchh | 27.1 | 0.002 | 10.1 | 44.2 | 45.9 | 0.000 | 26.6 | 65.2 | 36.9 | 0.000 | 21.0 | 52.8 |
| Banaskantha | 34.4 | 0.000 | 16.2 | 52.7 | 55.0 | 0.000 | 31.1 | 78.9 | 45.0 | 0.000 | 30.2 | 59.8 |
| Patan | 24.9 | 0.001 | 10.1 | 39.6 | 42.4 | 0.001 | 17.4 | 67.4 | 34.6 | 0.000 | 19.2 | 50.0 |
| Mahesana | 18.3 | 0.050 | 0.0 | 36.5 | 25.0 | 0.008 | 6.7 | 43.4 | 21.9 | 0.001 | 9.1 | 34.7 |
| Sabarkantha | 27.3 | 0.004 | 8.9 | 45.7 | 30.8 | 0.000 | 14.9 | 46.7 | 29.1 | 0.000 | 15.6 | 42.6 |
| Gandhinagar | 23.6 | 0.035 | 1.7 | 45.4 | 38.3 | 0.001 | 14.7 | 61.9 | 30.9 | 0.000 | 13.6 | 48.3 |
| Ahmadabad | 9.0 | 0.216 | -5.2 | 23.1 | 42.0 | 0.000 | 21.2 | 62.8 | 26.6 | 0.003 | 8.9 | 44.2 |
| Surendranagar | 6.2 | 0.184 | -2.9 | 15.3 | 25.3 | 0.005 | 7.5 | 43.0 | 15.8 | 0.002 | 5.7 | 25.9 |
| Rajkot | 17.6 | 0.033 | 1.4 | 33.7 | 9.7 | 0.076 | -1.0 | 20.5 | 13.2 | 0.017 | 2.4 | 24.0 |
| Jamnagar | 12.1 | 0.048 | 0.1 | 24.1 | 22.5 | 0.006 | 6.5 | 38.5 | 17.6 | 0.003 | 5.9 | 29.4 |
| Porbandar | 14.4 | 0.049 | 0.1 | 28.7 | 10.9 | 0.045 | 0.3 | 21.6 | 12.5 | 0.006 | 3.5 | 21.5 |
| Junagadh | 17.0 | 0.043 | 0.6 | 33.3 | 13.8 | 0.057 | -0.4 | 28.1 | 15.3 | 0.001 | 5.9 | 24.7 |
| Amreli | 9.4 | 0.240 | -6.3 | 25.2 | 7.4 | 0.172 | -3.2 | 18.1 | 8.3 | 0.062 | -0.4 | 16.9 |
| Bhavnagar | 26.8 | 0.017 | 4.8 | 48.9 | 38.4 | 0.000 | 18.2 | 58.6 | 33.0 | 0.000 | 18.6 | 47.4 |

| | | | | | | | | | | | | |
|---|---|---|---|---|---|---|---|---|---|---|---|---|
| Anand | 50.7 | 0.008 | 13.5 | 87.9 | 61.6 | 0.001 | 25.0 | 98.1 | 56.1 | 0.000 | 32.9 | 79.4 |
| Kheda | 27.7 | 0.003 | 9.2 | 46.3 | 55.9 | 0.000 | 27.2 | 84.6 | 42.4 | 0.000 | 26.8 | 58.1 |
| Panchmahal | 24.3 | 0.004 | 8.0 | 40.7 | 35.8 | 0.000 | 18.1 | 53.5 | 30.3 | 0.000 | 17.6 | 43.0 |
| Dohad | 39.6 | 0.000 | 20.8 | 58.3 | 58.1 | 0.000 | 32.8 | 83.4 | 49.1 | 0.000 | 34.8 | 63.4 |
| Vadodara | 14.9 | 0.042 | 0.6 | 29.2 | 18.0 | 0.018 | 3.1 | 32.9 | 16.5 | 0.004 | 5.3 | 27.6 |
| Narmada | 35.4 | 0.000 | 16.5 | 54.3 | 33.5 | 0.001 | 13.2 | 53.7 | 34.4 | 0.000 | 20.8 | 48.1 |
| Bharuch | 23.5 | 0.007 | 6.5 | 40.6 | 51.4 | 0.000 | 23.3 | 79.5 | 37.2 | 0.000 | 19.8 | 54.6 |
| The dangs | 18.1 | 0.001 | 7.2 | 29.1 | 22.2 | 0.001 | 8.8 | 35.5 | 20.3 | 0.000 | 9.9 | 30.6 |
| Navsari | 19.7 | 0.017 | 3.6 | 35.8 | 31.6 | 0.008 | 8.1 | 55.1 | 25.8 | 0.001 | 10.4 | 41.1 |
| Valsad | 11.6 | 0.077 | -1.2 | 24.5 | 35.5 | 0.003 | 11.7 | 59.2 | 23.6 | 0.000 | 11.6 | 35.5 |
| Surat | 13.8 | 0.079 | -1.6 | 29.3 | 6.4 | 0.198 | -3.3 | 16.1 | 9.8 | 0.028 | 1.0 | 18.6 |
| Tapi | 28.1 | 0.008 | 7.2 | 49.0 | 7.4 | 0.167 | -3.1 | 17.8 | 17.8 | 0.006 | 5.0 | 30.5 |
| Diu | 12.5 | 0.115 | -3.0 | 28.0 | 9.0 | 0.086 | -1.3 | 19.3 | 10.6 | 0.043 | 0.3 | 20.8 |
| Daman | 17.4 | 0.019 | 2.8 | 32.0 | 34.5 | 0.010 | 8.2 | 60.8 | 26.5 | 0.000 | 11.9 | 41.2 |
| Dadra & nagar haveli | 10.3 | 0.119 | -2.6 | 23.2 | 21.6 | 0.005 | 6.4 | 36.9 | 16.2 | 0.000 | 8.1 | 24.3 |
| Nandurbar | 15.3 | 0.010 | 3.6 | 26.9 | 23.7 | 0.004 | 7.4 | 40.0 | 19.4 | 0.001 | 7.8 | 31.1 |
| Dhule | 11.1 | 0.006 | 3.2 | 19.0 | 7.9 | 0.109 | -1.8 | 17.6 | 9.4 | 0.010 | 2.2 | 16.7 |
| Jalgaon | 26.5 | 0.013 | 5.5 | 47.5 | 28.2 | 0.004 | 9.1 | 47.4 | 27.5 | 0.000 | 12.2 | 42.7 |
| Buldana | 22.4 | 0.007 | 6.2 | 38.5 | 20.5 | 0.005 | 6.1 | 34.9 | 21.4 | 0.002 | 8.2 | 34.6 |
| Akola | 22.5 | 0.004 | 7.0 | 38.0 | 31.1 | 0.002 | 11.8 | 50.4 | 27.2 | 0.000 | 14.6 | 39.7 |
| Washim | 16.3 | 0.018 | 2.8 | 29.8 | 11.2 | 0.082 | -1.4 | 23.9 | 13.5 | 0.005 | 4.1 | 23.0 |
| Amravati | 4.6 | 0.215 | -2.7 | 11.9 | 16.4 | 0.082 | -2.1 | 35.0 | 10.5 | 0.024 | 1.4 | 19.6 |
| Wardha | 2.7 | 0.334 | -2.8 | 8.2 | 42.4 | 0.004 | 13.2 | 71.7 | 22.5 | 0.000 | 10.8 | 34.1 |
| Nagpur | 22.9 | 0.084 | -3.1 | 48.9 | 25.0 | 0.162 | -10.0 | 60.0 | 24.0 | 0.012 | 5.3 | 42.6 |
| Bhandara | 19.7 | 0.035 | 1.4 | 38.0 | 46.5 | 0.000 | 24.3 | 68.7 | 33.1 | 0.000 | 14.8 | 51.3 |
| Gondiya | 21.9 | 0.022 | 3.1 | 40.6 | 18.7 | 0.041 | 0.7 | 36.6 | 20.4 | 0.001 | 7.9 | 32.8 |
| Gadchiroli | 12.6 | 0.075 | -1.3 | 26.4 | 36.7 | 0.004 | 12.0 | 61.4 | 25.2 | 0.003 | 8.8 | 41.5 |
| Chandrapur | 10.8 | 0.193 | -5.5 | 27.0 | 29.4 | 0.027 | 3.3 | 55.5 | 19.9 | 0.003 | 6.9 | 32.9 |
| Yavatmal | 8.1 | 0.203 | -4.4 | 20.5 | 25.3 | 0.005 | 7.4 | 43.1 | 17.4 | 0.008 | 4.6 | 30.1 |
| Nanded | 30.8 | 0.001 | 13.0 | 48.6 | 28.0 | 0.002 | 10.6 | 45.4 | 29.3 | 0.000 | 18.9 | 39.8 |
| Hingoli | 36.7 | 0.001 | 15.5 | 58.0 | 33.8 | 0.001 | 13.8 | 53.9 | 35.3 | 0.000 | 22.1 | 48.5 |
| Parbhani | 11.6 | 0.013 | 2.4 | 20.7 | 27.2 | 0.001 | 11.7 | 42.7 | 19.9 | 0.000 | 12.0 | 27.8 |
| Jalna | 10.9 | 0.037 | 0.7 | 21.2 | 25.5 | 0.001 | 9.8 | 41.2 | 18.5 | 0.000 | 9.5 | 27.5 |
| Aurangabad | 6.0 | 0.176 | -2.7 | 14.6 | 3.6 | 0.208 | -2.0 | 9.2 | 4.8 | 0.080 | -0.6 | 10.2 |
| Nashik | 13.4 | 0.040 | 0.6 | 26.1 | 25.9 | 0.001 | 10.4 | 41.4 | 20.0 | 0.000 | 12.3 | 27.8 |
| Thane | 4.6 | 0.281 | -3.8 | 13.1 | 16.1 | 0.058 | -0.5 | 32.7 | 10.6 | 0.013 | 2.2 | 19.0 |
| Mumbai suburban | 5.0 | 0.348 | -5.4 | 15.5 | 31.7 | 0.093 | -5.3 | 68.7 | 19.3 | 0.027 | 2.2 | 36.4 |
| Mumbai | 13.8 | 0.114 | -3.3 | 31.0 | 14.6 | 0.119 | -3.8 | 32.9 | 14.2 | 0.037 | 0.9 | 27.6 |
| Raigarh | 24.3 | 0.072 | -2.2 | 50.9 | 15.9 | 0.100 | -3.1 | 34.9 | 19.9 | 0.009 | 5.0 | 34.8 |
| Pune | 19.2 | 0.102 | -3.8 | 42.1 | 19.6 | 0.031 | 1.8 | 37.3 | 19.4 | 0.004 | 6.1 | 32.7 |
| Ahmadnagar | 3.4 | 0.340 | -3.6 | 10.4 | 2.7 | 0.317 | -2.6 | 8.1 | 3.0 | 0.203 | -1.6 | 7.7 |
| Bid | 10.3 | 0.047 | 0.1 | 20.4 | 14.9 | 0.021 | 2.3 | 27.6 | 12.8 | 0.003 | 4.4 | 21.2 |
| Latur | 11.8 | 0.039 | 0.6 | 22.9 | 20.7 | 0.002 | 7.7 | 33.6 | 16.6 | 0.001 | 6.9 | 26.3 |
| Osmanabad | 13.4 | 0.120 | -3.5 | 30.3 | 29.2 | 0.002 | 10.5 | 47.9 | 21.9 | 0.000 | 10.4 | 33.3 |
| Solapur | 15.5 | 0.127 | -4.4 | 35.3 | 28.4 | 0.003 | 9.4 | 47.3 | 22.8 | 0.008 | 5.9 | 39.7 |
| Satara | 17.8 | 0.191 | -8.9 | 44.4 | 16.8 | 0.034 | 1.3 | 32.3 | 17.3 | 0.016 | 3.2 | 31.3 |
| Ratnagiri | 12.8 | 0.076 | -1.3 | 27.0 | 30.7 | 0.002 | 11.4 | 50.1 | 22.0 | 0.001 | 9.2 | 34.9 |
| Sindhudurg | 13.7 | 0.154 | -5.1 | 32.5 | 29.6 | 0.031 | 2.6 | 56.6 | 22.3 | 0.002 | 7.9 | 36.8 |
| Kolhapur | 5.3 | 0.224 | -3.3 | 13.9 | 10.5 | 0.081 | -1.3 | 22.3 | 8.3 | 0.058 | -0.3 | 16.9 |
| Sangli | 16.9 | 0.097 | -3.1 | 37.0 | 22.7 | 0.012 | 5.1 | 40.3 | 20.1 | 0.003 | 7.0 | 33.2 |
| Adilabad | 46.5 | 0.003 | 15.3 | 77.7 | 35.2 | 0.002 | 12.6 | 57.9 | 40.5 | 0.000 | 22.3 | 58.7 |
| Nizamabad | 43.7 | 0.000 | 19.2 | 68.1 | 51.3 | 0.000 | 24.0 | 78.5 | 47.9 | 0.000 | 29.6 | 66.1 |
| Karimnagar | 25.2 | 0.021 | 3.7 | 46.7 | 29.7 | 0.005 | 8.9 | 50.5 | 27.4 | 0.001 | 10.8 | 44.1 |
| Medak | 26.6 | 0.011 | 6.1 | 47.2 | 25.3 | 0.011 | 5.7 | 45.0 | 26.0 | 0.000 | 13.2 | 38.8 |
| Hyderabad | 16.6 | 0.080 | -2.0 | 35.2 | 20.1 | 0.094 | -3.4 | 43.7 | 18.5 | 0.014 | 3.7 | 33.3 |
| Rangareddy | 6.0 | 0.223 | -3.7 | 15.7 | 38.4 | 0.000 | 19.4 | 57.4 | 23.2 | 0.001 | 10.1 | 36.3 |
| Mahbubnagar | 37.5 | 0.000 | 21.4 | 53.7 | 50.3 | 0.000 | 24.0 | 76.5 | 44.0 | 0.000 | 28.8 | 59.3 |
| Nalgonda | 17.4 | 0.052 | -0.1 | 34.9 | 29.9 | 0.003 | 10.3 | 49.5 | 24.1 | 0.001 | 9.6 | 38.6 |
| Warangal | 27.8 | 0.016 | 5.2 | 50.5 | 9.3 | 0.148 | -3.3 | 21.9 | 18.4 | 0.004 | 5.9 | 30.9 |
| Khammam | 25.1 | 0.030 | 2.5 | 47.8 | 33.7 | 0.007 | 9.4 | 58.1 | 29.5 | 0.000 | 14.5 | 44.6 |
| Srikakulam | 43.4 | 0.000 | 22.5 | 64.3 | 45.8 | 0.000 | 21.6 | 69.9 | 44.7 | 0.000 | 26.1 | 63.3 |
| Vizianagaram | 25.4 | 0.016 | 4.7 | 46.1 | 28.1 | 0.002 | 10.4 | 45.9 | 26.8 | 0.000 | 14.9 | 38.8 |
| Visakhapatnam | 5.7 | 0.234 | -3.7 | 15.1 | 37.7 | 0.029 | 3.9 | 71.6 | 21.8 | 0.001 | 9.0 | 34.6 |
| East godavari | 33.4 | 0.009 | 8.5 | 58.4 | 28.1 | 0.026 | 3.4 | 52.9 | 30.7 | 0.001 | 11.8 | 49.7 |
| West godavari | 11.6 | 0.120 | -3.0 | 26.2 | 14.2 | 0.083 | -1.8 | 30.3 | 13.0 | 0.033 | 1.0 | 24.9 |
| Krishna | 37.9 | 0.007 | 10.5 | 65.3 | 13.0 | 0.117 | -3.2 | 29.2 | 24.8 | 0.007 | 6.9 | 42.7 |
| Guntur | 28.4 | 0.022 | 4.0 | 52.9 | 30.7 | 0.033 | 2.5 | 58.9 | 29.7 | 0.000 | 13.0 | 46.4 |
| Prakasam | 43.2 | 0.002 | 15.8 | 70.7 | 46.7 | 0.000 | 23.9 | 69.5 | 45.0 | 0.000 | 27.8 | 62.3 |
| Sri potti sriramulu nellore | 12.6 | 0.097 | -2.3 | 27.5 | 27.9 | 0.006 | 8.0 | 47.7 | 20.0 | 0.003 | 6.8 | 33.2 |
| Y.s.r. | 12.5 | 0.083 | -1.6 | 26.7 | 39.5 | 0.001 | 15.4 | 63.5 | 27.9 | 0.000 | 12.9 | 42.8 |
| Kurnool | 28.4 | 0.009 | 7.1 | 49.7 | 38.5 | 0.000 | 17.3 | 59.7 | 33.7 | 0.000 | 20.8 | 46.7 |
| Anantapur | 17.9 | 0.006 | 5.2 | 30.7 | 50.4 | 0.007 | 14.1 | 86.7 | 34.2 | 0.000 | 17.0 | 51.5 |
| Chittoor | 16.6 | 0.068 | -1.2 | 34.5 | 35.9 | 0.009 | 9.0 | 62.7 | 26.0 | 0.000 | 12.3 | 39.7 |
| Belgaum | 17.5 | 0.011 | 4.0 | 31.1 | 19.3 | 0.015 | 3.7 | 34.9 | 18.4 | 0.000 | 8.4 | 28.5 |
| Bagalkot | 22.5 | 0.000 | 10.9 | 34.2 | 11.4 | 0.069 | -0.9 | 23.7 | 16.4 | 0.000 | 7.4 | 25.4 |
| Bijapur | 15.0 | 0.012 | 3.3 | 26.7 | 11.8 | 0.030 | 1.1 | 22.4 | 13.4 | 0.001 | 5.7 | 21.0 |
| Bidar | 12.1 | 0.033 | 1.0 | 23.3 | 27.9 | 0.001 | 11.3 | 44.4 | 19.8 | 0.000 | 11.3 | 28.3 |
| Raichur | 29.9 | 0.001 | 12.6 | 47.2 | 44.0 | 0.000 | 23.6 | 64.4 | 37.2 | 0.000 | 24.8 | 49.6 |
| Koppal | 9.2 | 0.105 | -1.9 | 20.3 | 16.9 | 0.006 | 4.8 | 29.0 | 13.1 | 0.004 | 4.3 | 21.9 |
| Gadag | 10.8 | 0.239 | -7.2 | 28.8 | 19.8 | 0.115 | -4.8 | 44.4 | 15.7 | 0.030 | 1.5 | 29.9 |
| Dharwad | 33.5 | 0.001 | 14.0 | 53.1 | 23.6 | 0.011 | 5.5 | 41.8 | 28.5 | 0.000 | 12.5 | 44.5 |

| | | | | | | | | | | | | |
|---|---|---|---|---|---|---|---|---|---|---|---|---|
| Uttara kannada | 12.0 | 0.070 | -1.0 | 25.0 | 17.8 | 0.061 | -0.8 | 36.4 | 15.0 | 0.012 | 3.3 | 26.6 |
| Haveri | 5.8 | 0.289 | -5.0 | 16.6 | 17.1 | 0.034 | 1.3 | 33.0 | 11.6 | 0.056 | -0.3 | 23.6 |
| Bellary | 28.1 | 0.002 | 10.1 | 46.0 | 23.1 | 0.003 | 7.9 | 38.3 | 25.4 | 0.000 | 12.7 | 38.0 |
| Chitradurga | 17.8 | 0.033 | 1.4 | 34.3 | 22.4 | 0.018 | 3.8 | 40.9 | 20.3 | 0.001 | 8.6 | 32.0 |
| Davanagere | 15.9 | 0.065 | -1.0 | 32.7 | 47.2 | 0.001 | 20.1 | 74.2 | 31.8 | 0.000 | 16.2 | 47.3 |
| Shimoga | 16.1 | 0.049 | 0.1 | 32.2 | 41.0 | 0.005 | 12.6 | 69.3 | 28.5 | 0.000 | 12.5 | 44.6 |
| Udupi | 12.5 | 0.151 | -4.6 | 29.5 | 14.1 | 0.121 | -3.7 | 31.8 | 13.3 | 0.016 | 2.5 | 24.2 |
| Chikmagalur | 18.2 | 0.126 | -5.1 | 41.6 | 6.4 | 0.277 | -5.2 | 18.1 | 12.9 | 0.041 | 0.5 | 25.2 |
| Tumkur | 43.2 | 0.000 | 19.0 | 67.4 | 35.6 | 0.002 | 13.5 | 57.6 | 39.5 | 0.000 | 24.4 | 54.5 |
| Bangalore | 3.8 | 0.449 | -6.1 | 13.8 | 7.7 | 0.086 | -1.1 | 16.5 | 5.9 | 0.058 | -0.2 | 12.1 |
| Mandya | 24.8 | 0.013 | 5.3 | 44.3 | 15.8 | 0.156 | -6.0 | 37.6 | 20.1 | 0.002 | 7.5 | 32.7 |
| Hassan | 17.6 | 0.054 | -0.3 | 35.4 | 31.9 | 0.022 | 4.6 | 59.2 | 24.7 | 0.000 | 10.9 | 38.6 |
| Dakshina kannada | 17.6 | 0.027 | 2.0 | 33.3 | 19.6 | 0.042 | 0.7 | 38.5 | 18.6 | 0.013 | 3.9 | 33.3 |
| Kodagu | 0.0 | 0.000 | 0.0 | 0.0 | 21.7 | 0.021 | 3.3 | 40.1 | 10.7 | 0.021 | 1.6 | 19.8 |
| Mysore | 14.0 | 0.071 | -1.2 | 29.2 | 28.0 | 0.015 | 5.5 | 50.5 | 21.3 | 0.002 | 7.9 | 34.8 |
| Chamarajanagar | 24.2 | 0.008 | 6.2 | 42.2 | 42.4 | 0.001 | 16.9 | 67.9 | 33.0 | 0.000 | 16.2 | 49.9 |
| Gulbarga | 0.0 | 0.000 | 0.0 | 0.0 | 24.2 | 0.003 | 8.3 | 40.1 | 12.4 | 0.001 | 4.8 | 20.0 |
| Yadgir | 15.7 | 0.017 | 2.8 | 28.7 | 12.1 | 0.014 | 2.5 | 21.6 | 13.8 | 0.001 | 6.0 | 21.6 |
| Kolar | 20.6 | 0.025 | 2.6 | 38.7 | 25.9 | 0.004 | 8.4 | 43.3 | 23.5 | 0.000 | 10.5 | 36.4 |
| Chikkaballapura | 9.5 | 0.233 | -6.1 | 25.2 | 18.9 | 0.038 | 1.1 | 36.8 | 14.3 | 0.012 | 3.2 | 25.4 |
| Bangalore rural | 17.9 | 0.020 | 2.8 | 33.1 | 30.6 | 0.011 | 7.2 | 54.0 | 24.1 | 0.000 | 10.9 | 37.3 |
| Ramanagara | 14.3 | 0.132 | -4.3 | 32.8 | 27.8 | 0.021 | 4.2 | 51.3 | 21.0 | 0.001 | 8.2 | 33.8 |
| North goa | 9.4 | 0.108 | -2.1 | 21.0 | 11.0 | 0.073 | -1.0 | 23.0 | 10.3 | 0.011 | 2.3 | 18.3 |
| South goa | 19.5 | 0.075 | -2.0 | 41.0 | 12.5 | 0.109 | -2.8 | 27.8 | 16.0 | 0.027 | 1.8 | 30.2 |
| Lakshadweep | 27.3 | 0.005 | 8.0 | 46.5 | 8.0 | 0.056 | -0.2 | 16.2 | 17.3 | 0.002 | 6.5 | 28.1 |
| Kasaragod | 0.0 | 0.000 | 0.0 | 0.0 | 3.7 | 0.431 | -5.5 | 13.0 | 2.0 | 0.364 | -2.3 | 6.3 |
| Kannur | 0.0 | 0.000 | 0.0 | 0.0 | 6.9 | 0.241 | -4.6 | 18.4 | 3.4 | 0.133 | -1.0 | 7.9 |
| Wayanad | 9.0 | 0.045 | 0.2 | 17.8 | 14.8 | 0.095 | -2.6 | 32.1 | 11.8 | 0.012 | 2.6 | 20.9 |
| Kozhikode | 5.1 | 0.304 | -4.6 | 14.9 | 8.6 | 0.219 | -5.1 | 22.4 | 7.0 | 0.114 | -1.7 | 15.7 |
| Malappuram | 3.3 | 0.347 | -3.6 | 10.1 | 2.9 | 0.351 | -3.2 | 8.9 | 3.1 | 0.235 | -2.0 | 8.1 |
| Palakkad | 0.0 | 0.000 | 0.0 | 0.0 | 3.5 | 0.199 | -1.9 | 9.0 | 1.9 | 0.193 | -1.0 | 4.8 |
| Thrissur | 0.0 | 0.000 | 0.0 | 0.0 | 0.0 | 0.000 | 0.0 | 0.0 | 0.0 | 0.000 | 0.0 | 0.0 |
| Ernakulam | 0.0 | 0.000 | 0.0 | 0.0 | 3.4 | 0.306 | -3.1 | 9.8 | 1.6 | 0.279 | -1.3 | 4.5 |
| Idukki | 0.0 | 0.000 | 0.0 | 0.0 | 2.8 | 0.263 | -2.1 | 7.8 | 1.4 | 0.422 | -2.0 | 4.8 |
| Kottayam | 5.8 | 0.344 | -6.2 | 17.9 | 6.0 | 0.326 | -6.0 | 18.0 | 5.9 | 0.212 | -3.4 | 15.2 |
| Alappuzha | 31.9 | 0.078 | -3.5 | 67.3 | 5.9 | 0.300 | -5.3 | 17.2 | 19.1 | 0.007 | 5.2 | 33.1 |
| Pathanamthitta | 7.2 | 0.325 | -7.2 | 21.6 | 6.9 | 0.335 | -7.1 | 20.9 | 7.0 | 0.155 | -2.7 | 16.8 |
| Kollam | 4.8 | 0.413 | -6.8 | 16.5 | 13.7 | 0.185 | -6.6 | 34.0 | 9.7 | 0.107 | -2.1 | 21.4 |
| Thiruvananthapuram | 0.0 | 0.000 | 0.0 | 0.0 | 14.5 | 0.047 | 0.2 | 28.8 | 7.1 | 0.050 | 0.0 | 14.2 |
| Thiruvallur | 16.8 | 0.028 | 1.8 | 31.8 | 26.2 | 0.033 | 2.2 | 50.3 | 22.0 | 0.001 | 9.1 | 34.9 |
| Chennai | 1.7 | 0.145 | -0.6 | 4.1 | 0.0 | 0.000 | 0.0 | 0.0 | 1.0 | 0.139 | -0.3 | 2.2 |
| Kancheepuram | 14.2 | 0.083 | -1.9 | 30.4 | 2.7 | 0.341 | -2.8 | 8.2 | 8.4 | 0.063 | -0.5 | 17.2 |
| Vellore | 18.2 | 0.032 | 1.6 | 34.8 | 7.8 | 0.077 | -0.9 | 16.4 | 13.0 | 0.016 | 2.4 | 23.6 |
| Tiruvannamalai | 15.4 | 0.068 | -1.1 | 31.9 | 22.8 | 0.012 | 5.0 | 40.7 | 19.3 | 0.000 | 8.6 | 30.0 |
| Viluppuram | 9.6 | 0.060 | -0.4 | 19.7 | 19.1 | 0.053 | -0.3 | 38.4 | 14.7 | 0.002 | 5.4 | 24.0 |
| Salem | 0.0 | 0.000 | 0.0 | 0.0 | 22.0 | 0.010 | 5.2 | 38.7 | 11.4 | 0.019 | 1.9 | 20.8 |
| Namakkal | 3.3 | 0.386 | -4.2 | 10.8 | 20.2 | 0.053 | -0.2 | 40.7 | 11.8 | 0.030 | 1.1 | 22.5 |
| Erode | 3.5 | 0.325 | -3.4 | 10.4 | 0.0 | 0.000 | 0.0 | 0.0 | 1.7 | 0.325 | -1.7 | 5.1 |
| The nilgiris | 5.7 | 0.196 | -2.9 | 14.3 | 3.1 | 0.319 | -3.0 | 9.2 | 4.4 | 0.056 | -0.1 | 9.0 |
| Dindigul | 5.1 | 0.346 | -5.5 | 15.6 | 22.2 | 0.043 | 0.7 | 43.8 | 14.1 | 0.028 | 1.5 | 26.7 |
| Karur | 16.4 | 0.065 | -1.0 | 33.8 | 7.9 | 0.231 | -5.0 | 20.7 | 12.0 | 0.031 | 1.1 | 22.8 |
| Tiruchirappalli | 16.3 | 0.054 | -0.3 | 33.0 | 16.8 | 0.046 | 0.3 | 33.2 | 16.6 | 0.005 | 5.1 | 28.0 |
| Perambalur | 3.7 | 0.272 | -2.9 | 10.4 | 29.5 | 0.042 | 1.1 | 57.9 | 17.9 | 0.001 | 7.3 | 28.5 |
| Ariyalur | 16.3 | 0.082 | -2.1 | 34.8 | 17.6 | 0.079 | -2.0 | 37.1 | 17.0 | 0.007 | 4.7 | 29.3 |
| Cuddalore | 5.6 | 0.353 | -6.3 | 17.6 | 21.4 | 0.014 | 4.3 | 38.4 | 14.7 | 0.007 | 4.0 | 25.4 |
| Nagapattinam | 22.7 | 0.011 | 5.2 | 40.1 | 12.5 | 0.055 | -0.3 | 25.3 | 17.5 | 0.000 | 7.8 | 27.3 |
| Thiruvarur | 16.9 | 0.033 | 1.3 | 32.5 | 23.3 | 0.045 | 0.5 | 46.0 | 20.2 | 0.010 | 4.9 | 35.5 |
| Thanjavur | 23.2 | 0.073 | -2.2 | 48.5 | 12.6 | 0.114 | -3.0 | 28.2 | 17.4 | 0.003 | 5.9 | 28.9 |
| Pudukkottai | 7.9 | 0.123 | -2.1 | 18.0 | 12.9 | 0.040 | 0.6 | 25.1 | 10.3 | 0.036 | 0.7 | 20.0 |
| Sivaganga | 34.4 | 0.003 | 12.1 | 56.7 | 9.9 | 0.283 | -8.2 | 27.9 | 23.1 | 0.000 | 11.2 | 35.0 |
| Madurai | 11.5 | 0.087 | -1.7 | 24.7 | 17.9 | 0.035 | 1.3 | 34.6 | 14.9 | 0.003 | 5.0 | 24.7 |
| Theni | 3.2 | 0.388 | -4.1 | 10.5 | 22.0 | 0.068 | -1.6 | 45.5 | 12.9 | 0.045 | 0.3 | 25.5 |
| Virudhunagar | 14.8 | 0.071 | -1.2 | 30.9 | 14.8 | 0.090 | -2.3 | 31.9 | 14.8 | 0.023 | 2.0 | 27.6 |
| Ramanathapuram | 2.1 | 0.281 | -1.7 | 5.8 | 7.2 | 0.092 | -1.2 | 15.5 | 4.8 | 0.043 | 0.2 | 9.5 |
| Thoothukkudi | 8.8 | 0.238 | -5.8 | 23.4 | 4.5 | 0.376 | -5.5 | 14.4 | 6.7 | 0.159 | -2.6 | 16.1 |
| Tirunelveli | 10.9 | 0.218 | -6.4 | 28.3 | 36.0 | 0.011 | 8.3 | 63.7 | 23.3 | 0.002 | 8.4 | 38.2 |
| Kanniyakumari | 13.5 | 0.178 | -6.1 | 33.1 | 10.6 | 0.101 | -2.1 | 23.4 | 12.0 | 0.027 | 1.3 | 22.6 |
| Dharmapuri | 7.1 | 0.218 | -4.2 | 18.4 | 15.3 | 0.025 | 1.9 | 28.8 | 11.3 | 0.037 | 0.7 | 22.0 |
| Krishnagiri | 19.9 | 0.006 | 5.7 | 34.1 | 27.1 | 0.001 | 11.2 | 43.0 | 23.8 | 0.001 | 9.7 | 37.9 |
| Coimbatore | 10.7 | 0.243 | -7.2 | 28.6 | 19.0 | 0.031 | 1.7 | 36.2 | 15.0 | 0.007 | 4.1 | 26.0 |
| Tiruppur | 9.2 | 0.113 | -2.2 | 20.5 | 21.9 | 0.055 | -0.4 | 44.3 | 15.5 | 0.005 | 4.6 | 26.5 |
| Yanam | 10.3 | 0.084 | -1.4 | 22.0 | 13.3 | 0.045 | 0.3 | 26.3 | 11.9 | 0.005 | 3.5 | 20.2 |
| Puducherry | 2.2 | 0.254 | -1.6 | 6.1 | 18.7 | 0.014 | 3.8 | 33.6 | 11.0 | 0.025 | 1.4 | 20.6 |
| Mahe | 3.8 | 0.251 | -2.7 | 10.3 | 6.2 | 0.167 | -2.6 | 14.9 | 5.0 | 0.081 | -0.6 | 10.5 |
| Karaikal | 18.1 | 0.025 | 2.2 | 33.9 | 18.4 | 0.030 | 1.8 | 35.0 | 18.3 | 0.001 | 7.1 | 29.5 |
| Nicobars | 10.6 | 0.051 | 0.0 | 21.2 | 2.0 | 0.251 | -1.4 | 5.5 | 6.2 | 0.076 | -0.6 | 13.1 |
| North & middle andaman | 13.7 | 0.061 | -0.7 | 28.1 | 15.8 | 0.048 | 0.1 | 31.5 | 14.8 | 0.012 | 3.3 | 26.3 |
| South andaman | 5.1 | 0.332 | -5.2 | 15.3 | 8.2 | 0.119 | -2.1 | 18.5 | 6.6 | 0.057 | -0.2 | 13.5 |
| **India** | **26.6** | **0.000** | **25.8** | **27.5** | **34.0** | **0.000** | **33.1** | **34.9** | **30.5** | **0.000** | **29.9** | **31.1** |
