# Supplementary material for: Neonatal and under-five mortality rate in Indian districts with reference to Sustainable Development Goal 3: An analysis of the National Family Health Survey of India (NFHS), 2015–2016
Source: PLoS One. 2018 Jul 30;13(7):e0201125. doi: 10.1371/journal.pone.0201125 (PMC6066210; doi:10.1371/journal.pone.0201125)
Supplement: S2 Table — (PDF) [file pone.0201125.s004.pdf]

S2 Table. Estimated Districtwise Under-five mortality rate for ten-years periods preceding the survey, by gender, India, 2015-16.

District	Female				Male				Total			
	U5MR	P value	95% CI		U5MR	P value	95% CI		U5MR	P value	95% CI	
			Lower	Upper			Lower	Upper			Lower	Upper
Kupwara	49.6	0.000	31.1	68.2	67.6	0.000	41.8	93.4	59.0	0.000	43.1	74.9
Badgam	36.6	0.001	14.8	58.4	29.6	0.001	11.9	47.2	33.1	0.000	18.7	47.4
Leh	45.5	0.014	9.0	81.9	31.5	0.011	7.1	56.0	38.1	0.000	20.1	56.0
Kargil	40.8	0.007	11.4	70.2	66.6	0.000	43.9	89.3	54.5	0.000	37.4	71.6
Punch	34.2	0.000	19.3	49.2	41.4	0.000	26.5	56.2	38.1	0.000	25.9	50.4
Rajouri	32.3	0.000	15.6	48.9	41.6	0.000	20.6	62.6	37.1	0.000	24.4	49.8
Kathua	38.5	0.001	15.9	61.1	25.1	0.002	9.0	41.2	31.3	0.000	18.9	43.7
Baramula	34.5	0.000	16.8	52.1	52.1	0.000	28.1	76.1	43.7	0.000	28.5	58.9
Bandipore	42.8	0.000	21.2	64.3	56.0	0.000	32.8	79.3	49.2	0.000	31.2	67.1
Srinagar	18.9	0.023	2.6	35.2	36.1	0.014	7.3	64.9	27.9	0.000	15.0	40.8
Ganderbal	30.4	0.000	16.0	44.9	56.0	0.000	29.4	82.5	42.5	0.000	26.9	58.0
Pulwama	19.1	0.010	4.6	33.5	68.8	0.000	38.6	99.0	44.5	0.000	29.1	59.8
Shupiyan	32.2	0.004	10.2	54.2	32.6	0.000	17.4	47.8	32.7	0.000	17.8	47.5
Anantnag	45.9	0.000	21.8	69.9	41.7	0.000	21.4	62.0	43.7	0.000	28.2	59.2
Kulgam	32.3	0.002	11.8	52.8	75.2	0.000	54.4	96.1	54.6	0.000	35.7	73.6
Doda	37.5	0.000	18.4	56.6	37.2	0.000	18.4	56.0	37.4	0.000	24.3	50.5
Ramban	23.4	0.002	8.5	38.4	35.8	0.000	19.6	52.1	29.9	0.000	19.3	40.5
Kishtwar	44.6	0.000	23.8	65.4	46.1	0.000	27.5	64.6	45.4	0.000	30.4	60.5
Udhampur	30.6	0.001	12.7	48.6	19.0	0.003	6.4	31.5	24.6	0.000	12.6	36.6
Reasi	21.1	0.000	10.6	31.7	25.9	0.000	14.0	37.8	23.6	0.000	13.1	34.0
Jammu	23.0	0.017	4.1	41.8	19.4	0.036	1.3	37.6	21.0	0.000	10.0	32.1
Samba	13.9	0.033	1.2	26.7	19.7	0.015	3.9	35.5	17.1	0.002	6.2	28.0
Chamba	56.3	0.000	31.6	81.0	85.7	0.000	61.9	109.6	71.4	0.000	51.4	91.4
Kangra	14.3	0.062	-0.7	29.2	40.2	0.001	16.4	64.0	28.4	0.000	13.4	43.4
Lahul and spiti	43.9	0.017	7.7	80.2	37.1	0.014	7.5	66.6	40.9	0.000	20.9	60.9
Kullu	36.1	0.009	9.1	63.1	25.3	0.010	6.1	44.5	30.6	0.000	14.3	46.9
Mandi	68.9	0.000	42.8	94.9	25.5	0.048	0.2	50.8	47.4	0.000	29.2	65.5
Hamirpur	33.7	0.006	9.4	58.0	28.0	0.006	7.8	48.1	30.8	0.000	13.8	47.8
Una	35.3	0.003	11.6	58.9	42.5	0.001	18.6	66.4	39.1	0.000	20.7	57.4
Bilaspur	24.9	0.008	6.6	43.3	16.8	0.050	0.0	33.5	20.7	0.000	10.3	31.1
Solan	28.9	0.008	7.6	50.3	29.8	0.009	7.4	52.2	29.4	0.000	15.2	43.5
Sirmaur	34.1	0.001	14.1	54.1	37.7	0.001	15.3	60.2	35.9	0.000	23.0	48.8
Shimla	23.4	0.036	1.5	45.4	68.8	0.000	33.0	104.6	46.0	0.000	25.8	66.2
Kinnaur	82.4	0.001	35.2	129.6	83.0	0.000	42.6	123.3	82.7	0.000	54.4	111.0
Gurdaspur	27.8	0.007	7.6	47.9	28.4	0.008	7.4	49.4	28.2	0.000	17.1	39.2
Kapurthala	34.4	0.001	14.9	54.0	28.2	0.008	7.3	49.1	31.2	0.000	14.9	47.5
Jalandhar	44.0	0.002	16.2	71.8	16.6	0.014	3.4	29.8	28.8	0.001	11.7	46.0
Hoshiarpur	52.8	0.000	26.0	79.6	44.4	0.000	21.6	67.2	48.4	0.000	30.2	66.6
Sangrur	21.4	0.032	1.9	41.0	62.9	0.000	29.7	96.1	42.2	0.000	24.1	60.3
Fatehgarh sahib	73.5	0.000	37.0	110.0	23.2	0.020	3.7	42.7	46.8	0.000	25.2	68.4
Ludhiana	32.4	0.004	10.6	54.3	26.6	0.001	11.5	41.8	29.1	0.000	15.7	42.5
Moga	76.3	0.000	47.3	105.3	60.0	0.000	34.1	85.8	67.5	0.000	49.2	85.8
Firozpur	23.8	0.012	5.2	42.4	33.5	0.001	13.9	53.0	28.9	0.000	16.2	41.5
Muktsar	38.9	0.008	10.0	67.7	48.5	0.000	28.1	68.9	44.5	0.000	30.1	58.9
Faridkot	15.4	0.015	3.0	27.8	26.0	0.001	11.0	41.0	21.5	0.000	12.4	30.5
Bathinda	64.7	0.000	29.4	100.0	25.1	0.027	2.8	47.3	43.9	0.000	27.7	60.0
Mansa	25.1	0.002	9.2	40.9	36.3	0.002	13.2	59.4	31.0	0.000	16.2	45.8
Patiala	37.1	0.002	14.1	60.2	27.4	0.001	10.7	44.1	31.7	0.000	17.3	46.1
Amritsar	40.3	0.000	18.2	62.5	25.4	0.001	10.4	40.4	32.0	0.000	20.3	43.7
Tarn taran	29.2	0.001	12.0	46.4	38.9	0.001	16.6	61.1	34.2	0.000	20.3	48.2
Rupnagar	58.9	0.000	30.9	86.9	13.0	0.073	-1.2	27.3	34.6	0.000	15.9	53.2
Sahibzada ajit singh nagar	32.4	0.001	13.4	51.4	30.4	0.003	10.6	50.3	31.3	0.000	17.9	44.7
Shahid bhagat singh nagar	42.6	0.002	15.9	69.2	38.1	0.000	17.9	58.3	40.2	0.000	22.5	57.9
Barnala	58.7	0.005	17.8	99.5	57.6	0.001	23.9	91.2	58.2	0.000	32.5	83.9
Chandigarh	30.6	0.003	10.7	50.5	27.1	0.009	6.7	47.5	28.9	0.000	14.3	43.5
Uttarkashi	62.8	0.000	38.5	87.2	70.8	0.000	43.6	98.1	67.2	0.000	49.3	85.2
Chamoli	41.0	0.000	18.6	63.3	42.2	0.000	20.7	63.7	41.5	0.000	26.5	56.6
Rudraprayag	22.8	0.031	2.1	43.4	35.7	0.001	14.0	57.5	29.5	0.000	15.6	43.4
Tehri garhwal	31.2	0.000	15.6	46.9	37.5	0.000	19.1	55.8	34.5	0.000	21.6	47.4
Dehradun	30.3	0.000	15.2	45.4	31.8	0.000	17.0	46.6	31.1	0.000	23.4	38.9
Garhwal	25.7	0.007	7.1	44.2	27.5	0.020	4.3	50.7	26.8	0.000	14.0	39.6
Pithoragarh	23.1	0.004	7.5	38.8	61.3	0.000	31.3	91.3	43.4	0.000	27.4	59.3
Bageshwar	60.4	0.000	36.3	84.5	53.6	0.000	29.5	77.6	56.8	0.000	40.2	73.5
Almora	20.3	0.003	6.9	33.7	70.6	0.000	33.6	107.6	46.2	0.000	28.5	63.9
Champawat	70.4	0.000	43.6	97.3	45.0	0.000	23.6	66.3	57.3	0.000	41.8	72.8
Nainital	60.6	0.000	43.1	78.1	41.6	0.000	27.0	56.1	50.7	0.000	39.3	62.1
Udham singh nagar	47.1	0.000	33.7	60.5	49.3	0.000	36.3	62.3	48.2	0.000	37.2	59.3
Hardwar	86.4	0.000	68.8	104.0	60.9	0.000	51.2	70.7	73.3	0.000	62.5	84.0
Panchkula	11.3	0.098	-2.1	24.7	22.6	0.001	9.8	35.4	18.0	0.000	8.2	27.9
Ambala	29.8	0.002	10.6	49.0	39.3	0.000	21.1	57.5	35.3	0.000	23.2	47.4
Yamunanagar	35.8	0.009	8.9	62.8	33.0	0.000	18.1	47.9	34.1	0.000	20.6	47.6
Kurukshetra	38.2	0.002	14.5	61.9	27.5	0.001	11.2	43.9	32.1	0.000	15.4	48.7
Kaithal	70.1	0.000	41.8	98.5	47.6	0.000	25.7	69.5	58.1	0.000	44.3	71.8
Karnal	35.9	0.002	13.2	58.6	24.6	0.009	6.2	43.1	30.0	0.000	16.8	43.1
Panipat	14.5	0.019	2.4	26.7	15.5	0.022	2.3	28.8	15.1	0.001	6.0	24.1
Sonipat	9.9	0.092	-1.6	21.4	14.5	0.009	3.6	25.4	12.5	0.001	5.0	19.9
Jind	43.7	0.000	24.7	62.7	20.6	0.015	3.9	37.2	32.1	0.000	20.8	43.4
Fatehabad	32.5	0.001	13.3	51.7	26.4	0.003	9.3	43.5	29.2	0.000	15.6	42.8

Sirsa	57.5	0.000	31.1	83.9	46.5	0.000	28.9	64.1	51.7	0.000	39.6	63.8
Hisar	57.9	0.000	26.5	89.4	33.0	0.000	16.7	49.4	44.2	0.000	28.5	59.9
Bhiwani	48.6	0.000	26.8	70.3	60.9	0.000	35.3	86.5	55.1	0.000	39.4	70.7
Rohtak	49.3	0.000	25.2	73.4	53.3	0.000	30.8	75.9	51.5	0.000	34.8	68.2
Jhajjar	23.9	0.013	5.1	42.6	42.1	0.000	18.6	65.6	34.5	0.000	18.7	50.3
Mahendragarh	44.4	0.000	21.6	67.3	29.4	0.000	13.3	45.5	35.8	0.000	22.9	48.7
Rewari	26.1	0.003	8.8	43.5	35.7	0.000	18.8	52.5	31.7	0.000	18.8	44.6
Gurgaon	26.8	0.013	5.6	48.0	8.6	0.089	-1.3	18.5	16.8	0.010	4.1	29.5
Mewat	102.9	0.000	81.9	123.8	87.1	0.000	64.4	109.8	94.6	0.000	81.6	107.7
Faridabad	44.0	0.006	12.9	75.1	29.5	0.001	11.6	47.5	35.9	0.000	24.1	47.6
Palwal	47.9	0.000	31.0	64.8	26.3	0.000	13.2	39.4	36.1	0.000	22.2	49.9
North west	59.7	0.000	31.9	87.4	25.3	0.015	5.0	45.6	40.5	0.000	23.8	57.1
North	41.2	0.004	13.5	69.0	33.8	0.031	3.0	64.7	37.9	0.000	18.9	56.8
North east	50.8	0.000	24.5	77.2	46.1	0.000	23.7	68.5	48.2	0.000	28.7	67.7
East	48.1	0.003	16.1	80.0	26.9	0.043	0.9	52.8	37.7	0.000	19.0	56.5
New delhi	12.3	0.135	-3.8	28.5	5.0	0.383	-6.2	16.2	8.0	0.093	-1.3	17.2
Central	32.6	0.013	7.0	58.2	16.0	0.079	-1.8	33.7	24.6	0.000	11.7	37.5
West	23.8	0.062	-1.2	48.9	19.2	0.014	3.9	34.4	21.3	0.011	4.9	37.6
South west	9.4	0.097	-1.7	20.4	6.3	0.052	0.0	12.6	7.7	0.001	3.0	12.4
South	31.9	0.021	4.8	58.9	49.7	0.000	25.7	73.7	42.2	0.000	20.1	64.2
Ganganagar	43.7	0.000	22.0	65.3	50.5	0.000	27.3	73.7	47.4	0.000	33.4	61.4
Hanumangarh	43.4	0.000	20.2	66.6	44.2	0.000	23.7	64.7	43.7	0.000	28.5	58.9
Bikaner	41.5	0.000	30.2	52.7	55.7	0.000	39.9	71.5	48.8	0.000	40.8	56.8
Churu	46.2	0.000	28.3	64.0	30.7	0.000	16.8	44.6	38.0	0.000	27.4	48.6
Jhunjhunun	37.9	0.000	20.0	55.8	43.8	0.000	25.3	62.3	41.1	0.000	24.8	57.4
Alwar	43.5	0.000	25.6	61.4	45.4	0.000	27.1	63.7	44.5	0.000	31.1	58.0
Bharatpur	48.4	0.000	28.9	67.9	45.6	0.000	28.4	62.9	47.0	0.000	34.9	59.1
Dhaulpur	72.7	0.000	49.5	96.0	57.3	0.000	39.0	75.6	64.2	0.000	48.1	80.3
Karauli	120.0	0.000	93.4	146.5	68.8	0.000	47.2	90.4	92.9	0.000	70.6	115.3
Sawai madhopur	58.1	0.000	37.1	79.2	58.4	0.000	41.9	74.9	58.2	0.000	45.4	71.0
Dausa	69.5	0.000	41.4	97.6	57.5	0.000	37.0	77.9	63.3	0.000	48.7	77.8
Jaipur	64.0	0.000	49.6	78.5	57.5	0.000	42.2	72.8	60.5	0.000	48.4	72.6
Sikar	38.8	0.000	21.0	56.6	43.4	0.000	27.9	58.9	41.3	0.000	27.1	55.4
Nagaur	26.9	0.004	8.7	45.1	41.5	0.000	26.1	57.0	34.8	0.000	21.4	48.2
Jodhpur	56.5	0.000	37.0	76.0	52.8	0.000	38.0	67.7	54.6	0.000	44.2	64.9
Jaisalmer	61.5	0.000	43.2	79.7	50.4	0.000	33.1	67.6	55.4	0.000	38.6	72.2
Barmer	63.0	0.000	43.3	82.6	66.5	0.000	48.8	84.2	64.9	0.000	49.7	80.1
Jalor	59.5	0.000	36.3	82.7	66.6	0.000	47.2	86.0	63.4	0.000	48.6	78.2
Sirohi	74.6	0.000	45.6	103.5	66.2	0.000	40.7	91.7	70.3	0.000	56.3	84.3
Pali	68.5	0.000	44.0	93.0	59.4	0.000	39.4	79.5	63.8	0.000	44.8	82.8
Ajmer	38.9	0.000	26.9	50.9	37.4	0.000	24.6	50.2	38.2	0.000	27.3	49.1
Tonk	64.4	0.000	36.3	92.5	36.7	0.001	14.2	59.2	50.4	0.000	33.1	67.8
Bundi	65.1	0.000	37.9	92.4	57.3	0.000	34.0	80.7	61.0	0.000	44.0	78.1
Bhilwara	47.4	0.000	27.7	67.1	49.5	0.000	24.7	74.3	48.5	0.000	32.1	64.8
Rajsamand	44.3	0.000	23.5	65.1	79.8	0.000	55.6	104.0	64.1	0.000	48.5	79.7
Dungarpur	46.7	0.000	24.7	68.7	38.8	0.000	22.2	55.4	42.6	0.000	30.1	55.2
Banswara	35.9	0.000	18.9	52.8	47.4	0.000	32.0	62.8	41.8	0.000	30.2	53.4
Chittaurgarh	37.3	0.000	17.7	57.0	69.4	0.000	43.6	95.2	54.3	0.000	38.5	70.2
Kota	44.7	0.000	30.2	59.1	39.6	0.000	26.7	52.4	42.0	0.000	32.5	51.5
Baran	52.2	0.000	27.3	77.1	47.6	0.000	26.9	68.3	49.8	0.000	33.2	66.5
Jhalawar	45.4	0.000	27.2	63.7	80.3	0.000	59.3	101.3	64.8	0.000	47.5	82.1
Udaipur	57.1	0.000	35.4	78.8	65.7	0.000	41.2	90.2	61.3	0.000	46.1	76.5
Pratapgarh	72.4	0.000	47.8	97.0	57.8	0.000	37.8	77.9	64.7	0.000	51.4	78.0
Saharanpur	93.5	0.000	73.7	113.2	60.3	0.000	47.1	73.5	76.0	0.000	62.7	89.3
Muzaffarnagar	70.2	0.000	46.4	94.0	59.2	0.000	42.0	76.5	64.4	0.000	50.2	78.5
Bijnor	65.3	0.000	45.3	85.3	56.2	0.000	37.6	74.8	60.5	0.000	44.3	76.7
Moradabad	102.0	0.000	85.4	118.7	91.0	0.000	72.5	109.5	96.4	0.000	83.1	109.7
Rampur	80.2	0.000	57.9	102.5	60.3	0.000	40.0	80.5	70.0	0.000	57.8	82.1
Jyotiba phule nagar	90.6	0.000	65.3	115.8	75.6	0.000	50.3	100.8	82.9	0.000	67.7	98.0
Meerut	86.0	0.000	66.1	106.0	71.5	0.000	57.6	85.4	78.1	0.000	66.8	89.4
Baghpat	64.6	0.000	44.2	85.0	42.4	0.000	24.5	60.3	52.4	0.000	40.0	64.8
Ghaziabad	64.0	0.000	48.6	79.3	79.2	0.000	52.5	106.0	72.1	0.000	58.5	85.7
Gautam buddha nagar	75.3	0.000	57.8	92.8	68.0	0.000	46.6	89.5	71.3	0.000	58.2	84.5
Bulandshahr	63.7	0.000	48.0	79.5	81.3	0.000	60.3	102.3	73.0	0.000	53.8	92.2
Aligarh	100.8	0.000	81.6	120.1	91.4	0.000	73.1	109.7	95.8	0.000	83.4	108.3
Mahamaya nagar	85.9	0.000	61.2	110.7	86.8	0.000	60.9	112.6	86.4	0.000	67.4	105.3
Mathura	97.8	0.000	71.5	124.1	64.5	0.000	47.6	81.4	79.7	0.000	62.8	96.6
Agra	80.4	0.000	65.0	95.8	71.0	0.000	55.4	86.6	75.5	0.000	65.4	85.5
Firozabad	109.2	0.000	88.6	129.7	66.6	0.000	50.5	82.7	87.1	0.000	74.9	99.2
Mainpuri	104.0	0.000	75.8	132.2	87.7	0.000	66.8	108.7	95.6	0.000	78.8	112.3
Budaun	119.6	0.000	95.2	144.0	115.4	0.000	92.5	138.3	117.3	0.000	101.6	133.0
Bareilly	91.2	0.000	74.2	108.2	80.7	0.000	59.2	102.2	85.8	0.000	76.0	95.7
Pilibhit	97.2	0.000	72.9	121.4	98.7	0.000	71.0	126.4	97.8	0.000	80.1	115.6
Shahjahanpur	94.7	0.000	73.3	116.1	112.0	0.000	85.5	138.6	103.4	0.000	84.8	122.0
Kheri	128.4	0.000	100.0	156.8	87.1	0.000	61.4	112.8	107.5	0.000	89.9	125.2
Sitapur	131.9	0.000	103.6	160.2	94.8	0.000	70.5	119.1	112.2	0.000	92.5	132.0
Hardoi	83.5	0.000	48.8	118.2	93.7	0.000	69.3	118.2	89.1	0.000	72.1	106.0
Unnao	87.0	0.000	61.2	112.9	70.5	0.000	50.9	90.1	78.5	0.000	64.2	92.9
Lucknow	58.8	0.000	38.8	78.9	49.4	0.000	31.4	67.5	53.8	0.000	40.2	67.5
Rae bareli	95.1	0.000	68.8	121.4	75.0	0.000	54.6	95.3	84.3	0.000	63.9	104.7
Farrukhabad	105.9	0.000	80.9	130.9	93.7	0.000	68.8	118.7	99.8	0.000	83.2	116.3
Kannauj	103.0	0.000	76.9	129.0	91.0	0.000	67.3	114.7	96.8	0.000	79.2	114.5
Etawah	69.6	0.000	47.1	92.2	76.8	0.000	54.7	98.9	73.8	0.000	54.3	93.3

Auraiya	70.8	0.000	43.7	97.9	48.6	0.000	29.2	68.1	59.0	0.000	43.3	74.6
Kanpur dehat	67.4	0.000	39.6	95.2	70.8	0.000	47.6	94.0	69.2	0.000	55.0	83.3
Kanpur nagar	41.8	0.000	19.0	64.7	70.2	0.000	43.6	96.7	57.3	0.000	43.0	71.6
Jalaun	65.3	0.000	30.1	100.5	77.7	0.000	48.9	106.5	72.7	0.000	49.8	95.6
Jhansi	90.2	0.000	66.7	113.6	87.6	0.000	65.2	110.1	88.7	0.000	75.5	102.0
Lalitpur	84.7	0.000	59.7	109.7	70.9	0.000	42.2	99.5	77.7	0.000	58.1	97.3
Hamirpur	51.9	0.000	28.2	75.7	50.3	0.000	25.8	74.7	51.0	0.000	33.2	68.9
Mahoba	55.0	0.000	31.2	78.8	74.5	0.000	45.9	103.0	65.1	0.000	46.6	83.6
Banda	67.4	0.000	41.1	93.7	71.4	0.000	44.0	98.7	69.4	0.000	51.1	87.8
Chitrakoot	80.7	0.000	58.6	102.8	94.0	0.000	67.6	120.5	87.6	0.000	70.5	104.7
Fatehpur	81.7	0.000	59.3	104.2	64.6	0.000	36.5	92.8	73.4	0.000	55.1	91.7
Pratapgarh	77.1	0.000	47.6	106.5	71.8	0.000	45.8	97.8	74.3	0.000	57.3	91.3
Kaushambi	112.1	0.000	85.2	139.0	129.8	0.000	102.1	157.6	121.2	0.000	100.6	141.8
Allahabad	73.3	0.000	51.8	94.9	70.9	0.000	45.7	96.2	72.1	0.000	57.1	87.2
Bara banki	58.3	0.000	38.8	77.8	63.6	0.000	39.8	87.3	61.0	0.000	44.2	77.7
Faizabad	79.3	0.000	54.4	104.3	68.2	0.000	49.0	87.5	73.6	0.000	54.9	92.2
Ambedkar nagar	89.2	0.000	63.4	114.9	69.6	0.000	49.9	89.4	79.2	0.000	62.3	96.0
Sultanpur	87.2	0.000	59.7	114.7	81.7	0.000	61.1	102.2	84.2	0.000	66.3	102.2
Bahraich	94.2	0.000	76.0	112.3	70.9	0.000	56.3	85.5	82.3	0.000	67.8	96.8
Shrawasti	120.2	0.000	93.9	146.4	118.1	0.000	91.7	144.5	119.1	0.000	102.7	135.4
Balrampur	75.6	0.000	57.4	93.8	62.6	0.000	45.9	79.3	68.7	0.000	53.5	83.9
Gonda	110.7	0.000	88.1	133.4	122.7	0.000	95.6	149.9	116.7	0.000	95.2	138.2
Siddharth nagar	88.5	0.000	69.4	107.5	97.9	0.000	79.3	116.5	93.4	0.000	78.0	108.8
Basti	75.1	0.000	47.8	102.5	85.4	0.000	61.7	109.1	80.6	0.000	65.8	95.3
Sant kabir nagar	69.1	0.000	48.2	90.1	72.5	0.000	51.4	93.6	71.0	0.000	55.7	86.3
Mahrajganj	54.7	0.000	35.4	73.9	80.1	0.000	64.2	96.0	68.6	0.000	55.2	81.9
Gorakhpur	60.1	0.000	33.3	86.8	71.8	0.000	49.9	93.6	66.2	0.000	52.1	80.2
Kushinagar	56.4	0.000	35.5	77.3	72.7	0.000	56.5	89.0	65.3	0.000	53.4	77.2
Deoria	69.6	0.000	49.2	90.0	68.6	0.000	49.2	87.9	69.0	0.000	53.5	84.5
Azamgarh	74.1	0.000	52.5	95.6	57.6	0.000	36.6	78.6	65.8	0.000	50.8	80.7
Mau	62.1	0.000	45.7	78.5	79.0	0.000	57.7	100.2	70.9	0.000	54.9	86.9
Ballia	70.9	0.000	47.3	94.6	76.7	0.000	55.1	98.2	73.9	0.000	57.9	90.0
Jaunpur	75.6	0.000	49.4	101.9	82.8	0.000	60.3	105.3	79.3	0.000	63.8	94.7
Ghazipur	72.2	0.000	48.0	96.4	74.7	0.000	53.3	96.1	73.3	0.000	58.6	88.1
Chandauli	53.2	0.000	35.2	71.2	63.7	0.000	41.7	85.6	58.6	0.000	44.1	73.1
Varanasi	76.5	0.000	58.4	94.6	53.0	0.000	37.1	68.9	64.3	0.000	50.0	78.6
Sant ravidas nagar (bhadohi)	91.6	0.000	69.1	114.1	112.3	0.000	87.1	137.4	102.2	0.000	83.6	120.9
Mirzapur	85.2	0.000	60.4	110.1	81.3	0.000	57.1	105.4	83.3	0.000	70.4	96.1
Sonbhadra	66.1	0.000	44.9	87.4	63.6	0.000	45.0	82.3	64.8	0.000	48.7	80.8
Etah	92.9	0.000	66.8	119.0	94.3	0.000	69.3	119.3	93.7	0.000	79.3	108.1
Kanshiram nagar	109.3	0.000	82.4	136.2	116.6	0.000	89.2	144.0	113.3	0.000	95.6	130.9
Pashchim champaran	52.6	0.000	35.9	69.3	56.4	0.000	40.3	72.5	54.5	0.000	42.3	66.7
Purba champaran	74.5	0.000	56.1	93.0	72.6	0.000	52.9	92.3	73.5	0.000	56.9	90.1
Sheohar	80.2	0.000	62.3	98.1	87.3	0.000	67.2	107.4	84.0	0.000	69.8	98.3
Sitamarhi	73.9	0.000	51.8	96.1	74.5	0.000	53.8	95.3	74.3	0.000	57.9	90.7
Madhubani	70.9	0.000	50.2	91.5	47.2	0.000	30.4	64.0	58.8	0.000	45.1	72.5
Supaul	49.3	0.000	36.2	62.4	57.9	0.000	41.9	73.8	53.6	0.000	42.8	64.5
Araria	78.7	0.000	57.3	100.0	82.8	0.000	64.2	101.3	80.7	0.000	64.0	97.4
Kishanganj	44.0	0.000	24.7	63.3	56.6	0.000	36.6	76.6	50.8	0.000	37.6	64.0
Purnia	76.6	0.000	58.2	94.9	86.7	0.000	70.0	103.3	81.8	0.000	68.4	95.3
Katihar	54.4	0.000	35.5	73.4	82.3	0.000	60.1	104.4	68.9	0.000	54.0	83.7
Madhepura	67.1	0.000	49.7	84.6	63.6	0.000	46.4	80.7	65.4	0.000	54.0	76.9
Saharsa	59.2	0.000	38.5	79.9	75.0	0.000	58.7	91.2	67.0	0.000	54.2	79.9
Darbhanga	38.6	0.000	23.4	53.8	39.6	0.000	25.4	53.7	39.1	0.000	29.2	49.0
Muzaffarpur	47.4	0.000	30.9	63.9	36.4	0.000	21.3	51.5	41.6	0.000	31.3	52.0
Gopalganj	37.1	0.000	24.6	49.6	62.7	0.000	44.1	81.2	49.8	0.000	37.2	62.4
Siwan	47.4	0.000	27.9	66.8	49.4	0.000	34.6	64.2	48.5	0.000	36.5	60.6
Saran	41.9	0.000	24.5	59.3	55.3	0.000	37.8	72.8	48.8	0.000	37.6	60.0
Vaishali	52.3	0.000	36.4	68.2	63.6	0.000	43.6	83.5	58.5	0.000	44.1	72.9
Samastipur	51.1	0.000	31.8	70.5	41.0	0.000	27.7	54.3	45.9	0.000	35.9	55.9
Begusarai	49.3	0.000	29.7	69.0	54.5	0.000	35.1	73.8	51.9	0.000	39.4	64.5
Khagaria	52.6	0.000	35.6	69.6	68.2	0.000	51.7	84.6	60.5	0.000	48.7	72.4
Bhagalpur	27.5	0.000	17.2	37.8	48.5	0.000	32.4	64.5	38.6	0.000	27.2	50.0
Banka	63.2	0.000	42.6	83.8	47.4	0.000	30.9	64.0	54.8	0.000	44.0	65.6
Munger	74.7	0.000	54.8	94.7	64.9	0.000	46.2	83.6	69.6	0.000	56.4	82.9
Lakhisarai	84.1	0.000	63.6	104.7	63.1	0.000	48.0	78.2	73.0	0.000	60.4	85.7
Sheikhpura	59.9	0.000	44.0	75.7	61.2	0.000	41.8	80.7	60.6	0.000	48.0	73.2
Nalanda	47.1	0.000	31.1	63.1	48.4	0.000	31.5	65.3	47.8	0.000	35.4	60.2
Patna	50.9	0.000	39.1	62.8	54.7	0.000	40.5	69.0	52.9	0.000	44.1	61.6
Bhojpur	56.4	0.000	39.4	73.3	49.5	0.000	29.1	69.9	52.8	0.000	38.5	67.1
Buxar	62.7	0.000	40.1	85.2	62.8	0.000	48.0	77.6	62.7	0.000	47.0	78.4
Kaimur (bhabua)	63.9	0.000	46.0	81.7	86.1	0.000	65.7	106.4	75.1	0.000	62.8	87.4
Rohtas	49.1	0.000	33.5	64.7	79.6	0.000	58.7	100.4	64.6	0.000	50.7	78.5
Aurangabad	66.6	0.000	43.5	89.7	55.7	0.000	38.8	72.6	60.9	0.000	46.0	75.8
Gaya	73.7	0.000	52.7	94.7	75.3	0.000	57.3	93.3	74.5	0.000	59.9	89.2
Nawada	52.7	0.000	36.8	68.6	55.1	0.000	40.8	69.4	53.9	0.000	41.6	66.3
Jamui	49.4	0.000	33.9	65.0	69.9	0.000	51.9	87.8	60.0	0.000	50.1	69.9
Jehanabad	65.3	0.000	41.3	89.3	62.0	0.000	44.2	79.7	63.6	0.000	44.7	82.5
Arwal	49.3	0.000	31.9	66.6	55.8	0.000	38.0	73.6	52.5	0.000	39.9	65.2
North district	14.7	0.144	-5.0	34.3	17.0	0.008	4.4	29.6	16.0	0.001	6.2	25.8
West district	65.0	0.000	29.1	100.9	75.7	0.000	46.2	105.2	70.8	0.000	47.0	94.7
South district	2.2	0.304	-2.0	6.5	7.8	0.166	-3.2	18.7	5.0	0.087	-0.7	10.7
East district	22.3	0.002	8.2	36.3	16.4	0.005	5.0	27.8	19.2	0.000	10.5	27.9

Tawang	30.4	0.020	4.8	55.9	23.6	0.006	6.6	40.6	26.8	0.000	14.1	39.6
West kameng	27.3	0.007	7.3	47.2	29.6	0.013	6.3	52.9	28.4	0.000	14.5	42.4
East kameng	29.4	0.000	13.4	45.5	30.8	0.000	15.4	46.1	30.1	0.000	18.8	41.4
Papumpare	18.8	0.014	3.8	33.7	18.4	0.005	5.6	31.3	18.6	0.000	9.2	28.0
Upper subansiri	21.9	0.011	5.1	38.8	35.0	0.000	17.0	53.0	28.6	0.000	17.0	40.3
West siang	5.0	0.328	-5.0	14.9	18.5	0.038	1.0	35.9	12.3	0.027	1.4	23.2
East siang	0.0	0.000	0.0	0.0	5.8	0.126	-1.6	13.3	3.1	0.124	-0.8	7.0
Upper siang	7.5	0.190	-3.7	18.7	12.0	0.106	-2.6	26.5	9.7	0.040	0.5	19.0
Changlang	63.2	0.000	39.5	86.9	23.4	0.000	11.6	35.2	43.6	0.000	32.5	54.7
Tirap	47.1	0.000	28.0	66.3	60.0	0.000	41.3	78.7	53.9	0.000	39.7	68.2
Lower subansiri	28.1	0.007	7.5	48.6	18.6	0.066	-1.2	38.5	23.2	0.003	8.1	38.4
Kurung kumey	26.4	0.001	10.5	42.3	34.0	0.000	18.3	49.6	30.3	0.000	18.8	41.8
Dibang valley	6.3	0.228	-4.0	16.6	13.2	0.071	-1.1	27.6	9.8	0.036	0.7	18.9
Lower dibang valley	20.8	0.025	2.6	39.0	33.7	0.000	17.3	50.0	27.6	0.000	16.6	38.6
Lohit	48.4	0.000	26.1	70.8	92.0	0.000	64.1	119.9	71.6	0.000	52.8	90.4
Anjaw	19.4	0.003	6.6	32.2	24.4	0.001	10.3	38.5	22.1	0.002	8.4	35.7
Mon	12.8	0.003	4.4	21.3	28.1	0.000	15.3	40.9	20.8	0.000	12.5	29.2
Mokokchung	29.4	0.024	3.9	54.8	31.9	0.015	6.1	57.6	30.7	0.007	8.3	53.1
Zunheboto	15.5	0.026	1.9	29.0	15.1	0.053	-0.2	30.4	15.3	0.000	7.9	22.6
Wokha	33.0	0.001	14.1	51.9	37.9	0.003	13.3	62.5	35.5	0.000	16.6	54.5
Dimapur	38.1	0.000	24.5	51.6	38.3	0.000	21.0	55.6	38.2	0.000	26.3	50.0
Phek	41.2	0.000	25.9	56.6	27.2	0.000	12.4	42.0	34.1	0.000	22.8	45.3
Tuensang	53.5	0.000	35.2	71.7	43.2	0.000	19.8	66.6	48.4	0.000	36.9	59.8
Longleng	29.0	0.001	11.3	46.6	16.4	0.017	2.9	29.8	22.4	0.000	11.6	33.3
Kiphire	59.1	0.000	37.1	81.1	80.8	0.000	54.2	107.5	70.4	0.000	51.7	89.2
Kohima	30.0	0.001	11.8	48.1	30.0	0.000	13.5	46.5	30.0	0.000	18.6	41.4
Peren	36.8	0.000	21.1	52.6	51.1	0.000	29.2	73.0	44.2	0.000	30.1	58.4
Senapati (excluding 3 sub-divisions)	49.0	0.000	31.0	66.9	39.2	0.000	23.8	54.6	43.8	0.000	29.9	57.7
Tamenglong	28.0	0.000	13.4	42.5	35.2	0.000	19.3	51.2	31.6	0.000	20.1	43.2
Churachandpur	15.9	0.012	3.5	28.4	43.7	0.000	21.4	65.9	30.2	0.000	16.9	43.6
Bishnupur	16.9	0.001	6.6	27.2	21.3	0.000	10.4	32.2	19.2	0.000	11.8	26.5
Thoubal	28.2	0.000	15.3	41.0	31.5	0.000	20.1	42.8	29.9	0.000	21.5	38.3
Imphal west	13.8	0.004	4.4	23.3	24.6	0.000	12.1	37.2	19.1	0.000	10.6	27.6
Imphal east	20.8	0.000	11.6	30.0	31.0	0.000	18.6	43.5	26.2	0.000	19.5	32.9
Ukhrul	32.4	0.001	12.9	51.9	39.2	0.000	23.1	55.3	36.0	0.000	23.5	48.6
Chandel	33.9	0.000	16.3	51.6	34.5	0.000	18.9	50.1	34.2	0.000	22.6	45.9
Mamit	42.8	0.000	21.9	63.7	66.9	0.000	43.1	90.8	55.2	0.000	39.0	71.3
Kolasib	43.3	0.000	28.0	58.7	61.3	0.000	41.4	81.3	52.5	0.000	38.9	66.2
Aizawl	70.0	0.000	35.9	104.0	45.6	0.000	25.1	66.2	57.4	0.000	40.1	74.7
Champhai	29.9	0.000	16.1	43.8	37.4	0.000	24.0	50.8	33.7	0.000	25.5	41.8
Serchhip	42.7	0.000	25.5	59.9	45.4	0.000	28.8	62.0	44.1	0.000	32.3	56.0
Lunglei	58.9	0.000	40.1	77.6	47.2	0.000	26.7	67.6	53.0	0.000	39.4	66.5
Lawngtlai	36.2	0.000	19.0	53.3	57.0	0.000	38.0	76.0	46.7	0.000	31.4	62.0
Saiha	68.8	0.000	49.3	88.3	90.5	0.000	71.2	109.8	80.0	0.000	66.0	94.1
West tripura	24.2	0.000	11.6	36.7	24.5	0.000	11.5	37.5	24.2	0.000	16.2	32.3
South tripura	30.9	0.003	10.1	51.6	3.6	0.214	-2.1	9.2	16.5	0.004	5.4	27.7
Dhalai	21.0	0.014	4.2	37.7	46.5	0.000	25.6	67.3	33.9	0.000	21.0	46.7
North tripura	49.9	0.000	26.7	73.1	69.5	0.000	39.5	99.4	60.1	0.000	43.9	76.2
West garo hills	28.0	0.000	14.3	41.8	41.9	0.000	27.8	55.9	35.1	0.000	23.6	46.7
East garo hills	14.2	0.006	4.1	24.4	20.3	0.000	10.7	29.9	17.2	0.000	7.9	26.4
South garo hills	38.0	0.000	19.6	56.3	43.9	0.000	24.2	63.6	41.0	0.000	24.9	57.1
West khasi hills	53.7	0.000	35.0	72.4	54.8	0.000	39.0	70.7	54.3	0.000	42.8	65.7
Ribhoi	59.1	0.000	42.1	76.1	74.0	0.000	53.7	94.3	66.4	0.000	52.4	80.3
East khasi hills	38.9	0.000	26.2	51.7	42.7	0.000	30.5	55.0	40.9	0.000	31.5	50.3
Jaintia hills	41.0	0.000	25.1	56.9	35.5	0.000	20.1	51.0	38.2	0.000	28.8	47.6
Kokrajhar	59.4	0.000	34.9	84.0	49.3	0.000	26.4	72.1	54.8	0.000	39.0	70.6
Dhubri	32.8	0.000	18.1	47.6	56.7	0.000	35.3	78.0	45.3	0.000	31.8	58.8
Goalpara	50.5	0.000	28.1	72.9	46.7	0.000	29.7	63.7	48.5	0.000	34.4	62.6
Barpeta	40.6	0.000	22.7	58.4	46.5	0.000	27.5	65.6	43.8	0.000	31.5	56.0
Morigaon	67.4	0.000	42.5	92.3	82.6	0.000	55.1	110.0	75.2	0.000	58.6	91.9
Nagaon	64.3	0.000	42.2	86.3	75.3	0.000	51.4	99.3	70.0	0.000	52.9	87.0
Sonitpur	32.9	0.001	13.5	52.3	65.1	0.000	37.2	93.0	49.3	0.000	34.7	64.0
Lakhimpur	43.8	0.000	19.3	68.3	56.4	0.000	34.5	78.4	50.1	0.000	33.1	67.1
Dhemaji	59.7	0.000	36.8	82.5	76.1	0.000	52.1	100.0	68.2	0.000	51.7	84.6
Tinsukia	78.6	0.000	52.1	105.1	86.1	0.000	59.1	113.1	82.7	0.000	66.1	99.3
Dibrugarh	26.7	0.009	6.7	46.8	31.0	0.001	13.2	48.7	28.9	0.000	16.0	41.9
Sivasagar	66.7	0.000	40.4	93.0	89.1	0.000	56.2	122.0	77.3	0.000	63.6	91.1
Jorhat	47.8	0.000	29.2	66.4	56.2	0.000	27.9	84.6	52.2	0.000	33.5	70.8
Golaghat	45.4	0.000	24.6	66.2	58.7	0.000	37.9	79.4	52.1	0.000	35.1	69.2
Karbi anglong	57.2	0.000	37.4	77.1	83.3	0.000	52.9	113.7	70.7	0.000	55.0	86.3
Dima hasao	67.2	0.000	44.3	90.1	85.8	0.000	58.4	113.2	76.7	0.000	56.8	96.6
Cachar	82.5	0.000	51.6	113.3	72.3	0.000	45.5	99.1	77.3	0.000	59.0	95.7
Karimganj	76.9	0.000	52.9	100.9	95.6	0.000	72.6	118.6	86.7	0.000	71.5	101.9
Hailakandi	66.2	0.000	41.9	90.6	76.0	0.000	51.0	101.0	71.2	0.000	53.8	88.6
Bongaigaon	28.1	0.000	12.5	43.6	41.8	0.000	20.0	63.6	35.1	0.000	20.4	49.8
Chirang	55.8	0.000	38.9	72.6	45.7	0.000	22.8	68.7	50.6	0.000	34.4	66.8
Kamrup	23.4	0.003	8.1	38.6	59.4	0.000	37.8	80.9	42.3	0.000	28.4	56.1
Kamrup metropolitan	43.9	0.002	16.3	71.5	44.5	0.000	20.2	68.8	44.1	0.000	26.9	61.4
Nalbari	35.0	0.001	13.8	56.1	40.9	0.001	17.2	64.7	38.1	0.000	23.1	53.1
Baksa	40.7	0.000	23.1	58.3	51.6	0.000	33.0	70.3	46.2	0.000	29.3	63.2
Darrang	42.3	0.000	23.7	61.0	83.7	0.000	56.6	110.8	63.7	0.000	47.2	80.2
Udalguri	37.9	0.000	22.2	53.5	56.0	0.000	34.7	77.2	47.1	0.000	33.9	60.3
Darjiling	21.9	0.030	2.1	41.7	21.4	0.047	0.3	42.5	21.8	0.003	7.4	36.2

Jalpaiguri	25.8	0.012	5.6	45.9	41.7	0.000	23.4	60.0	34.1	0.000	19.1	49.1
Koch bihar	38.7	0.001	16.0	61.5	62.7	0.000	33.7	91.7	51.0	0.000	29.8	72.2
Uttar dinajpur	45.3	0.000	25.5	65.1	62.0	0.000	42.2	81.8	54.0	0.000	40.4	67.6
Dakshin dinajpur	49.0	0.004	16.0	82.1	31.6	0.000	14.3	48.8	39.4	0.000	23.7	55.0
Maldah	46.8	0.000	26.7	66.9	60.1	0.000	36.1	84.2	53.8	0.000	34.6	73.0
Murshidabad	22.5	0.003	7.6	37.5	60.0	0.000	35.5	84.5	41.8	0.000	28.4	55.1
Birbhum	36.1	0.006	10.6	61.6	31.0	0.000	15.4	46.7	33.4	0.000	17.4	49.4
Barddhaman	35.5	0.003	12.2	58.7	33.2	0.029	3.4	63.1	34.2	0.000	20.3	48.2
Nadia	30.2	0.010	7.1	53.3	25.4	0.002	9.3	41.5	27.7	0.000	14.3	41.1
North twenty four parganas	23.0	0.024	3.0	42.9	35.0	0.002	13.2	56.9	29.5	0.001	12.5	46.6
Hugli	58.1	0.000	26.6	89.6	16.3	0.089	-2.5	35.1	36.4	0.000	17.9	54.8
Bankura	40.1	0.003	14.1	66.2	56.8	0.000	29.4	84.2	47.9	0.000	30.7	65.2
Puruliya	30.0	0.000	14.8	45.1	31.8	0.001	13.4	50.1	30.9	0.000	18.0	43.8
Haora	21.6	0.017	3.9	39.3	42.7	0.001	16.4	69.1	32.4	0.000	18.9	45.9
Kolkata	19.5	0.133	-5.9	44.9	20.1	0.154	-7.6	47.8	19.8	0.039	1.0	38.7
South twenty four parganas	27.0	0.001	10.8	43.3	77.5	0.000	43.6	111.3	51.8	0.000	33.3	70.4
Paschim medinipur	15.9	0.057	-0.5	32.2	29.0	0.007	7.9	50.1	22.8	0.001	9.3	36.4
Purba medinipur	33.3	0.004	10.5	56.1	11.3	0.094	-1.9	24.5	22.0	0.000	11.1	33.0
Garhwa	67.8	0.000	43.1	92.4	67.3	0.000	44.5	90.1	67.5	0.000	50.5	84.6
Chatra	74.4	0.000	49.3	99.5	77.4	0.000	54.6	100.2	75.9	0.000	61.8	90.0
Kodarma	31.6	0.000	13.9	49.4	48.7	0.000	27.7	69.7	40.4	0.000	26.9	53.8
Giridih	60.1	0.000	39.0	81.2	57.9	0.000	38.3	77.6	59.0	0.000	46.8	71.2
Deoghar	72.4	0.000	49.0	95.8	45.8	0.000	30.0	61.6	58.1	0.000	42.6	73.6
Godda	72.2	0.000	46.9	97.5	70.4	0.000	45.9	94.9	71.2	0.000	52.0	90.5
Sahibganj	61.0	0.000	41.8	80.2	62.2	0.000	43.0	81.4	61.7	0.000	48.2	75.1
Pakur	41.7	0.000	26.7	56.7	61.5	0.000	40.8	82.2	51.6	0.000	38.9	64.3
Dhanbad	50.3	0.000	35.8	64.8	60.9	0.000	44.1	77.7	55.9	0.000	43.1	68.8
Bokaro	48.9	0.000	33.8	64.1	43.8	0.000	29.4	58.1	46.4	0.000	35.2	57.5
Lohardaga	32.8	0.000	16.0	49.6	52.4	0.000	30.9	73.9	42.5	0.000	30.3	54.8
Purbi singhbhum	14.2	0.007	3.9	24.6	12.3	0.008	3.2	21.3	13.2	0.000	7.6	18.9
Palamu	59.8	0.000	38.9	80.8	63.4	0.000	41.0	85.8	61.6	0.000	44.1	79.1
Latehar	35.9	0.000	16.7	55.0	41.5	0.000	21.9	61.2	38.8	0.000	27.3	50.2
Hazaribagh	54.2	0.000	33.8	74.7	51.8	0.000	34.2	69.4	52.9	0.000	37.9	67.9
Ramgarh	51.3	0.000	37.4	65.3	49.3	0.000	34.5	64.1	50.3	0.000	38.7	61.9
Dumka	66.6	0.000	38.5	94.7	68.3	0.000	46.2	90.4	67.6	0.000	50.0	85.2
Jamtara	60.3	0.000	40.9	79.7	52.2	0.000	35.6	68.8	56.1	0.000	39.5	72.7
Ranchi	57.2	0.000	36.2	78.2	49.4	0.000	31.6	67.2	53.2	0.000	42.3	64.1
Khunti	44.5	0.000	23.8	65.2	40.3	0.000	21.3	59.3	42.4	0.000	29.1	55.7
Gumla	41.4	0.000	22.4	60.4	48.7	0.000	27.7	69.7	45.1	0.000	30.4	59.9
Simdega	103.7	0.000	78.8	128.6	93.6	0.000	67.8	119.5	98.9	0.000	82.4	115.3
Pashchimi singhbhum	94.7	0.000	62.4	126.9	115.0	0.000	83.9	146.1	105.3	0.000	87.6	123.0
Saraikela kharsawan	52.6	0.000	24.2	80.9	56.3	0.000	31.2	81.4	54.6	0.000	36.6	72.6
Bargarh	32.0	0.001	13.8	50.2	35.2	0.001	15.1	55.3	33.6	0.000	18.1	49.2
Jharsuguda	44.4	0.000	29.6	59.1	62.0	0.000	41.3	82.7	53.5	0.000	41.9	65.2
Sambalpur	54.6	0.000	25.4	83.8	57.6	0.000	32.3	82.9	56.2	0.000	35.4	77.0
Debagarh	38.6	0.000	19.0	58.2	86.6	0.000	58.7	114.5	61.7	0.000	45.3	78.2
Sundargarh	36.7	0.000	20.7	52.7	55.1	0.000	38.2	72.0	45.8	0.000	33.6	57.9
Kendujhar	66.1	0.000	40.8	91.4	64.2	0.000	39.4	88.9	64.9	0.000	47.1	82.7
Mayurbhanj	48.2	0.000	27.1	69.3	73.2	0.000	48.8	97.6	60.8	0.000	44.7	77.0
Baleshwar	41.3	0.000	21.3	61.3	27.7	0.005	8.6	46.9	34.1	0.000	19.5	48.7
Bhadrak	39.3	0.000	20.2	58.5	47.8	0.000	25.5	70.0	43.7	0.000	27.0	60.4
Kendrapara	41.8	0.000	21.4	62.2	50.3	0.000	22.7	77.9	46.0	0.000	32.9	59.1
Jagatsinghapur	25.5	0.007	6.8	44.2	58.7	0.001	23.8	93.6	43.3	0.000	24.8	61.9
Cuttack	18.9	0.031	1.7	36.0	39.4	0.004	12.5	66.3	29.2	0.000	13.2	45.2
Jajapur	69.4	0.000	42.6	96.2	36.6	0.000	18.1	55.2	51.9	0.000	35.9	67.8
Dhenkanal	45.3	0.000	20.2	70.4	56.5	0.000	33.4	79.7	51.1	0.000	36.3	66.0
Anugul	63.1	0.000	31.2	95.1	59.0	0.000	34.4	83.6	61.1	0.000	41.0	81.1
Nayagarh	83.2	0.000	52.4	114.1	63.1	0.000	39.6	86.6	72.2	0.000	53.6	90.9
Khordha	17.9	0.001	7.1	28.7	25.9	0.000	14.8	37.0	21.9	0.000	13.5	30.4
Puri	44.3	0.001	19.1	69.6	60.3	0.000	35.1	85.6	52.8	0.000	35.9	69.7
Ganjam	26.2	0.000	11.7	40.8	55.1	0.000	33.1	77.0	41.4	0.000	26.7	56.0
Gajapati	83.1	0.000	57.1	109.1	70.5	0.000	47.1	94.0	76.5	0.000	59.6	93.4
Kandhamal	85.7	0.000	57.9	113.5	101.8	0.000	76.5	127.0	93.8	0.000	76.3	111.4
Baudh	98.8	0.000	68.4	129.1	84.2	0.000	61.0	107.5	91.5	0.000	72.1	110.9
Subarnapur	40.1	0.001	16.8	63.4	64.5	0.000	39.0	90.1	52.8	0.000	38.6	67.0
Balangir	45.4	0.000	24.2	66.7	72.9	0.000	43.9	101.9	59.4	0.000	41.0	77.8
Nuapada	51.0	0.000	31.9	70.2	69.9	0.000	46.8	93.0	60.2	0.000	44.2	76.1
Kalahandi	78.8	0.000	50.8	106.7	75.2	0.000	50.2	100.3	76.8	0.000	54.2	99.4
Rayagada	120.2	0.000	83.3	157.1	141.7	0.000	105.0	178.5	131.5	0.000	108.3	154.6
Nabarangapur	72.7	0.000	54.5	90.8	100.7	0.000	77.5	123.8	86.7	0.000	68.5	104.8
Koraput	73.0	0.000	49.0	97.0	58.0	0.000	35.5	80.6	65.5	0.000	47.3	83.6
Malkangiri	80.8	0.000	58.5	103.0	107.4	0.000	76.1	138.7	94.9	0.000	77.3	112.4
Korea (koriya)	73.8	0.000	51.3	96.3	86.8	0.000	65.9	107.8	80.5	0.000	64.2	96.9
Surguja	76.5	0.000	51.1	101.9	96.3	0.000	64.8	127.8	86.1	0.000	67.2	105.0
Jashpur	68.3	0.000	45.7	90.8	96.8	0.000	69.4	124.2	83.1	0.000	58.2	108.0
Raigarh	73.0	0.000	42.4	103.6	68.4	0.000	39.1	97.7	70.5	0.000	50.2	90.8
Korba	85.0	0.000	67.3	102.7	86.5	0.000	67.0	105.9	85.7	0.000	70.2	101.3
Janjgir - champa	70.8	0.000	40.1	101.4	46.8	0.000	24.9	68.7	58.0	0.000	41.4	74.5
Bilaspur	61.0	0.000	41.3	80.6	83.3	0.000	62.0	104.7	72.0	0.000	58.1	86.0
Kabirdham	65.3	0.000	48.3	82.2	66.1	0.000	43.0	89.2	65.7	0.000	47.3	84.1
Rajnandgaon	46.7	0.000	27.4	66.1	85.4	0.000	58.8	111.9	66.7	0.000	47.8	85.6
Durg	39.8	0.000	26.1	53.5	52.7	0.000	34.4	71.0	46.5	0.000	33.3	59.7
Raipur	59.1	0.000	41.1	77.0	67.8	0.000	48.1	87.4	63.4	0.000	51.7	75.0

Mahasamund	41.4	0.001	17.7	65.1	69.1	0.000	44.9	93.4	56.0	0.000	38.7	73.2
Dhamtari	62.7	0.000	39.9	85.5	69.2	0.000	46.7	91.8	66.0	0.000	47.6	84.3
Uttar bastar kanker	53.2	0.000	24.4	82.0	85.7	0.000	56.2	115.2	70.1	0.000	50.6	89.6
Bastar	71.4	0.000	49.5	93.4	119.3	0.000	91.7	146.9	95.9	0.000	77.1	114.6
Narayanpur	58.0	0.000	38.2	77.9	86.4	0.000	67.4	105.3	73.0	0.000	57.3	88.7
Dakshin bastar dantewada	102.6	0.000	75.5	129.7	110.4	0.000	82.5	138.4	106.6	0.000	88.5	124.7
Bijapur	54.2	0.000	35.7	72.7	67.6	0.000	50.0	85.2	61.2	0.000	46.7	75.7
Sheopur	82.2	0.000	54.8	109.7	86.1	0.000	61.4	110.7	84.2	0.000	64.1	104.4
Morena	89.3	0.000	67.1	111.4	68.0	0.000	44.6	91.4	77.8	0.000	60.6	95.1
Bhind	75.0	0.000	48.8	101.2	62.7	0.000	43.2	82.2	68.6	0.000	52.0	85.1
Gwalior	71.0	0.000	50.0	92.0	69.3	0.000	53.0	85.7	70.2	0.000	56.6	83.7
Datia	93.4	0.000	66.7	120.1	87.7	0.000	63.4	112.0	90.5	0.000	73.8	107.2
Shivpuri	78.2	0.000	53.4	103.1	76.6	0.000	57.8	95.5	77.3	0.000	61.3	93.3
Tikamgarh	106.4	0.000	74.8	138.1	73.4	0.000	50.8	96.1	89.8	0.000	69.0	110.6
Chhatarpur	88.2	0.000	61.7	114.7	78.1	0.000	55.0	101.3	82.9	0.000	64.2	101.7
Panna	123.4	0.000	93.1	153.7	113.2	0.000	80.9	145.5	117.8	0.000	97.4	138.2
Sagar	66.8	0.000	39.4	94.2	81.2	0.000	53.6	108.7	74.9	0.000	57.6	92.1
Damoh	73.9	0.000	44.6	103.2	77.0	0.000	53.8	100.1	75.6	0.000	55.1	96.2
Satna	76.0	0.000	47.7	104.3	65.0	0.000	40.1	90.0	70.6	0.000	56.0	85.3
Rewa	105.3	0.000	76.0	134.7	110.1	0.000	81.6	138.7	107.8	0.000	87.1	128.5
Umaria	66.8	0.000	43.2	90.4	77.4	0.000	43.7	111.2	72.2	0.000	51.6	92.9
Neemuch	41.3	0.000	21.9	60.7	52.5	0.000	31.2	73.7	47.1	0.000	30.4	63.8
Mandsaur	57.2	0.000	33.0	81.4	65.3	0.000	42.6	87.9	61.5	0.000	44.2	78.7
Ratlam	34.4	0.001	14.5	54.3	39.8	0.000	22.6	57.1	37.2	0.000	28.0	46.5
Ujjain	38.1	0.000	26.6	49.5	64.1	0.000	46.8	81.5	50.8	0.000	40.7	61.0
Shajapur	53.1	0.000	33.2	73.1	45.7	0.000	27.7	63.6	49.3	0.000	33.3	65.4
Dewas	66.4	0.000	43.3	89.4	79.4	0.000	52.1	106.7	72.8	0.000	56.1	89.5
Dhar	60.3	0.000	38.5	82.0	80.0	0.000	53.8	106.2	70.5	0.000	54.5	86.5
Indore	22.0	0.001	8.9	35.1	21.0	0.000	12.6	29.3	21.4	0.000	13.3	29.5
Khargone (west nimar)	54.7	0.000	30.9	78.5	53.1	0.000	31.0	75.2	53.9	0.000	39.2	68.7
Barwani	55.4	0.000	38.0	72.9	55.6	0.000	41.0	70.1	55.6	0.000	43.0	68.1
Rajgarh	88.2	0.000	61.2	115.2	82.8	0.000	55.6	110.0	85.7	0.000	65.6	105.8
Vidisha	60.4	0.000	39.9	80.9	86.9	0.000	56.8	117.0	73.6	0.000	56.4	90.9
Bhopal	51.1	0.000	27.2	74.9	47.5	0.000	28.3	66.6	49.4	0.000	32.5	66.3
Sehore	50.3	0.000	28.9	71.6	49.3	0.000	28.7	70.0	49.8	0.000	37.9	61.7
Raisen	63.6	0.000	41.5	85.8	69.2	0.000	47.0	91.4	66.7	0.000	50.6	82.8
Betul	58.2	0.000	35.1	81.2	81.2	0.000	53.1	109.3	70.6	0.000	55.5	85.6
Harda	58.6	0.000	39.0	78.3	58.3	0.000	38.0	78.7	58.5	0.000	43.3	73.6
Hoshangabad	55.9	0.000	36.8	75.0	56.2	0.000	39.0	73.5	56.1	0.000	44.3	67.9
Katni	73.7	0.000	48.0	99.5	79.2	0.000	55.6	102.8	76.4	0.000	57.5	95.3
Jabalpur	69.4	0.000	47.5	91.4	63.7	0.000	46.0	81.5	66.5	0.000	57.4	75.6
Narsimhapur	43.2	0.000	21.6	64.8	46.7	0.000	21.5	71.9	45.1	0.000	29.6	60.5
Dindori	91.1	0.000	58.5	123.7	86.4	0.000	59.3	113.4	88.8	0.000	64.7	112.9
Mandla	68.5	0.000	42.5	94.5	96.2	0.000	65.4	127.0	82.2	0.000	61.5	102.9
Chhindwara	73.1	0.000	46.5	99.8	57.7	0.000	33.8	81.5	65.1	0.000	46.2	84.0
Seoni	63.9	0.000	41.0	86.8	29.1	0.000	14.1	44.1	46.2	0.000	30.8	61.6
Balaghat	70.1	0.000	43.7	96.4	77.4	0.000	50.1	104.7	73.8	0.000	53.8	93.7
Guna	88.7	0.000	60.8	116.7	91.8	0.000	72.4	111.1	90.1	0.000	71.8	108.4
Ashoknagar	44.1	0.000	27.9	60.2	48.3	0.000	32.5	64.0	46.3	0.000	33.9	58.6
Shahdol	82.9	0.000	58.7	107.0	70.9	0.000	50.1	91.8	76.9	0.000	57.8	96.0
Anuppur	68.9	0.000	42.2	95.7	69.1	0.000	47.2	91.0	68.9	0.000	54.0	83.7
Sidhi	94.1	0.000	72.2	116.0	86.5	0.000	62.6	110.5	90.2	0.000	72.2	108.2
Singrauli	69.4	0.000	41.2	97.7	89.2	0.000	64.5	113.9	79.5	0.000	65.5	93.5
Jhabua	52.1	0.000	32.4	71.9	64.6	0.000	46.6	82.7	58.4	0.000	45.1	71.7
Alirajpur	71.9	0.000	52.8	91.0	92.9	0.000	74.5	111.3	82.7	0.000	69.7	95.7
Khandwa (east nimar)	51.0	0.000	30.0	72.0	55.1	0.000	36.8	73.5	53.2	0.000	38.6	67.8
Burhanpur	57.2	0.000	41.3	73.0	70.7	0.000	55.9	85.5	64.5	0.000	51.4	77.5
Kachchh	36.5	0.001	15.9	57.1	60.3	0.000	38.0	82.6	49.0	0.000	31.2	66.8
Banaskantha	56.0	0.000	35.5	76.6	72.6	0.000	46.3	98.9	64.6	0.000	48.0	81.2
Patan	31.7	0.000	14.4	49.0	56.8	0.000	29.7	83.8	45.7	0.000	28.4	62.9
Mahesana	24.2	0.029	2.4	46.0	48.8	0.000	24.5	73.1	37.5	0.000	22.1	52.8
Sabarkantha	48.1	0.000	24.1	72.2	58.9	0.000	34.9	82.8	53.6	0.000	35.0	72.3
Gandhinagar	45.2	0.004	14.7	75.6	57.1	0.000	28.4	85.8	51.0	0.000	31.0	71.0
Ahmadabad	21.7	0.015	4.2	39.1	48.0	0.000	25.2	70.8	35.9	0.000	15.8	55.9
Surendranagar	32.0	0.002	11.7	52.3	42.1	0.000	18.5	65.6	37.0	0.000	22.8	51.2
Rajkot	32.9	0.012	7.2	58.5	28.9	0.008	7.5	50.4	30.7	0.000	15.2	46.1
Jamnagar	29.8	0.004	9.4	50.1	31.3	0.000	13.8	48.7	30.4	0.000	14.2	46.6
Porbandar	21.8	0.016	4.1	39.5	23.8	0.001	9.2	38.4	22.9	0.000	10.1	35.7
Junagadh	41.7	0.005	12.7	70.7	36.2	0.008	9.6	62.7	38.7	0.000	20.9	56.5
Amreli	22.1	0.039	1.1	43.1	15.2	0.061	-0.7	31.0	18.1	0.005	5.4	30.9
Bhavnagar	40.4	0.000	17.7	63.1	38.4	0.000	18.2	58.6	39.4	0.000	23.6	55.2
Anand	87.2	0.000	43.5	130.9	78.4	0.000	38.3	118.5	82.7	0.000	55.5	110.0
Kheda	57.2	0.000	30.8	83.6	71.5	0.000	39.7	103.4	64.7	0.000	48.1	81.2
Panchmahal	48.1	0.000	26.5	69.7	51.2	0.000	30.2	72.1	49.7	0.000	36.2	63.3
Dohad	77.1	0.000	54.9	99.3	97.0	0.000	62.0	132.0	87.4	0.000	66.9	108.0
Vadodara	40.3	0.002	15.0	65.5	52.7	0.000	29.6	75.8	46.4	0.000	30.4	62.4
Narmada	70.7	0.000	45.7	95.6	72.6	0.000	44.4	100.7	71.6	0.000	52.1	91.1
Bharuch	45.0	0.000	25.0	65.1	92.0	0.000	53.4	130.5	68.1	0.000	45.3	90.8
The dangs	46.1	0.000	29.5	62.6	36.9	0.000	16.8	56.9	41.2	0.000	27.0	55.3
Navsari	35.9	0.003	11.9	60.0	46.3	0.000	21.5	71.1	41.2	0.000	19.5	63.0
Valsad	20.8	0.020	3.3	38.3	47.1	0.000	21.1	73.2	34.0	0.000	19.8	48.2
Surat	19.8	0.020	3.1	36.5	6.4	0.198	-3.3	16.1	12.6	0.013	2.6	22.5
Tapi	34.2	0.003	11.3	57.1	19.0	0.019	3.2	34.9	26.6	0.000	13.1	40.0

Diu	17.1	0.085	-2.4	36.5	10.4	0.057	-0.3	21.2	13.5	0.022	1.9	25.0
Daman	22.4	0.009	5.6	39.2	50.7	0.000	23.1	78.3	37.4	0.000	18.4	56.5
Dadra & nagar haveli	23.4	0.014	4.7	42.2	45.9	0.000	24.6	67.1	35.0	0.000	22.4	47.6
Nandurbar	18.4	0.001	7.9	28.9	33.1	0.000	14.9	51.3	25.7	0.000	11.5	40.0
Dhule	17.4	0.008	4.6	30.2	19.8	0.004	6.3	33.2	18.7	0.000	9.1	28.3
Jalgaon	39.4	0.003	13.7	65.1	36.5	0.000	17.8	55.3	37.9	0.000	18.8	57.0
Buldana	28.9	0.001	12.0	45.8	28.4	0.002	10.8	46.0	28.6	0.000	12.8	44.4
Akola	32.9	0.001	12.7	53.2	35.1	0.001	14.6	55.7	34.1	0.000	19.1	49.2
Washim	25.3	0.000	11.3	39.4	20.1	0.014	4.0	36.2	22.5	0.000	10.3	34.7
Amravati	14.5	0.080	-1.7	30.6	26.8	0.046	0.4	53.2	20.6	0.004	6.7	34.4
Wardha	2.7	0.334	-2.8	8.2	65.0	0.000	30.0	100.0	33.9	0.000	19.1	48.7
Nagpur	30.2	0.040	1.3	59.1	43.8	0.025	5.5	82.0	37.1	0.001	15.9	58.3
Bhandara	24.6	0.012	5.4	43.9	49.8	0.000	28.4	71.1	37.2	0.000	18.6	55.8
Gondiya	40.0	0.001	15.4	64.7	29.8	0.007	8.3	51.4	35.2	0.000	20.6	49.8
Gadchiroli	14.9	0.050	0.0	29.8	46.5	0.001	18.2	74.8	31.4	0.000	14.1	48.7
Chandrapur	22.4	0.082	-2.8	47.6	38.2	0.007	10.6	65.8	30.1	0.002	11.0	49.2
Yavatmal	14.4	0.057	-0.4	29.1	40.8	0.000	18.1	63.5	28.6	0.001	12.3	44.9
Nanded	45.1	0.000	23.5	66.7	48.8	0.000	27.4	70.1	47.1	0.000	32.9	61.3
Hingoli	49.4	0.000	25.3	73.5	39.6	0.000	17.6	61.7	44.6	0.000	30.2	58.9
Parbhani	21.9	0.010	5.2	38.6	46.8	0.000	24.3	69.3	35.3	0.000	25.4	45.2
Jalna	22.5	0.001	9.0	36.0	31.1	0.000	13.8	48.5	27.0	0.000	17.2	36.7
Aurangabad	15.5	0.012	3.4	27.6	30.7	0.000	13.7	47.8	23.4	0.000	10.9	35.9
Nashik	18.4	0.010	4.4	32.4	61.6	0.000	27.8	95.4	41.4	0.000	25.5	57.3
Thane	20.0	0.048	0.2	39.8	27.0	0.016	5.0	49.0	23.6	0.000	10.5	36.7
Mumbai suburban	38.3	0.027	4.3	72.2	37.7	0.048	0.3	75.0	38.2	0.001	16.5	59.9
Mumbai	28.4	0.059	-1.1	57.8	23.7	0.091	-3.8	51.2	26.1	0.015	5.1	47.2
Raigarh	40.5	0.015	7.8	73.2	20.2	0.048	0.2	40.2	30.0	0.000	14.6	45.3
Pune	19.2	0.102	-3.8	42.1	26.3	0.012	5.8	46.9	23.0	0.002	8.2	37.7
Ahmadnagar	10.6	0.045	0.3	21.0	12.4	0.066	-0.8	25.7	11.6	0.008	3.0	20.3
Bid	19.8	0.009	4.8	34.8	21.4	0.018	3.6	39.1	20.6	0.000	10.1	31.1
Latur	20.2	0.004	6.4	33.9	32.8	0.000	14.8	50.8	27.0	0.000	14.5	39.6
Osmanabad	21.8	0.030	2.1	41.5	36.2	0.000	17.3	55.2	29.6	0.000	16.3	42.9
Solapur	15.5	0.127	-4.4	35.3	49.0	0.000	23.0	75.1	34.2	0.002	12.6	55.8
Satara	24.3	0.084	-3.2	51.8	22.3	0.025	2.8	41.8	23.2	0.001	9.0	37.5
Ratnagiri	18.0	0.049	0.1	35.8	37.1	0.000	16.6	57.5	27.8	0.001	11.8	43.8
Sindhudurg	31.1	0.021	4.7	57.5	29.6	0.031	2.6	56.6	30.2	0.001	11.6	48.8
Kolhapur	5.3	0.224	-3.3	13.9	28.5	0.014	5.9	51.2	18.7	0.010	4.4	33.0
Sangli	26.3	0.038	1.4	51.1	32.9	0.001	13.2	52.6	30.0	0.000	16.0	44.1
Adilabad	64.9	0.000	29.4	100.3	45.8	0.001	19.2	72.4	54.7	0.000	33.6	75.9
Nizamabad	56.7	0.000	31.5	82.0	76.3	0.000	42.3	110.3	67.9	0.000	44.9	90.8
Karimnagar	39.0	0.006	11.1	67.0	47.8	0.000	23.5	72.0	43.4	0.000	21.9	64.9
Medak	45.2	0.002	16.7	73.6	45.1	0.000	19.8	70.3	45.1	0.000	28.4	61.8
Hyderabad	27.1	0.020	4.2	49.9	25.6	0.046	0.5	50.8	26.3	0.004	8.2	44.5
Rangareddy	25.3	0.029	2.6	48.1	49.4	0.000	26.3	72.4	38.1	0.000	20.8	55.5
Mahbubnagar	58.7	0.000	39.0	78.4	83.7	0.000	49.1	118.2	71.4	0.000	50.5	92.4
Nalgonda	27.0	0.024	3.5	50.6	44.8	0.000	20.6	69.0	36.4	0.000	20.8	52.1
Warangal	27.8	0.016	5.2	50.5	14.9	0.079	-1.7	31.5	21.4	0.002	7.8	34.9
Khammam	29.6	0.018	5.1	54.0	58.0	0.002	21.6	94.4	44.2	0.000	26.0	62.3
Srikakulam	52.2	0.001	22.8	81.6	61.5	0.000	34.8	88.2	57.2	0.000	33.4	81.0
Vizianagaram	56.3	0.000	27.0	85.7	55.5	0.000	31.5	79.5	56.1	0.000	38.5	73.7
Visakhapatnam	28.7	0.050	0.0	57.3	55.5	0.005	16.8	94.1	42.1	0.000	22.7	61.5
East godavari	57.1	0.001	22.6	91.6	56.0	0.000	26.4	85.6	56.2	0.000	32.9	79.6
West godavari	44.3	0.001	18.2	70.4	33.2	0.004	10.4	56.0	38.6	0.000	22.8	54.4
Krishna	59.1	0.002	21.5	96.7	18.4	0.051	-0.1	36.9	37.6	0.000	16.6	58.6
Guntur	50.0	0.002	17.8	82.2	53.3	0.001	22.5	84.1	52.2	0.000	29.1	75.3
Prakasam	66.6	0.001	29.1	104.2	67.1	0.000	39.5	94.6	66.9	0.000	46.8	86.9
Sri potti sriramulu nellore	12.6	0.097	-2.3	27.5	30.1	0.003	10.1	50.1	21.1	0.001	8.3	33.9
Y.s.r.	29.2	0.014	5.9	52.6	47.2	0.000	24.5	69.9	39.7	0.000	19.2	60.2
Kurnool	57.2	0.000	30.0	84.4	69.7	0.000	47.4	92.1	63.9	0.000	47.5	80.4
Anantapur	37.2	0.001	14.3	60.2	62.5	0.001	25.9	99.2	50.0	0.000	31.1	68.8
Chittoor	37.0	0.002	13.9	60.0	49.7	0.003	16.6	82.9	43.2	0.000	24.6	61.9
Belgaum	20.8	0.007	5.7	35.8	31.9	0.001	12.7	51.2	26.5	0.000	14.2	38.7
Bagalkot	32.8	0.000	18.1	47.6	33.3	0.001	12.8	53.8	33.1	0.000	17.2	48.9
Bijapur	33.3	0.000	15.6	51.0	20.1	0.008	5.3	34.9	26.5	0.000	16.9	36.0
Bidar	17.2	0.008	4.5	29.8	42.0	0.000	19.6	64.4	29.2	0.000	16.8	41.7
Raichur	34.0	0.001	14.3	53.7	52.2	0.000	30.3	74.2	43.4	0.000	29.4	57.3
Koppal	23.0	0.004	7.2	38.7	33.4	0.000	16.1	50.7	28.2	0.000	17.3	39.1
Gadag	10.8	0.239	-7.2	28.8	24.4	0.071	-2.1	50.9	18.3	0.014	3.7	32.9
Dharwad	47.9	0.000	25.2	70.6	38.0	0.000	18.4	57.7	42.8	0.000	23.6	62.0
Uttara kannada	26.5	0.007	7.3	45.7	20.7	0.038	1.1	40.4	23.6	0.001	9.8	37.4
Haveri	17.4	0.037	1.0	33.8	20.7	0.017	3.6	37.8	19.1	0.005	5.6	32.5
Bellary	44.7	0.000	23.2	66.2	31.2	0.000	14.5	47.9	37.2	0.000	22.7	51.8
Chitradurga	32.8	0.008	8.6	57.0	52.9	0.000	31.5	74.4	43.8	0.000	26.3	61.2
Davanagere	24.8	0.013	5.2	44.4	62.3	0.000	33.8	90.8	43.8	0.000	25.7	62.0
Shimoga	39.5	0.001	16.8	62.3	49.9	0.002	18.2	81.6	44.7	0.000	24.8	64.7
Udupi	17.8	0.070	-1.4	37.1	14.1	0.121	-3.7	31.8	15.8	0.004	4.9	26.7
Chikmagalur	18.2	0.126	-5.1	41.6	19.7	0.038	1.1	38.3	18.9	0.013	4.1	33.8
Tumkur	62.6	0.000	34.3	90.9	62.6	0.000	29.2	95.9	62.5	0.000	43.2	81.9
Bangalore	17.5	0.046	0.3	34.6	15.2	0.046	0.3	30.1	16.2	0.007	4.3	28.0
Mandya	36.7	0.008	9.5	63.8	29.1	0.030	2.8	55.4	32.6	0.001	12.5	52.6
Hassan	27.0	0.019	4.5	49.5	53.4	0.001	21.3	85.6	40.4	0.000	22.4	58.5
Dakshina kannada	31.6	0.004	10.0	53.3	19.6	0.042	0.7	38.5	25.5	0.001	10.1	40.9
Kodagu	0.0	0.000	0.0	0.0	30.1	0.004	9.5	50.7	14.9	0.002	5.5	24.3

Mysore	39.7	0.005	11.7	67.7	30.6	0.008	8.0	53.2	35.0	0.000	19.6	50.4
Chamarajanagar	43.0	0.001	17.6	68.4	66.8	0.000	34.2	99.5	54.5	0.000	32.3	76.7
Gulbarga	17.4	0.016	3.3	31.5	29.8	0.000	13.4	46.2	23.9	0.000	13.0	34.7
Yadgir	21.9	0.002	8.1	35.7	29.3	0.001	11.8	46.8	25.9	0.000	15.6	36.1
Kolar	45.6	0.001	18.7	72.5	39.6	0.001	15.2	64.0	42.3	0.000	23.9	60.7
Chikkaballapura	36.5	0.007	10.1	62.9	29.4	0.008	7.8	51.0	32.8	0.000	15.3	50.4
Bangalore rural	22.3	0.022	3.3	41.3	47.6	0.002	16.9	78.4	34.7	0.000	19.7	49.6
Ramanagara	34.4	0.014	6.9	62.0	36.9	0.005	11.1	62.8	35.7	0.000	18.7	52.8
North goa	11.3	0.056	-0.3	22.9	14.7	0.021	2.3	27.2	13.2	0.001	5.2	21.1
South goa	27.5	0.044	0.7	54.3	17.7	0.064	-1.1	36.5	22.6	0.011	5.1	40.0
Lakshadweep	33.6	0.002	12.3	54.9	20.9	0.008	5.5	36.4	27.0	0.000	13.2	40.8
Kasaragod	0.0	0.000	0.0	0.0	6.9	0.278	-5.6	19.4	3.7	0.158	-1.4	8.9
Kannur	6.5	0.222	-3.9	16.9	13.6	0.117	-3.4	30.6	10.0	0.026	1.2	18.9
Wayanad	19.1	0.008	5.1	33.2	19.1	0.076	-2.0	40.2	19.0	0.001	8.1	30.0
Kozhikode	5.1	0.304	-4.6	14.9	8.6	0.219	-5.1	22.4	7.0	0.114	-1.7	15.7
Malappuram	3.3	0.347	-3.6	10.1	2.9	0.351	-3.2	8.9	3.1	0.235	-2.0	8.1
Palakkad	0.0	0.000	0.0	0.0	7.2	0.129	-2.1	16.5	3.9	0.114	-1.0	8.8
Thrissur	0.0	0.000	0.0	0.0	0.0	0.000	0.0	0.0	0.0	0.000	0.0	0.0
Ernakulam	0.0	0.000	0.0	0.0	13.3	0.142	-4.5	31.0	6.6	0.174	-2.9	16.0
Idukki	5.6	0.371	-6.6	17.8	8.9	0.220	-5.3	23.2	7.2	0.071	-0.6	15.0
Kottayam	5.8	0.344	-6.2	17.9	6.0	0.326	-6.0	18.0	5.9	0.212	-3.4	15.2
Alappuzha	31.9	0.078	-3.5	67.3	5.9	0.300	-5.3	17.2	19.1	0.007	5.2	33.1
Pathanamthitta	14.4	0.076	-1.5	30.3	21.0	0.087	-3.0	45.1	17.8	0.021	2.7	32.9
Kollam	4.8	0.413	-6.8	16.5	18.4	0.085	-2.5	39.2	12.2	0.084	-1.6	26.0
Thiruvananthapuram	0.0	0.000	0.0	0.0	19.5	0.023	2.7	36.3	9.6	0.028	1.0	18.1
Thiruvallur	22.0	0.021	3.4	40.6	39.2	0.006	11.1	67.3	31.2	0.000	15.5	46.9
Chennai	2.5	0.105	-0.5	5.6	13.1	0.351	-14.4	40.5	7.3	0.219	-4.3	18.9
Kancheepuram	37.2	0.027	4.3	70.1	14.2	0.027	1.6	26.7	25.2	0.004	8.0	42.5
Vellore	28.9	0.011	6.5	51.2	24.0	0.002	8.8	39.2	26.4	0.000	11.9	40.8
Tiruvannamalai	22.5	0.036	1.5	43.6	29.4	0.006	8.3	50.5	26.2	0.000	11.5	40.8
Viluppuram	28.2	0.002	10.4	45.9	37.4	0.014	7.6	67.1	33.0	0.000	18.6	47.3
Salem	4.3	0.277	-3.4	12.0	30.4	0.005	9.4	51.4	17.8	0.000	8.0	27.6
Namakkal	10.8	0.036	0.7	20.8	26.9	0.039	1.3	52.5	18.8	0.003	6.4	31.3
Erode	10.6	0.163	-4.3	25.4	9.9	0.212	-5.6	25.4	10.4	0.037	0.6	20.1
The nilgiris	10.8	0.113	-2.6	24.3	17.8	0.069	-1.4	36.9	14.4	0.015	2.8	26.0
Dindigul	27.7	0.011	6.2	49.1	38.0	0.003	12.6	63.3	33.1	0.001	13.8	52.4
Karur	28.0	0.021	4.2	51.9	16.4	0.052	-0.2	33.1	22.0	0.003	7.5	36.5
Tiruchirappalli	41.4	0.004	13.0	69.9	39.8	0.005	12.0	67.5	40.7	0.000	21.7	59.6
Perambalur	8.5	0.175	-3.8	20.8	44.4	0.005	13.6	75.2	28.1	0.000	13.1	43.2
Ariyalur	16.3	0.082	-2.1	34.8	21.8	0.035	1.6	42.0	19.4	0.001	7.9	30.9
Cuddalore	23.9	0.030	2.4	45.5	34.4	0.000	15.0	53.8	29.9	0.000	17.9	42.0
Nagapattinam	40.2	0.000	20.3	60.1	25.1	0.010	6.1	44.1	32.6	0.000	19.6	45.7
Thiruvarur	23.8	0.016	4.4	43.2	23.3	0.045	0.5	46.0	23.5	0.005	7.3	39.7
Thanjavur	29.0	0.030	2.9	55.1	45.4	0.004	14.7	76.1	38.0	0.000	19.3	56.6
Pudukkottai	14.6	0.028	1.5	27.7	19.6	0.010	4.6	34.6	17.1	0.003	5.8	28.4
Sivaganga	40.5	0.001	17.0	64.0	14.9	0.142	-5.0	34.7	28.7	0.000	13.9	43.5
Madurai	14.6	0.043	0.4	28.7	28.0	0.011	6.4	49.6	21.6	0.000	10.3	32.9
Theni	15.3	0.070	-1.2	31.8	39.5	0.011	9.1	69.9	27.8	0.001	10.9	44.6
Virudhunagar	30.0	0.014	6.1	53.9	18.7	0.042	0.7	36.8	24.3	0.003	8.1	40.6
Ramanathapuram	7.0	0.045	0.2	13.9	16.6	0.040	0.8	32.4	12.0	0.004	3.8	20.3
Thoothukkudi	13.6	0.108	-3.0	30.2	30.1	0.036	2.0	58.1	21.4	0.000	9.7	33.2
Tirunelveli	28.7	0.021	4.4	53.0	53.9	0.001	21.0	86.9	41.3	0.000	23.5	59.0
Kanniyakumari	21.8	0.063	-1.2	44.7	25.1	0.015	4.8	45.3	23.5	0.001	9.0	37.9
Dharmapuri	13.9	0.062	-0.7	28.4	25.0	0.014	5.2	44.9	19.5	0.003	6.5	32.6
Krishnagiri	27.7	0.005	8.2	47.3	52.6	0.000	27.3	77.8	40.6	0.001	17.5	63.7
Coimbatore	10.7	0.243	-7.2	28.6	26.4	0.005	7.9	45.0	19.0	0.000	8.5	29.5
Tiruppur	19.5	0.022	2.9	36.2	38.0	0.003	12.5	63.4	28.6	0.000	12.8	44.4
Yanam	20.8	0.024	2.8	38.7	33.1	0.001	13.6	52.6	27.3	0.000	12.2	42.4
Puducherry	12.9	0.064	-0.7	26.6	30.9	0.002	11.5	50.3	22.5	0.006	6.6	38.3
Mahe	3.8	0.251	-2.7	10.3	10.1	0.094	-1.7	21.9	6.9	0.028	0.8	13.1
Karaikal	28.9	0.002	10.4	47.3	29.3	0.006	8.6	50.1	29.0	0.000	13.6	44.4
Nicobars	10.6	0.051	0.0	21.2	7.1	0.128	-2.0	16.3	8.9	0.022	1.3	16.5
North & middle andaman	17.7	0.023	2.4	33.0	19.5	0.021	2.9	36.1	18.6	0.002	6.9	30.4
South andaman	8.2	0.174	-3.6	20.1	13.7	0.032	1.2	26.3	11.1	0.018	1.9	20.2
**India**	**50.4**	**0.000**	**49.3**	**51.5**	**53.8**	**0.000**	**52.6**	**55.0**	**52.2**	**0.000**	**51.3**	**53.0**
